# The impact of traditional Chinese medicine on gastrointestinal dysfunction in sepsis patients: a systematic review and network meta-analysis

**DOI:** 10.3389/fphar.2025.1648809

**Published:** 2025-10-10

**Authors:** Ziyi Sang, Yuchao Wang, Caixia Wu, Jiangtao Yin

**Affiliations:** ^1^ College of Excellence in Medical Education, Jiangsu University, Zhenjiang, Jiangsu, China; ^2^ Department of Critical Care Medicine, Digestive Disease Institute of Jiangsu University, Affiliated Hospital of Jiangsu University, Zhenjiang, China

**Keywords:** traditional Chinese medicine, sepsis, gastrointestinal dysfunction, network meta-analysis, system review

## Abstract

**Objective:**

This study aimed to evaluate, via a network meta-analysis, the efficacy and comparative differences of 16 traditional Chinese medicines—including Dachaihu Tang, Tiaoqi Tongfu Tang, Yiqi formulations, Dahuang Fuzi Tang, and Xuebijing—in treating gastrointestinal dysfunction in patients with sepsis. Effect sizes were quantified across outcome measures such as gastrointestinal dysfunction scores, traditional Chinese medicine syndrome scores, APACHE II scores, AGI grades, mean levels of D-lactate, diamine oxidase (DAO), tumor necrosis factor-alpha (TNF-α), interleukin-6 (IL-6), and intra-abdominal pressure (IAP), with the aim of providing evidence-based recommendations for precise clinical medication.

**Methods:**

Randomized controlled trials assessing the efficacy of various traditional Chinese medicines in patients with sepsis-induced gastrointestinal dysfunction were retrieved by systematically searching PubMed, Embase, Cochrane Library, Web of Science, and CNKI.

**Results:**

Traditional Chinese medicines were overall more effective than conventional treatments in improving gastrointestinal function, suppressing inflammatory responses, and repairing the intestinal mucosal barrier in patients with sepsis. Among the evaluated formulas, Dachaihu Tang showed the most significant improvement in gastrointestinal dysfunction scores [MD = −2.03, 95% CI: −3.86 to −0.20], while Yiqi formulations exhibited the most favorable effect on traditional Chinese medicine syndrome scores [MD = −5.70, 95% CI: −9.84 to −1.56]. Dahuang Fuzi Tang was most effective in improving AGI grade [MD = 0.27, 95% CI: 0.19 to 0.40], and Tiaoqi Tongfu Tang had the strongest effect on reducing IAP [MD = −4.93, 95% CI: −9.09 to −0.77]. In terms of inflammatory markers, Tiaoqi Tongfu Tang significantly reduced APACHE II scores [MD = −6.90, 95% CI: −9.66 to −4.13] and TNF-α levels [MD = −7.65, 95% CI: −10.27 to −5.04], while Houpo Heji had the most potent regulatory effect on IL-6 [MD = −4.24, 95% CI: −5.34 to −3.14]. Regarding intestinal mucosal barrier repair, Xuebijing was most effective in reducing D-lactate levels [MD = −3.51, 95% CI: −6.73 to −0.29], and had the greatest effect on improving DAO levels [MD = −4.57, 95% CI: −7.86 to −1.28].

**Conclusion:**

This study provides an evidence-based reference for clinical decision-making. Different traditional Chinese medicines demonstrated distinct advantages in the treatment of gastrointestinal dysfunction associated with sepsis. It is recommended that clinical interventions be selected based on core pathological features: Dachaihu Tang for gastrointestinal motility disorders, Tiaoqi Tongfu Tang and Houpo Heji for prominent systemic inflammatory responses, and Xuebijing for severe mucosal barrier damage. All of these treatments showed significant superiority over conventional therapies. These findings require further validation through longer-duration, larger-sample, and higher-quality randomized controlled trials.

**Systematic review registration:**

https://www.crd.york.ac.uk/PROSPERO/view/CRD420251062621, identifier CRD420251062621.

## 1 Introduction

Sepsis is a life-threatening syndrome characterized by a systemic inflammatory response triggered by infection. Due to its acute onset, rapid progression, and high mortality rate, its management has become a major challenge in critical care medicine worldwide (Singer et al., 2016; Cecconi et al., 2018). The key pathophysiological features of sepsis include uncontrolled systemic inflammation, immune dysfunction, and multiple organ failure, with gastrointestinal dysfunction being one of the principal driving factors (Adelman et al., 2020). Gastrointestinal dysfunction in sepsis not only leads to impaired nutrient absorption and microbial dysbiosis but also causes damage to the intestinal mucosal barrier, facilitating bacterial and endotoxin translocation, thereby significantly increasing treatment complexity and mortality risk (Tang et al., 2022). Current clinical interventions for sepsis-related gastrointestinal dysfunction include early enteral nutrition, prokinetic agents, and probiotics for gut microbiota regulation (Vincent, 2022). However, these approaches remain limited in efficacy. For instance, enteral nutrition is often discontinued due to poor patient tolerance (Patel et al., 2021), and the effects of prokinetic agents are highly variable across individuals (Wang and Camilleri, 2019). Moreover, while Biomedicine can partially control disease progression, they are frequently associated with safety concerns. Antibiotics, in particular, may lead to antimicrobial resistance and gut flora imbalance (Lankelma et al., 2017), and are often accompanied by adverse effects such as nausea and vomiting (Wang and Camilleri, 2019). Studies have shown that sepsis patients treated solely with conventional biomedicine still face a 28-day mortality rate as high as 33.8%, prolonged ICU stays, and significantly increased healthcare costs (Torio and Andrews, 2006; Liang et al., 2015). These challenges highlight the urgent need to explore safer and more effective therapeutic strategies.

In contrast, traditional Chinese medicine (TCM) demonstrates unique advantages in both safety and efficacy. Through integrative regulation via multi-metabolite, multi-target, and multi-pathway mechanisms, TCM not only alleviates clinical symptoms of gastrointestinal dysfunction in sepsis but also enhances overall therapeutic outcomes, with a typically lower incidence of adverse effects compared to biomedicine (Song et al., 2023). Specifically, TCM interventions may reverse sepsis-associated gastrointestinal dysmotility, mucosal damage, and bacterial translocation by promoting intestinal peristalsis, regulating gut microbiota balance, and improving intestinal microcirculation (Zhang et al., 2021). For example, Rheum palmatum (Da Huang) has been shown to significantly alleviate toxic paralytic ileus, improve gastrointestinal functional recovery rates, and reduce serum endotoxin levels and inflammatory cytokine concentrations (Chen and Wang, 2009). Likewise, Banxia Xiexin Tang has been reported to relieve abdominal pain, distension, nausea, and vomiting by regulating gastrointestinal hormone secretion and enhancing gut motility. It also significantly lowers dysbiosis scores, promotes the proliferation of beneficial bacteria, and suppresses the overgrowth of pathogenic species (Dai et al., 2025). Although numerous TCM formulations have demonstrated beneficial effects on sepsis-induced gastrointestinal dysfunction, the absence of standardized efficacy evaluation criteria and the unclear comparative effectiveness among these agents pose challenges for optimal clinical decision-making.

Network meta-analysis (NMA) is an evidence-based method that integrates data from multiple sources to enable both direct and indirect comparisons across various interventions, thereby allowing quantitative ranking (Higgins and Welton, 2015). By constructing a network of interventions, this method systematically evaluates their relative efficacy and safety, effectively addressing the challenge of insufficient head-to-head comparisons among multiple therapeutic strategies. NMA provides high-level evidence for the comprehensive assessment of complex interventions (Sterne et al., 2019). This study aims to clarify the therapeutic effects and comparative efficacy of various TCM formulations in treating sepsis-induced gastrointestinal dysfunction using network meta-analysis. It also seeks to quantify their effects in reducing inflammatory markers such as IL-6 and TNF-α, as well as improving intestinal function, thereby providing evidence-based support for clinical decision-making and promoting the standardized integration of TCM into the comprehensive treatment of sepsis.

## 2 Materials and methods

This meta-analysis was conducted in accordance with the methodological guidance outlined in the Cochrane Handbook for Systematic Reviews of Interventions and the Preferred Reporting Items for Systematic Reviews and Meta-Analyses (PRISMA) statement. The protocol was prospectively registered in the international PROSPERO database under the registration number CRD420251062621.

### 2.1 Search strategy

A systematic literature search was conducted across five electronic databases—PubMed, EMBASE, the Cochrane Central Register of Controlled Trials (CENTRAL), Web of Science, and CNKI—from database inception to April 2025. The search strategy was structured based on the PICOS framework: (P) Population: patients with sepsis; (I) Intervention: traditional Chinese medicine; (C) Comparator: control group receiving conventional treatment only; (O) Outcome: improvement of gastrointestinal dysfunction in sepsis; and (S) Study design: randomized controlled trials. Two reviewers independently conducted the search. The detailed PICOTS table, including patient characteristics, interventions, comparators, outcomes, timing, and study setting, is provided in Supplementary Table S1. The detailed search strategy for five electronic databases is presented in Supplementary Tables S2A–D as an example.

### 2.2 Botanical identification and herbal nomenclature

All Chinese herbal medicines involved in the included studies were taxonomically validated to ensure the accuracy of plant nomenclature. The accepted botanical names, families, authorities, and pharmacopeial drug names (if available) were confirmed using the Medicinal Plant Names Services and Plants of the World Online databases.

The interventions included in this study are summarized in Supplementary Table S3, which presents representative formulas for each category, reflecting the core structure and therapeutic rationale. The interventions included are as follows:

Banxia Xiexin Tang [Pinelliae Decoction for Purging Heart Fire]
Intestinal Comfort [A proprietary medicine, no standard Latin name]
Rheum palmatum L. [Polygonaceae; Rhei radix et rhizoma] (Common name: Rhubarb)
Dachaihu Tang [Major Bupleurum Decoction]
Dachengqi Tang [Major Purgative Decoction]
Dahuang Fuzi Tang [Rhubarb and Aconite Decoction]
Dahuang Gancao Tang [Rhubarb and Licorice Decoction]
Dahuang Mudan Tang [Rhubarb and Moutan Decoction]
Tiaoqi Tongfu Tang [Qi-Regulating and Fu-Purging Decoction]
Houpo Heji [Officinal Magnolia Beverage]
Sijunzi Tang [Four Gentlemen Decoction]
Liangge San [Cool the Diaphragm Powder]
Xuebijing Injection [Blood Purification Injection] (A proprietary medicine)
Yantiao Fang [A specific formula, no standard Latin name]
Yiqi Tang [Benefit-Qi Decoction]
Zengye Chengqi Tang [Increase Fluids and Purgative Decoction]
Conventional Therapy (as control)

Note: Table X presents representative formulas for each category. Within each category, the composition of individual prescriptions is generally similar; therefore, only representative formulas are shown.

### 2.3 Inclusion criteria

1. The intervention group received various TCM formulations as treatment for sepsis;
2. The control group received conventional treatment only. Conventional treatment included early fluid resuscitation combined with close hemodynamic monitoring (e.g., monitoring of hemodynamic parameters); initial empiric broad-spectrum antimicrobial therapy with prompt switch to pathogen-sensitive antibiotics immediately upon confirmation of the pathogenic microorganism; administration of vasoactive agents where norepinephrine was the first-line choice and vasopressin could be added as an adjunctive agent to achieve the target mean arterial pressure (MAP); comprehensive supportive care encompassing host immune modulation, sedation management, continuous renal replacement therapy (CRRT), and nutritional support; as well as symptomatic treatments tailored to clinical needs, including improvement of intragastric pH value, protection of the gastrointestinal mucosal barrier, and corection of intestinal flora imbalance.
3. Randomized controlled trials (RCTs);
4. Reported at least one of the following outcome measures: gastrointestinal dysfunction scores, TCM syndrome scores, APACHE II scores, AGI grades, D-lactate levels, DAO levels, TNF-α, IL-6, or IAP);
5. No language restrictions were applied.

### 2.4 Exclusion criteria

1. Studies with incomplete data or lacking outcome reporting;
2. Studies that were not RCTs, including quasi-RCTs, animal studies, study protocols, conference abstracts, case reports, or correspondence;
3. Studies not involving the target population, interventions, or outcome measures;
4. Duplicate publications or studies with overlapping data.

### 2.5 Study selection

EndNote software was used for reference management and initial deduplication. Two reviewers independently screened the titles to exclude duplicates, non-randomized studies, reviews, conference abstracts, protocols, and correspondence. Subsequently, the abstracts were independently reviewed by the same two reviewers to identify eligible and ineligible studies. Full texts of the remaining studies were then independently assessed by both reviewers to confirm inclusion. Disagreements were resolved through discussion, and if consensus could not be reached, a third reviewer was consulted for arbitration.

### 2.6 Data extraction

A pre-designed standardized data extraction form was used to collect information from the included studies. The extracted variables included: (1) first author; (2) year of publication; (3) sample size and sex distribution; (4) mean age of participants; (5) interventions and control measures; (6) treatment duration; and (7) primary outcomes, including clinical measures (such as gastrointestinal dysfunction scores, TCM syndrome scores, APACHE II scores, AGI grades) and molecular markers (such as D-lactate, DAO mean, TNF-α, IL-6, and IAP).

### 2.7 Risk of bias of individual studies

Risk of bias (ROB) was independently assessed by two reviewers using the Cochrane Risk of Bias tool (version 2.0, RoB 2.0), as outlined in the Cochrane Handbook for Systematic Reviews of Interventions (version 5.1.0). The assessment covered the following five domains: (1) bias arising from the randomization process; (2) bias due to deviations from intended interventions; (3) bias due to missing outcome data; (4) bias in measurement of the outcome; and (5) bias in selection of the reported result. The overall risk of bias for each study was categorized as low (all domains at low risk), some concerns (at least one domain with some concerns but none at high risk), or high (at least one domain at high risk or multiple domains with issues that substantially lower the credibility of the results) (Sterne et al., 2019).

### 2.8 Data analysis

The study included both continuous and dichotomous variables. Continuous variables were reported as mean ± standard deviation (SD), while dichotomous variables were reported as the number of events. Results for continuous variables were expressed as mean difference (MD) or standardized mean difference (SMD). MD refers to the absolute difference in means between the treatment and control groups and is applicable when outcomes are measured using the same scale. SMD is calculated by dividing the mean difference between groups by the pooled standard deviation, making it suitable for combining trials that use different measurement scales. Both MD and SMD were reported with 95% confidence intervals (CIs). Dichotomous outcomes were expressed as relative risk (RR) or odds ratio (OR). RR is defined as the ratio of event incidence in the treatment group to that in the control group, calculated using data from a 2 × 2 contingency table. OR represents the odds of an event occurring in the exposed group compared to the non-exposed group and reflects the relative strength of association between groups. Both RR and OR were also reported with 95% confidence intervals (CIs). Considering potential heterogeneity among studies, a random-effects model was adopted instead of a fixed-effects model (Puerto Nino and Brignardello-Petersen, 2023).No key outcome data were missing in the included studies; therefore, no imputation or unit conversion was performed. This is clearly stated to ensure transparency in data handling and analysis.

Network meta-analysis was performed using Stata software (version 15.1), in strict accordance with the PRISMA extension statement for network meta-analyses (Moher et al., 2015; Vats et al., 2019). Statistical inference and model synthesis were conducted under a Bayesian framework, using Markov chain Monte Carlo (MCMC) simulation chains. To evaluate consistency between direct and indirect comparisons, node-splitting analysis was employed via built-in routines in Stata. Consistency was considered acceptable if the P-value from the node-splitting analysis exceeded 0.05 (Lee, 2018).

Stata software was used to construct and visualize the network plot of differentTCM interventions interventions. Each node in the plot represents a specificTCM interventions intervention or control condition, and lines connecting nodes indicate the existence of direct head-to-head comparisons between interventions. The size of each node and the thickness of each line are proportional to the number of studies involving that intervention or comparison (Chaimani et al., 2013).

Treatment rankings were summarized and reported using P-scores, which are considered a frequentist analogue of the surface under the cumulative ranking curve (SUCRA). P-scores are used to quantify the certainty that one treatment is superior to another and are calculated based on the mean certainty across all competing interventions. P-scores range from 0 to 1, where a score of 1 indicates the most effective treatment without ambiguity, and a score of 0 represents the least effective. Although P-scores or SUCRA values can be intuitively interpreted as percentage estimates of treatment effectiveness or acceptability, they should be interpreted with caution unless there are clinically meaningful differences among interventions (Marotta et al., 2020). To assess the risk of publication bias potentially arising from small-study effects in the network meta-analysis, network funnel plots were constructed and visually inspected for asymmetry (Khera et al., 2016).

## 3 Results

### 3.1 Study and identification and selection

**FIGURE 1:**
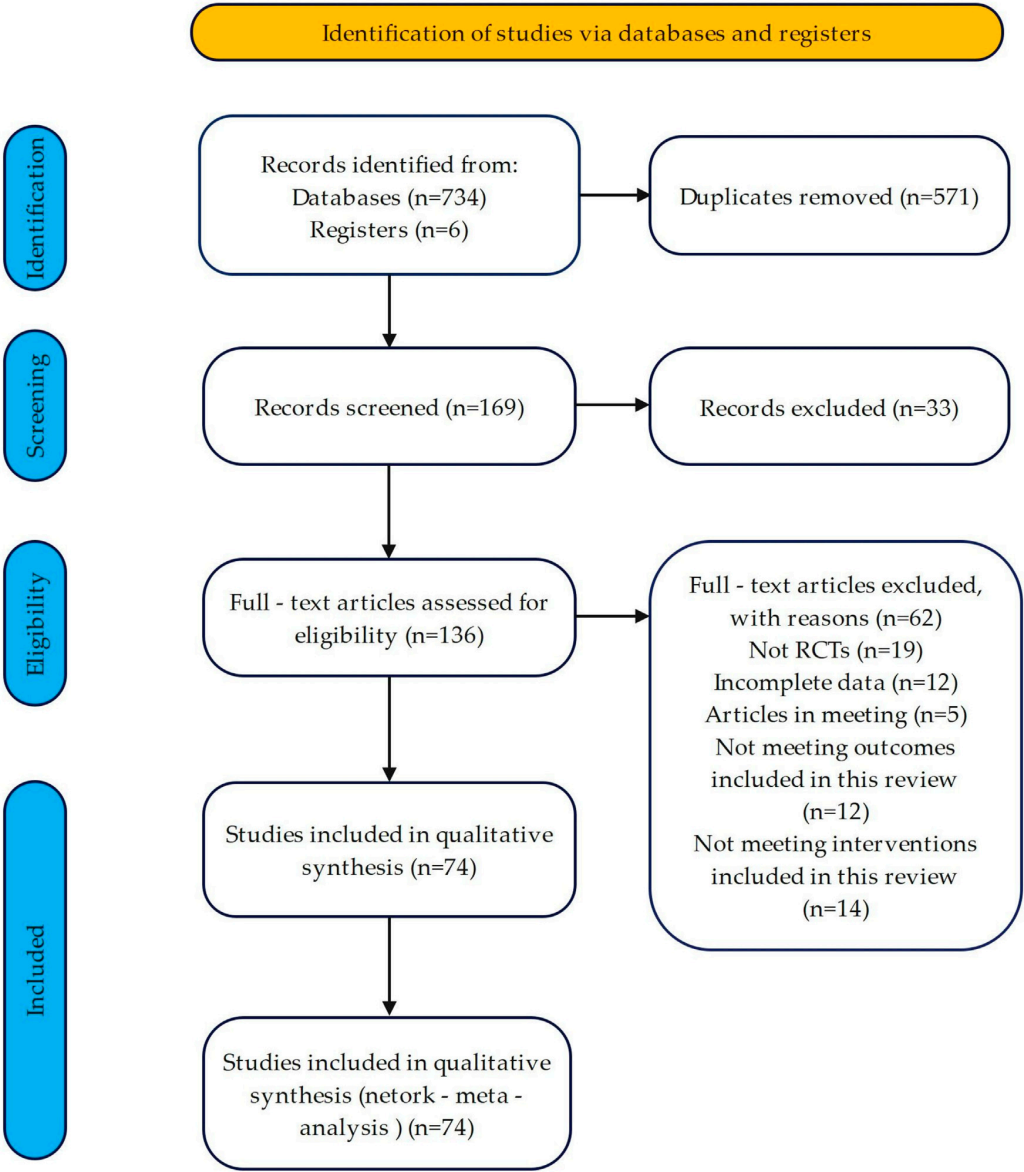
Flow diagram of literature selection. Note: PRISMA flow diagram showing the identification, screening, eligibility assessment, and inclusion of studies in the Network Meta-Analysis (NMA).

A total of 734 records were identified through electronic database searches, with an additional 6 records obtained via manual searching. After removing duplicates, 169 records remained for title and abstract screening, of which 33 were excluded. The remaining 136 full-text articles were assessed for eligibility, and 62 were excluded for the following reasons: non-randomized trials, incomplete data, conference abstracts, and interventions not meeting the inclusion criteria. A total of 74 studies were ultimately included in this analysis (Figure 1).

### 3.2 Quality assessment of the included studies

**FIGURE 2:**
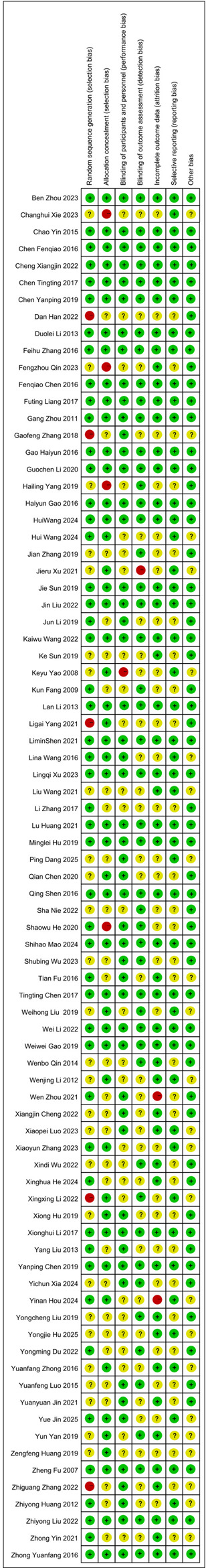
Risk of bias summary. Note: This figure was generated based on the Cochrane Collaboration RoB 2.0 tool evaluation and is only included in randomized controlled trials. “+” indicates low risk of bias, “−” indicates high risk of bias, and “?” indicates insufficient information to make a judgment. The evaluation was independently completed by two researchers and agreed upon through third-party arbitration, without blind weight adjustment of the outcome.

Of the included studies, 33 were judged to be at low risk of bias, 28 at moderate risk, and 13 at high risk. Most studies reported using randomization; however, several did not provide sufficient details regarding sequence generation or allocation concealment. Blinding of participants and investigators was reported in most studies. However, for subjective outcomes such as TCM syndrome scores, blinding at the outcome assessment stage was rarely clarified, raising concerns about potential detection bias. Detailed assessments are provided in Figure 2.

### 3.3 Characteristics of the included studies

In total, 74 RCTs involving 5,898 patients diagnosed with sepsis and gastrointestinal dysfunction were included in the analysis. Interventions in the experimental groups included treatment with one of the following TCM formulas or preparations: Banxia Xiexin Tang (Wenjing, 2012; Zhang et al., 2016; Lina et al., 2016; Gaofeng and Zhaoqiu, 2018; Hailing et al., 2019; Li, 2019; Guochen, 2020; Shaowu et al., 2020; Fengzhou et al., 2023), Intestinal Comfort (Chen et al., 2016; Haiyun, 2016; Tingting, 2017), Dachaihu Tang (Zhong et al., 2016), Dachengqi Tang (Yanping et al., 2019; Xiangjin et al., 2022), Rhubarb (Kun et al., 2009; Gang et al., 2011; Luo et al., 2013; Chao and Hong, 2015; Tian, 2016; Li, 2017; Jie et al., 2019; Minglei et al., 2019; Yun, 2019; Ligai et al., 2021; Lu and Qiaoyan, 2021; Zhong and Ligai, 2021; Zhou et al., 2021; Shubing et al., 2023), Dahuang Fuzi Tang (Yufeng, 2015; Author Anonymous, 2017; Xionghui and Dongshan, 2017; Jun, 2019; Xiong and Xiaoming, 2019; Yongcheng et al., 2019; Jieru and Zhichao, 2021; Dan et al., 2022; Jin et al., 2022; Kaiwu, 2022; Zhiyong et al., 2022; Xiaopei, 2023), Dahuang Gancao Tang (Zheng, 2007; Keyu, 2008; Qin and Huang, 2014), Dahuang Mudan Tang (Xie et al., 2023; Xiaoyun, 2023; Ping and Hua, 2025; Yongjie and Yongheng, 2025), Tiaoqi Tongfu Tang (Zhiyong, 2012; Leland et al., 2013; Ke et al., 2019; Xingxing, 2022; Zhang et al., 2022; Hui and Qingyu, 2024), Houpo Heji (Limin et al., 2021; Wei et al., 2022; Shihao et al., 2024; Xinghua et al., 2024), Sijunzi Tang (Yang, 2013; Niesha, 2022; Lingqi and Jiaping, 2023), Liangge San (Qing, 2016; Yue and Rong, 2025), Xuebijing (Jian et al., 2019; Ben, 2023), Yantiao Fang (Qian, 2020; Jinyuanyuan et al., 2021; Liu, 2021), Yiqi Tang (Zengfeng and Rukang, 2019; Zhiguang, 2022; Yinan, 2024), Zengye Chengqi Tang (Weiwei et al., 2019; Yongming et al., 2022; Spring et al., 2024), and conventional therapy. Of the included studies, 50 reported gastrointestinal dysfunction scores, 24 reported TCM syndrome scores, 57 reported APACHE II scores, 11 reported AGI grades, 18 reported D-lactate levels, 14 reported DAO levels, 21 reported TNF-α, 16 reported IL-6, and 26 reported IAP. The characteristics of the included studies are summarized in Supplementary Table S4.

### 3.4 Network meta-analysis

The complete network plots of the included interventions are presented in Supplementary Figures 3, 5, 7, 9, 11, 13, 15, 17, 19. Figures 3–11 present the comparative effect estimates across all interventions. The complete network plots of the included interventions are presented in Figures 12–20.

**FIGURE 3:**
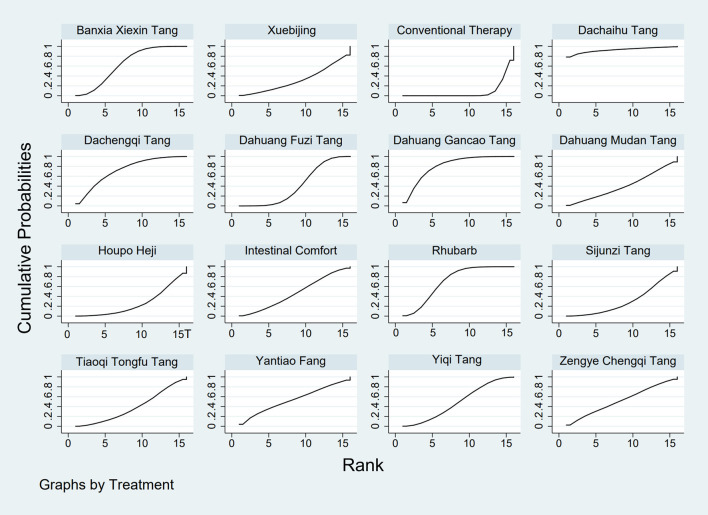
SUCRA plot for GID score. Note: Surface Under the Cumulative Ranking curve plot for GID score. Higher SUCRA values indicate a greater probability of a TCM intervention being the most effective. SUCRA: Surface Under the Cumulative Ranking curve; GID: Gastrointestinal Dysfunction; TCM: Traditional Chinese Medicine.

**FIGURE 4:**
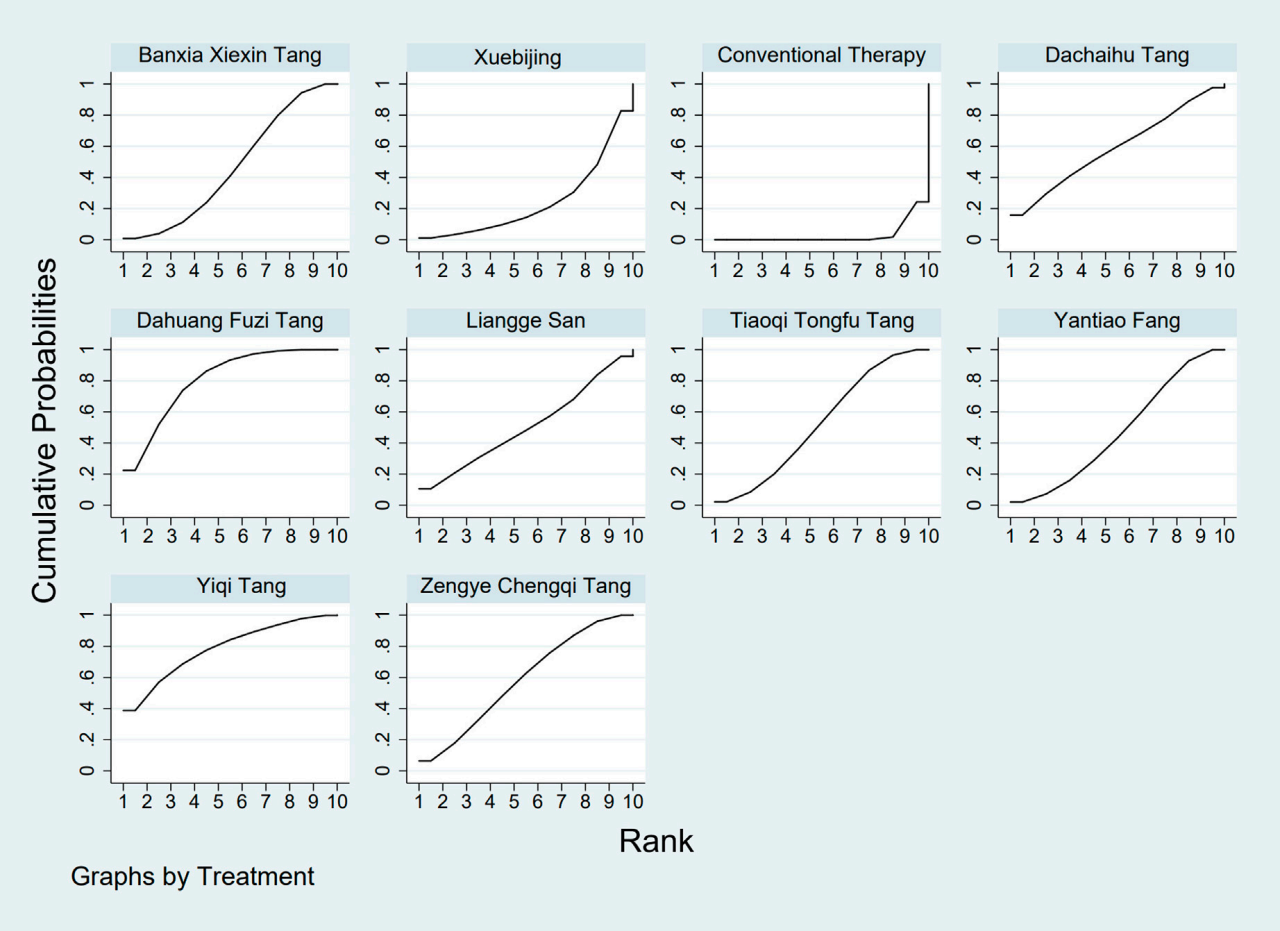
SUCRA plot for TCM symptom score. Note: Surface Under the Cumulative Ranking curve plot for Traditional Chinese Medicine symptom score. Higher SUCRA values indicate higher probability of being the most effective. SUCRA: Surface Under the Cumulative Ranking curve; TCM: Traditional Chinese Medicine.

**FIGURE 5:**
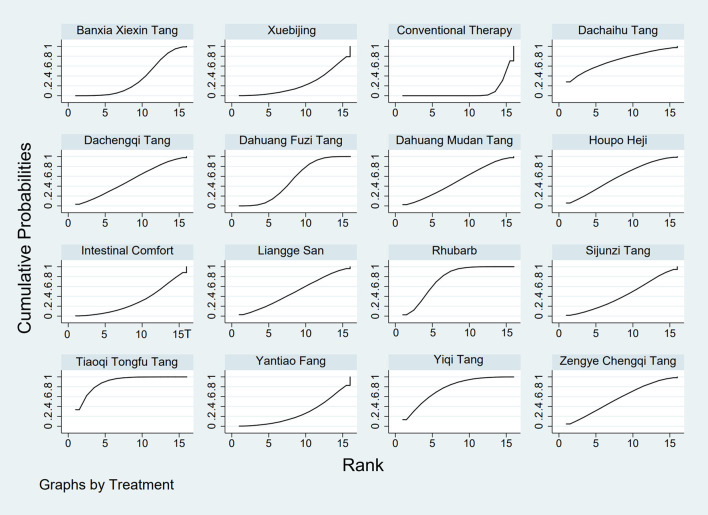
SUCRA plot for APACHE II score. Note: Surface Under the Cumulative Ranking curve plot for Acute Physiology and Chronic Health Evaluation II score. Higher SUCRA values indicate higher probability of treatment being most effective. SUCRA: Surface Under the Cumulative Ranking curve; APACHE II: Acute Physiology and Chronic Health Evaluation II.

**FIGURE 6:**
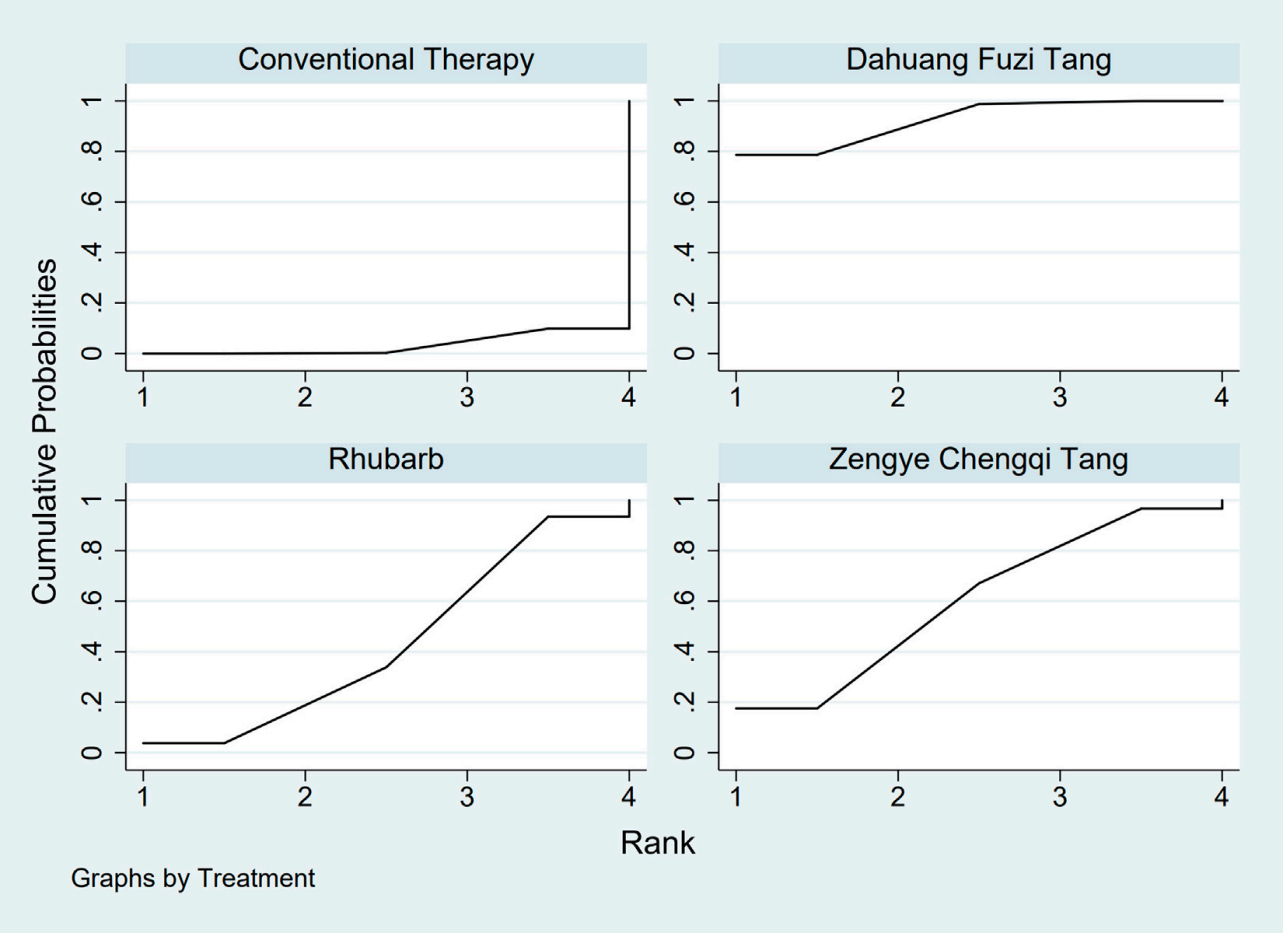
SUCRA plot for AGI classification. Note: Surface Under the Cumulative Ranking curve plot for Acute Gastrointestinal Injury classification. Higher SUCRA values indicate higher probability of being the best treatment. SUCRA: Surface Under the Cumulative Ranking curve; AGI: Acute Gastrointestinal Injury.

**FIGURE 7:**
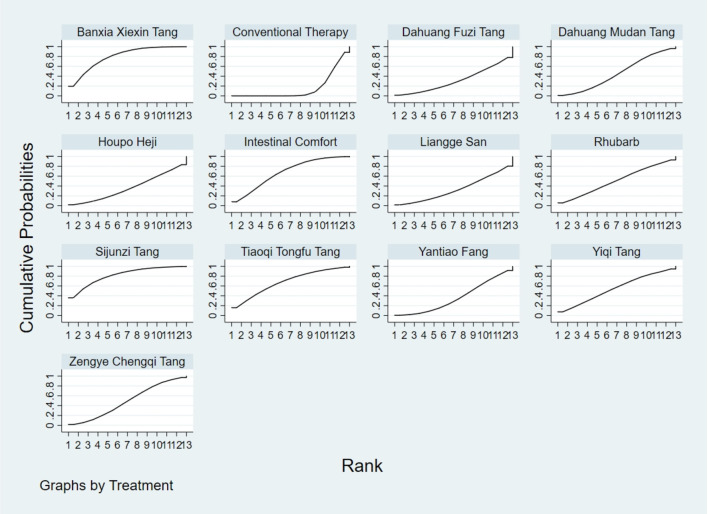
SUCRA plot for D-lactic acid. Note: Surface Under the Cumulative Ranking curve plot for D-lactic acid levels. Higher SUCRA values indicate higher probability of effective reduction. SUCRA: Surface Under the Cumulative Ranking curve.

**FIGURE 8:**
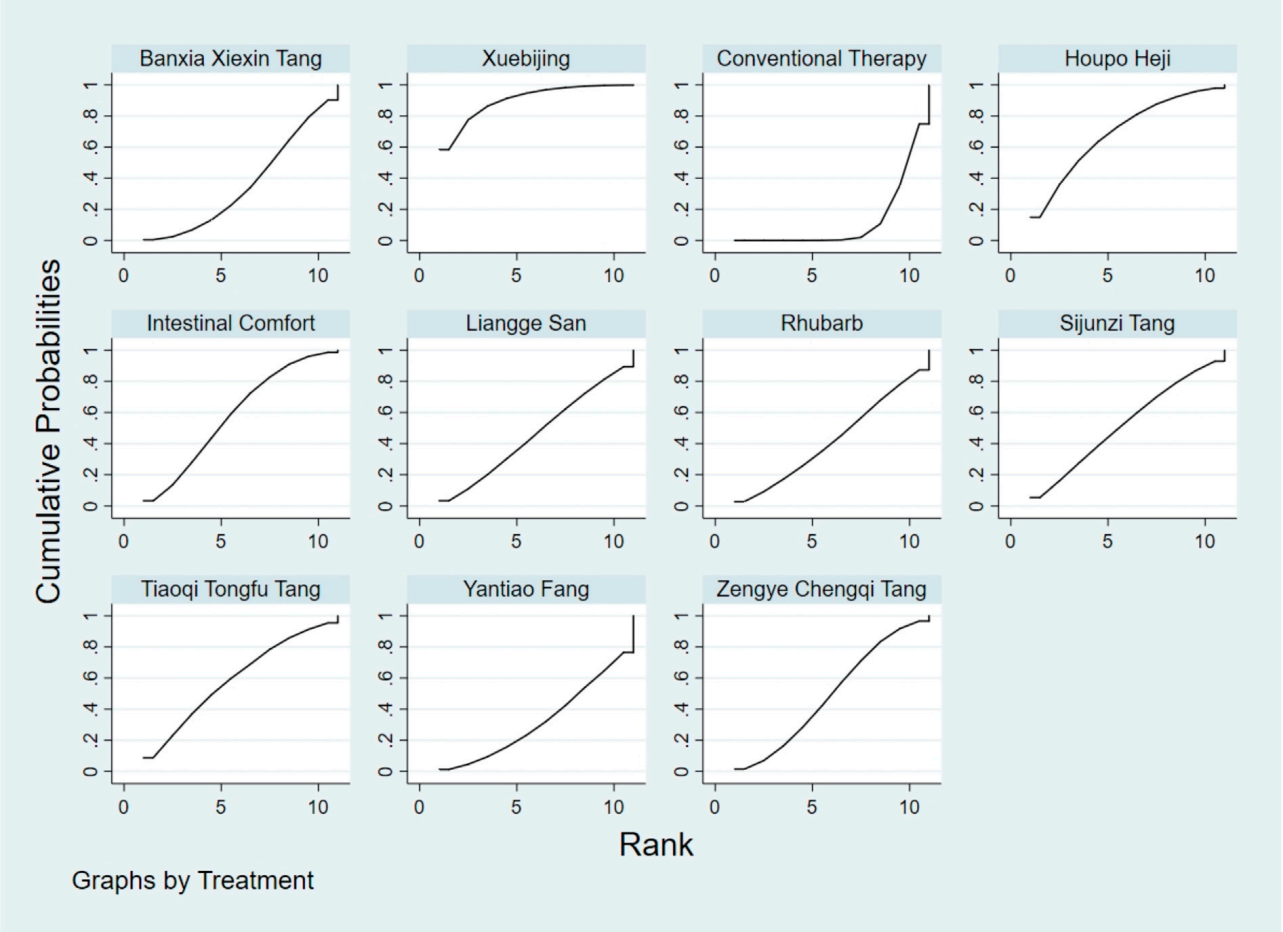
SUCRA plot for DAO mean. Note: Surface Under the Cumulative Ranking curve plot for Diamine Oxidase mean. Higher SUCRA values indicate higher probability of being most effective. SUCRA: Surface Under the Cumulative Ranking curve; DAO: Diamine Oxidase.

**FIGURE 9:**
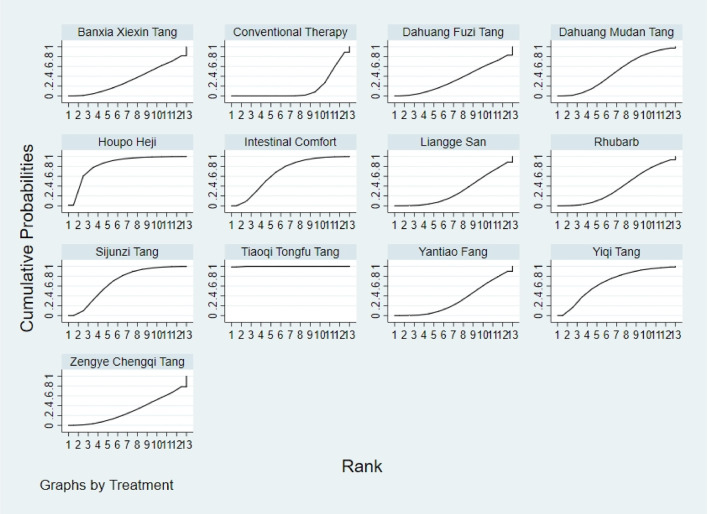
SUCRA plot for TNF-α. Note: Surface Under the Cumulative Ranking curve plot for Tumor Necrosis Factor-α levels. Higher SUCRA values indicate greater probability of effective reduction. SUCRA: Surface Under the Cumulative Ranking curve; TNF-α: Tumor Necrosis Factor-α.

**FIGURE 10:**
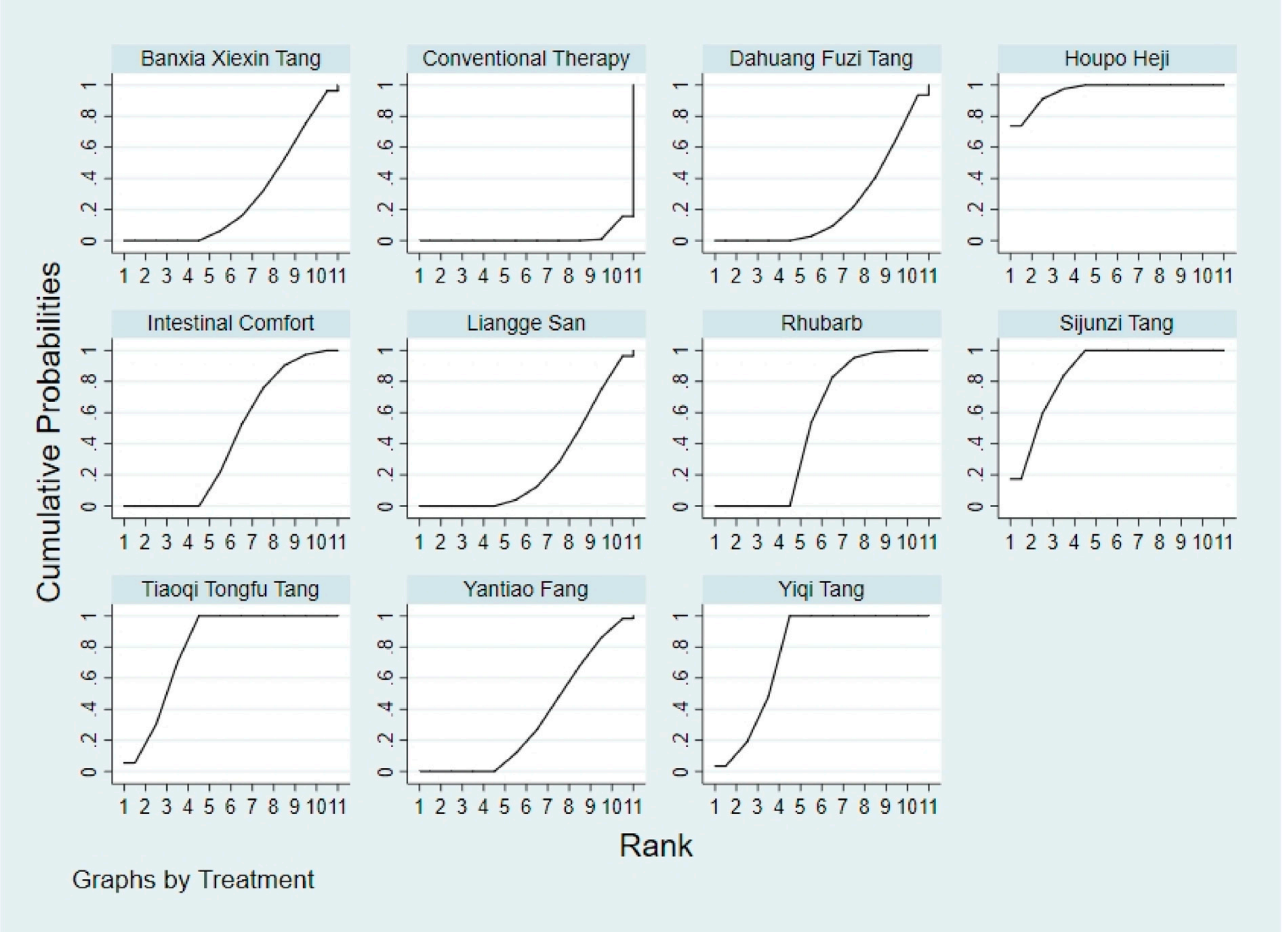
SUCRA plot for IL-6. Note: Surface Under the Cumulative Ranking curve plot for Interleukin-6 levels. Higher SUCRA values indicate higher probability of effective reduction. SUCRA: Surface Under the Cumulative Ranking curve; IL-6: Interleukin-6.

**FIGURE 11:**
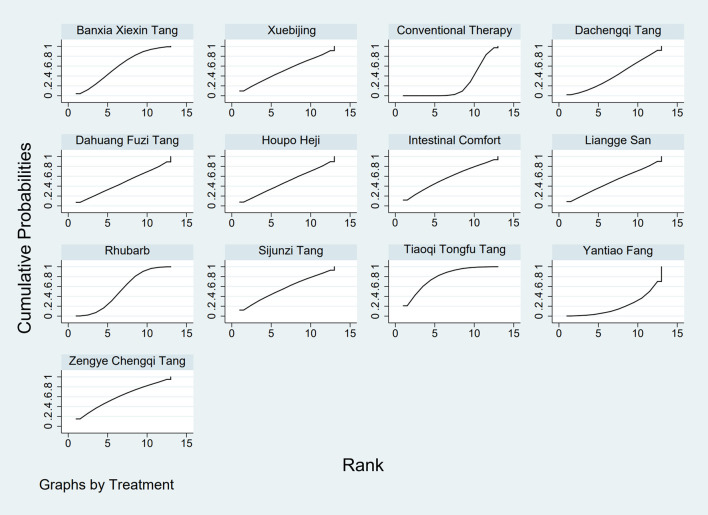
SUCRA plot for IAP. Note: Surface Under the Cumulative Ranking curve plot for IAP. Higher SUCRA values indicate higher probability of effective reduction. SUCRA: Surface Under the Cumulative Ranking curve; IAP: Intra-abdominal Pressure.

**FIGURE 12:**
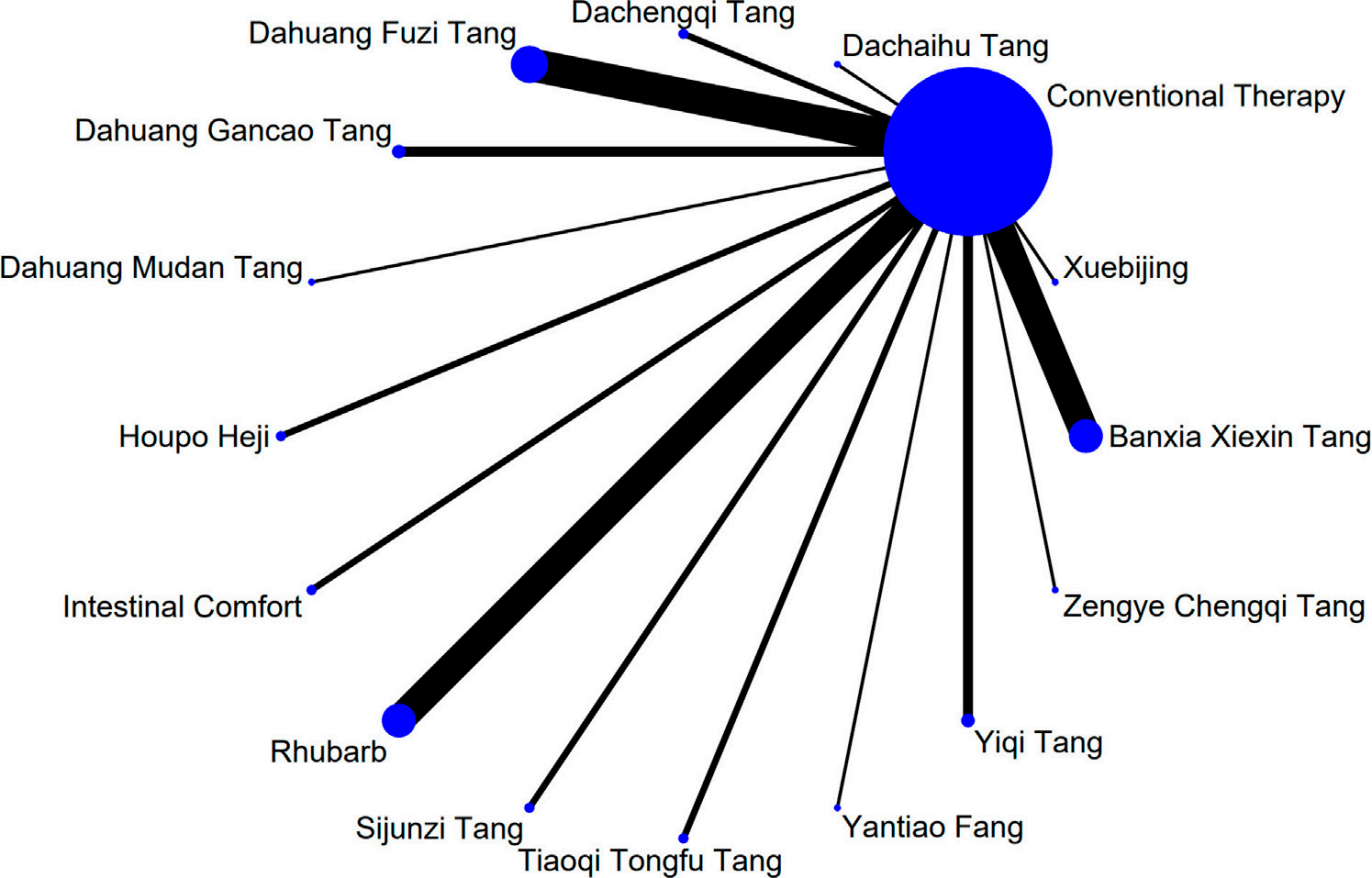
NMA figure for Gastrointestinal Dysfunction (GID) score. Note: Network Meta-Analysis diagram for Gastrointestinal Dysfunction score. Nodes represent Traditional Chinese Medicine (TCM) interventions; lines indicate direct head-to-head comparisons. NMA: Network Meta-Analysis; GID: Gastrointestinal Dysfunction; TCM: Traditional Chinese Medicine.

**FIGURE 13:**
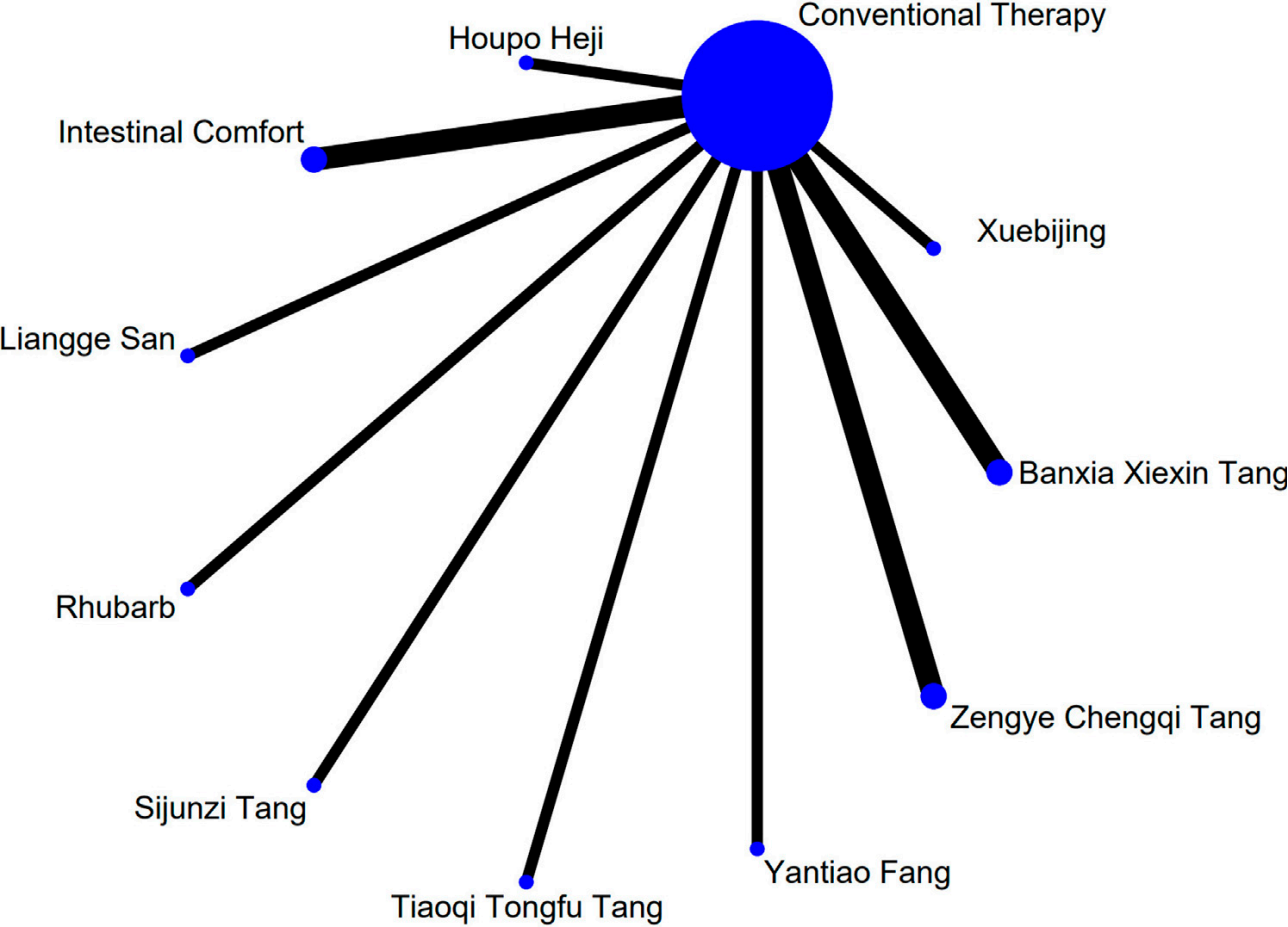
NMA figure for DAO mean. Note: Network Meta-Analysis diagram for Diamine Oxidase mean. Nodes represent interventions; lines indicate direct comparisons. NMA: Network Meta-Analysis; DAO: Diamine Oxidase.

**FIGURE 14:**
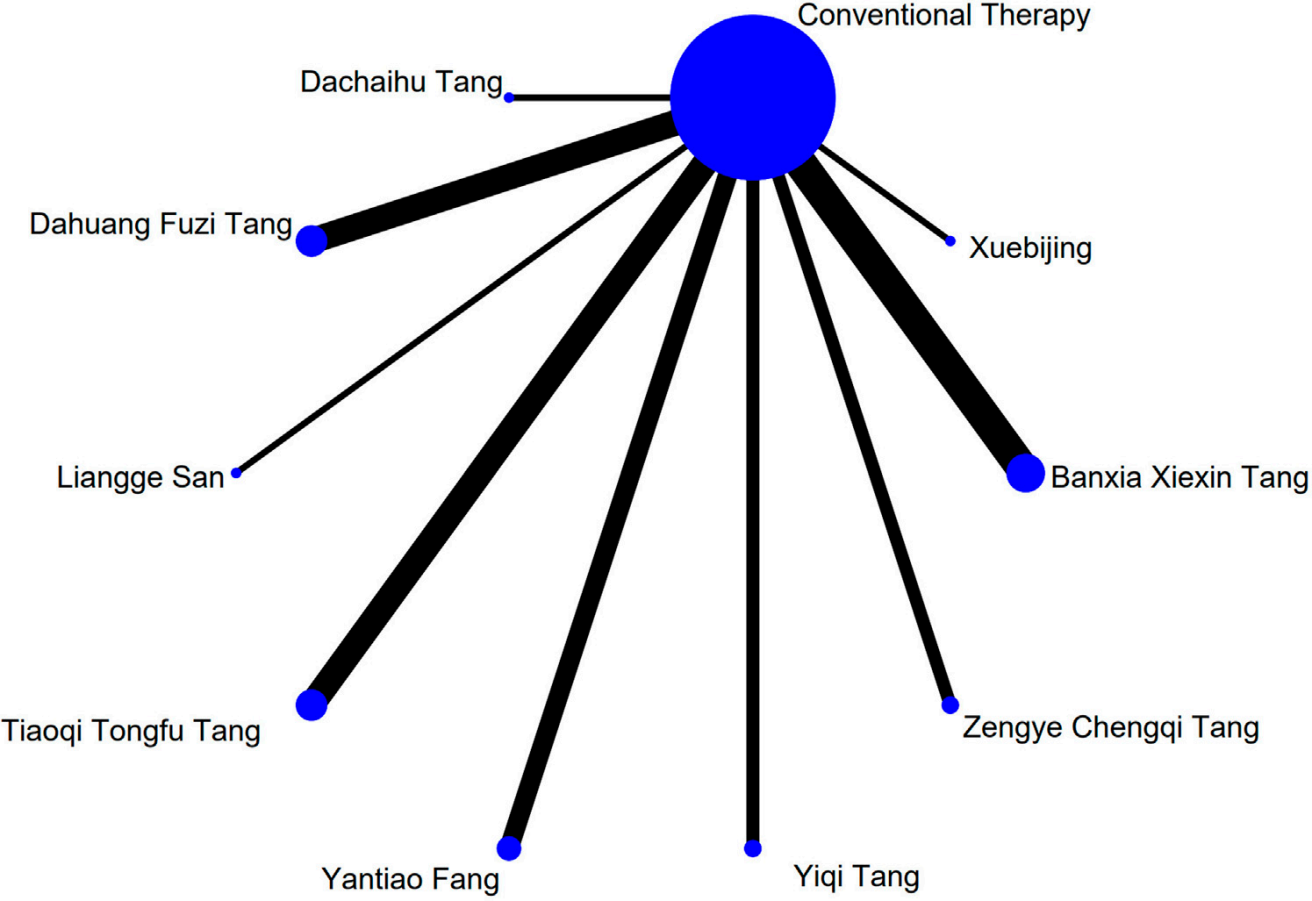
NMA figure for TCM symptom score. Note: Network Meta-Analysis diagram for Traditional Chinese Medicine symptom score. Nodes represent interventions; lines indicate direct comparisons. NMA: Network Meta-Analysis; TCM: Traditional Chinese Medicine.

**FIGURE 15:**
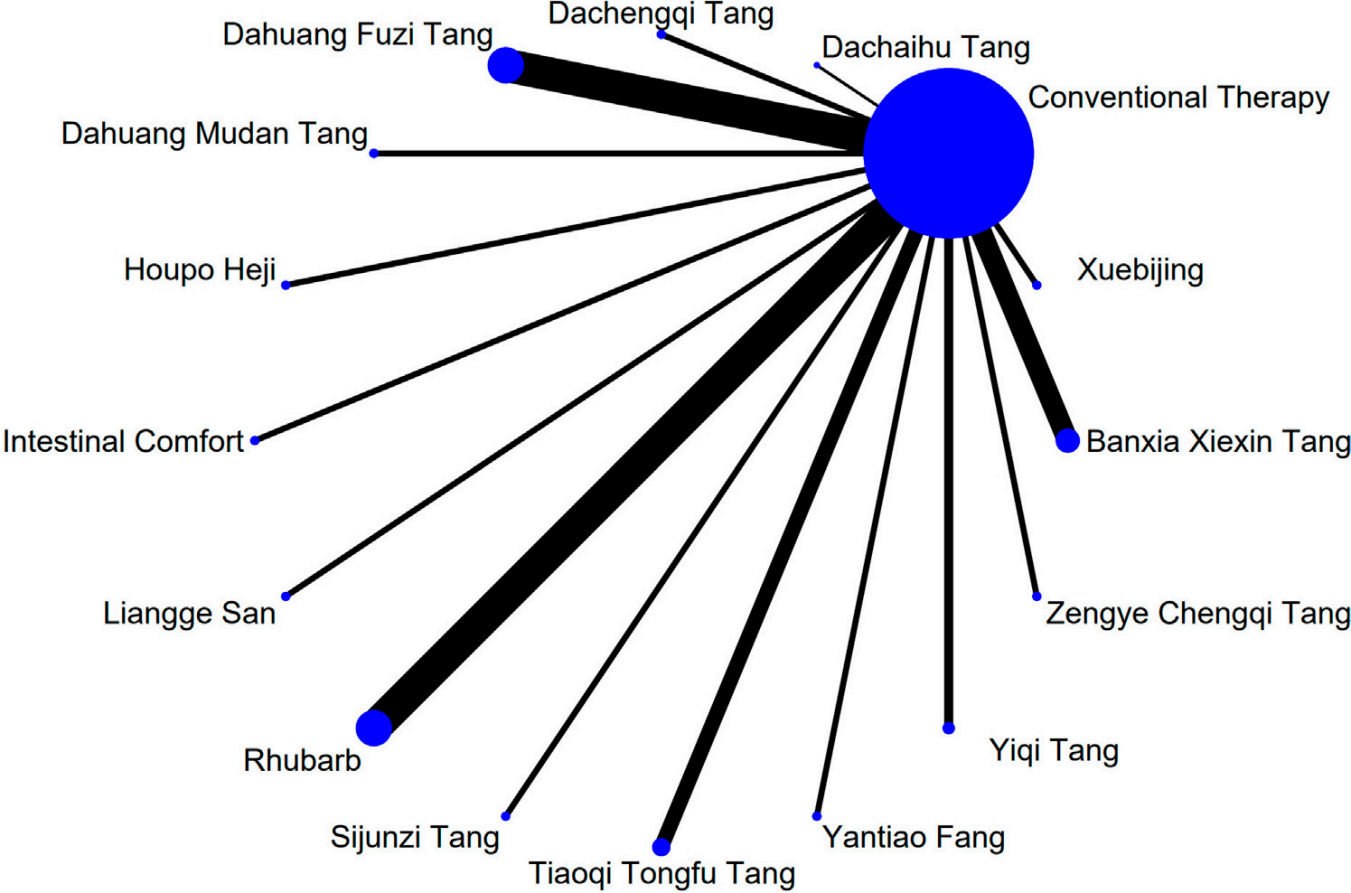
NMA figure for APACHE II score. Note: Network Meta-Analysis diagram for Acute Physiology and Chronic Health Evaluation II score. Nodes represent interventions; lines indicate direct comparisons. NMA: Network Meta-Analysis; APACHE II: Acute Physiology and Chronic Health Evaluation II.

**FIGURE 16:**
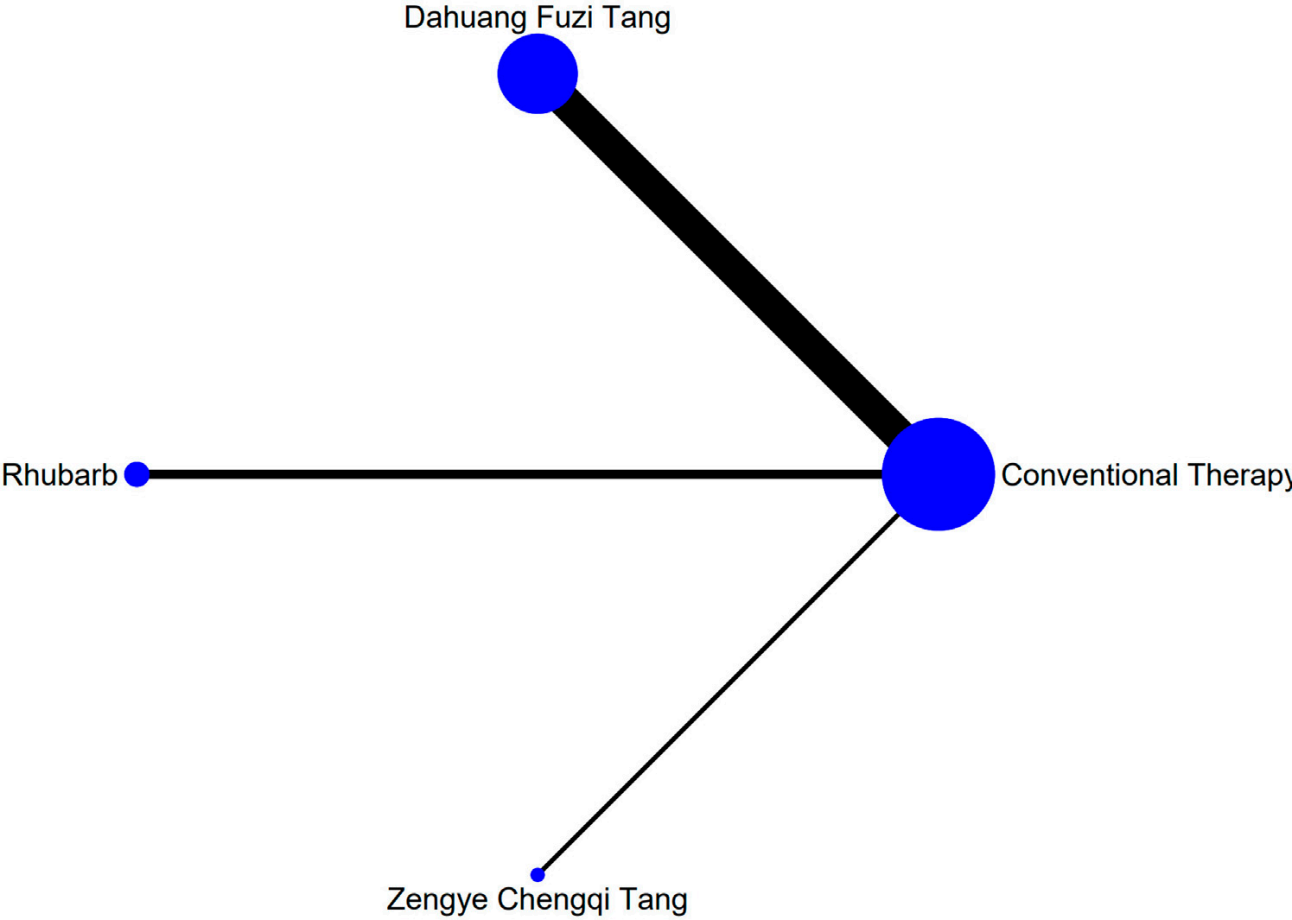
NMA figure for AGI classification. Note: Network Meta-Analysis diagram for Acute Gastrointestinal Injury classification. Nodes represent interventions; lines indicate direct comparisons. NMA: Network Meta-Analysis; AGI: Acute Gastrointestinal Injury.

**FIGURE 17:**
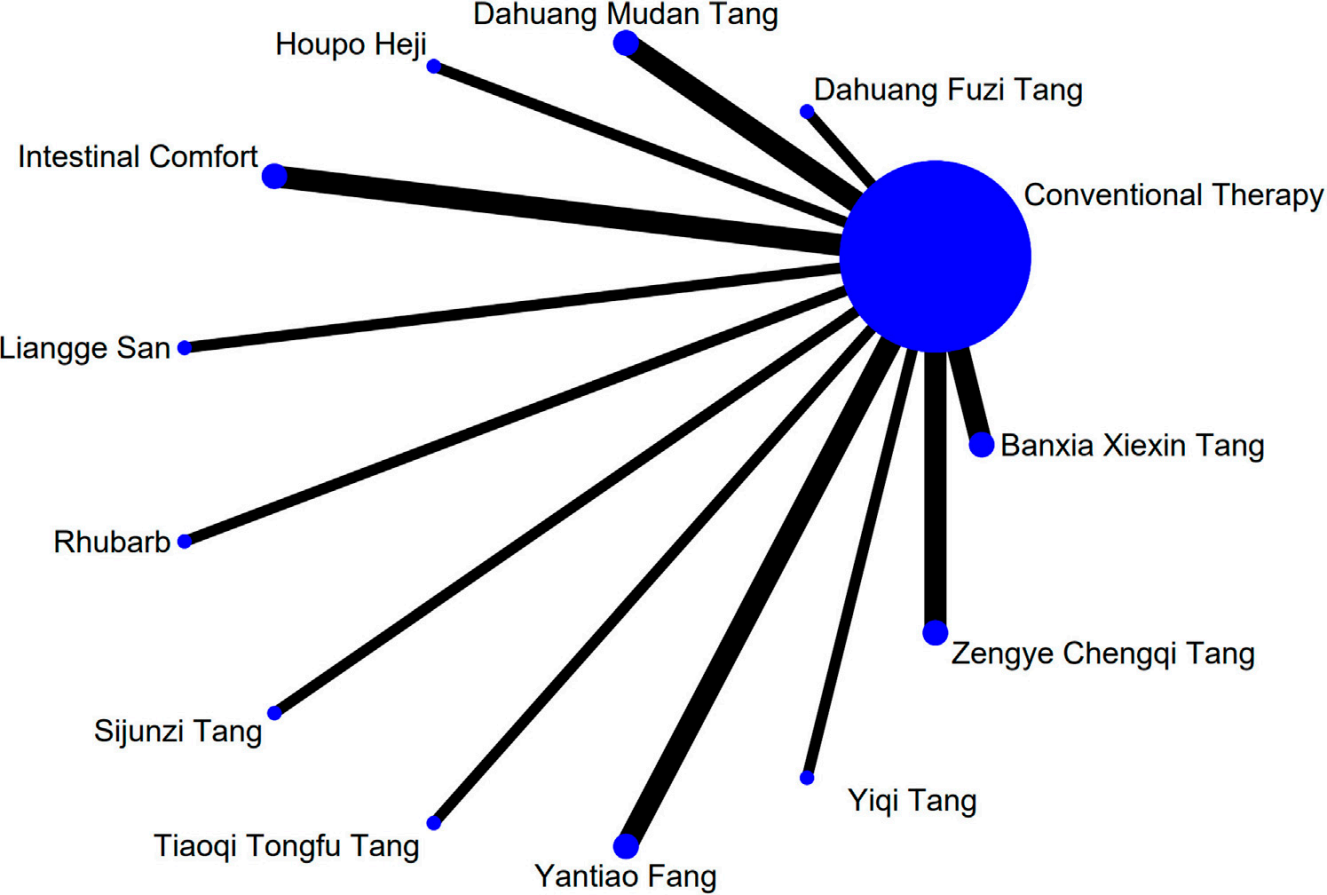
NMA figure for D-lactic acid. Note: Network Meta-Analysis diagram for D-lactic acid levels. Nodes represent interventions; lines indicate direct comparisons. NMA: Network Meta-Analysis.

**FIGURE 18:**
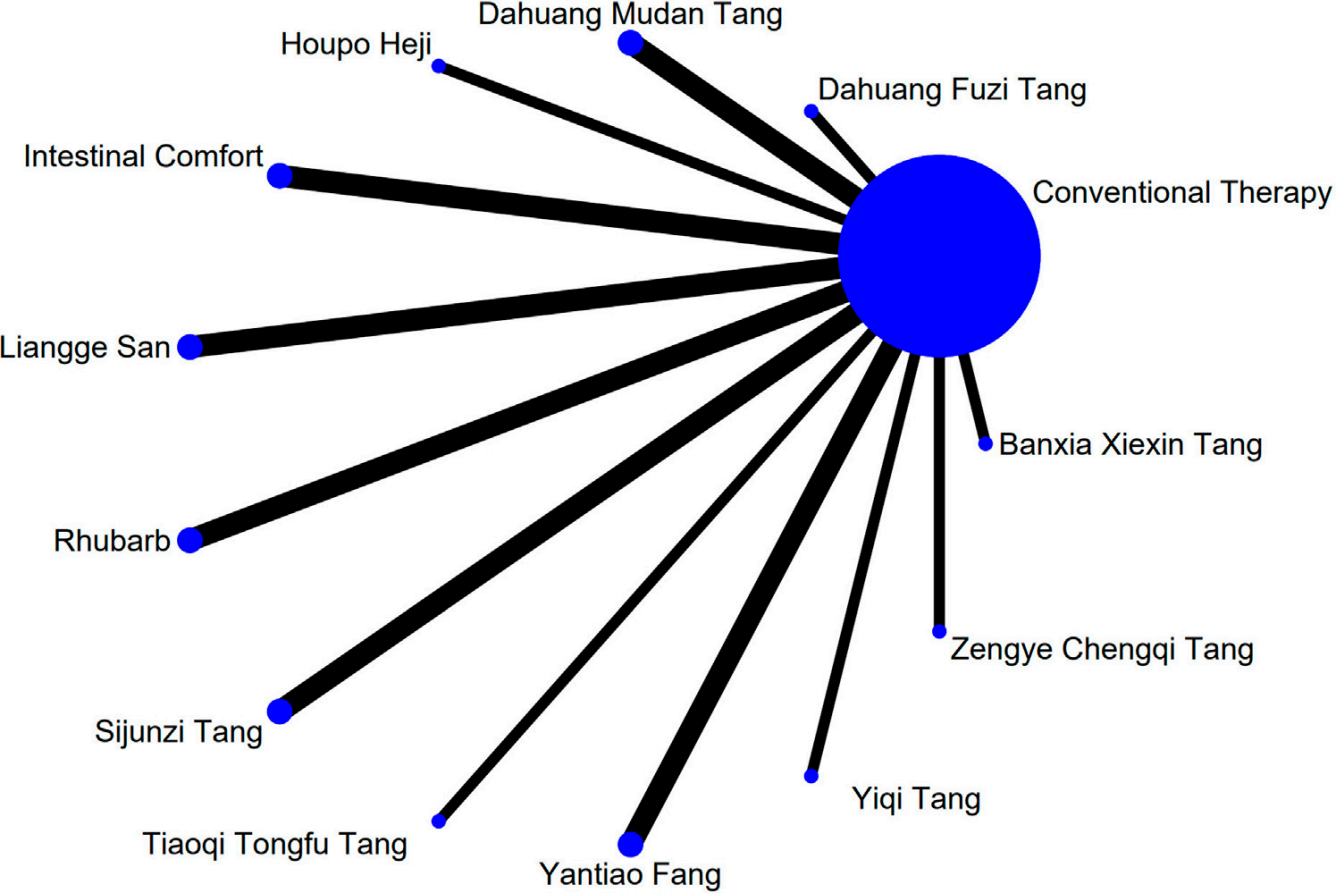
NMA figure for TNF-α. Note: Network Meta-Analysis diagram for Tumor Necrosis Factor-α. Nodes represent interventions; lines indicate direct comparisons. NMA: Network Meta-Analysis; TNF-α: Tumor Necrosis Factor-α.

**FIGURE 19:**
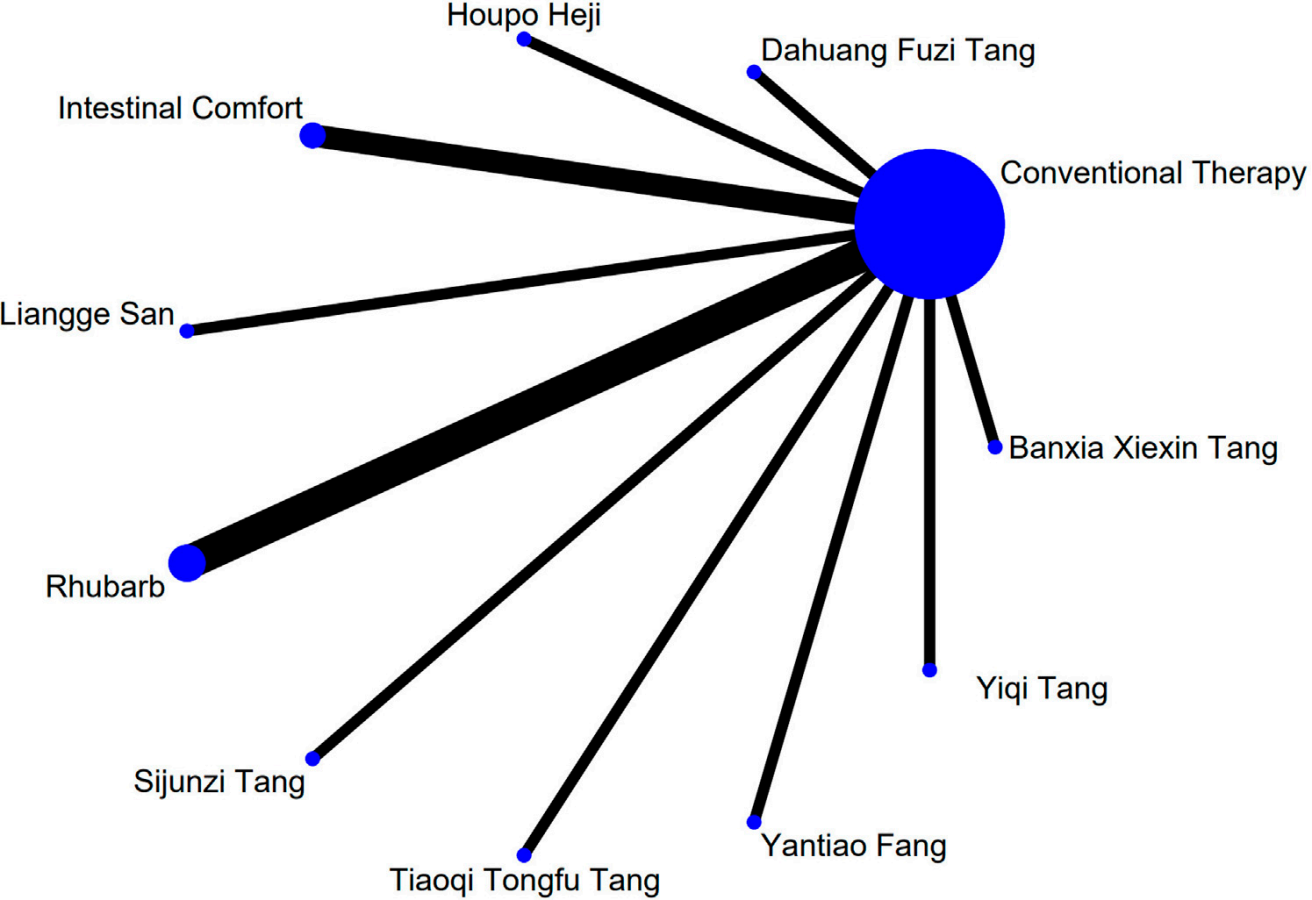
NMA figure for IL-6. Note: Network Meta-Analysis diagram for Interleukin-6. Nodes represent interventions; lines indicate direct comparisons. NMA: Network Meta-Analysis; IL-6: Interleukin-6.

**FIGURE 20:**
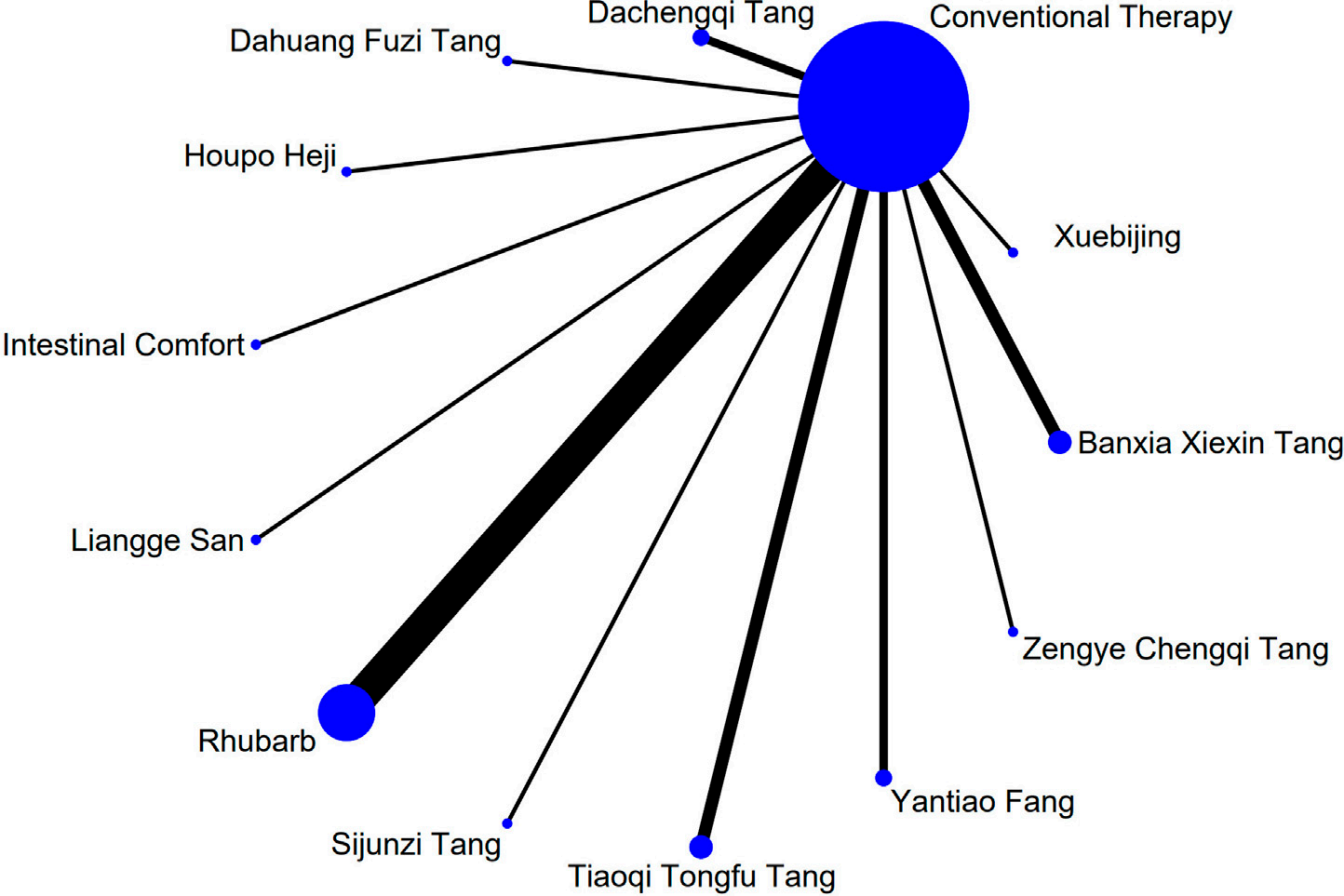
NMA figure for IAP. Note: Network Meta-Analysis diagram for Intra-abdominal Pressure. Nodes represent interventions; lines indicate direct comparisons. NMA: Network Meta-Analysis; IAP: Intra-abdominal Pressure.

#### 3.4.1 Gastrointestinal dysfunction score

All included comparisons passed the consistency test between direct and indirect evidence (all P-values >0.05), indicating acceptable consistency within the evidence network. Detailed results are presented in Supplementary Table S5A.

The results showed that, compared with conventional treatment in the control group, the following interventions significantly reduced gastrointestinal dysfunction scores: Banxia Xiexin Tang [MD = −0.76, 95% CI: −1.09 to −0.44], Dachaihu Tang [MD = −2.03, 95% CI: −3.86 to −0.20], Dachengqi Tang [MD = −0.91, 95% CI: −1.54 to −0.29], Rhubarb [MD = −0.84, 95% CI: −1.13 to −0.54], Dahuang Fuzi Tang [MD = −0.50, 95% CI: −0.78 to −0.22], Dahuang Gancao Tang [MD = −1.03, 95% CI: −1.55 to −0.51], and Yiqi Tang [MD = −0.58, 95% CI: −1.11 to −0.05]. Detailed results are presented in Supplementary Table S6A. Among these, Dachaihu Tang ranked highest in probability of effectiveness (92.7%), as shown in Figure 3, with the detailed cumulative ranking probabilities summarized in Supplementary Table S7A. Figure 12 presents the comparative effect estimates across all interventions.

#### 3.4.2 TCM symptom score

All included comparisons passed the consistency test between direct and indirect evidence (all P-values >0.05), indicating acceptable consistency within the evidence network. Detailed results are provided in Supplementary Table S5B.

The results showed that, compared with conventional treatment in the control group, the following interventions were significantly more effective in reducing TCM symptom scores: Banxia Xiexin Tang [MD = −3.48, 95% CI: −5.41 to −1.54], Dahuang Fuzi Tang [MD = −5.31, 95% CI: −7.45 to −3.17], Tiaoqi Tongfu Tang [MD = −3.71, 95% CI: −5.98 to −1.43], Yantiao Fang [MD = −3.47, 95% CI: −5.85 to −1.09], Yiqi Tang [MD = −5.70, 95% CI: −9.84 to −1.56], and Zengye Chengqi Tang [MD = −4.04, 95% CI: −6.73 to −1.35]. Detailed results are presented in Supplementary Table S6B. Among these, Yiqi Tang ranked highest in probability of effectiveness (78.8%), as shown in Figure 4, with the detailed cumulative ranking probabilities summarized in Supplementary Table S7B. Figure 14 presents the comparative effect estimates across all interventions.

#### 3.4.3 Apache Ⅱ score

All included comparisons passed the consistency test between direct and indirect evidence (all P-values >0.05), indicating acceptable consistency within the evidence network. Detailed results are provided in Supplementary Table S5C.

The results showed that, compared with conventional treatment in the control group, the following interventions were significantly more effective in reducing APACHE II scores: Rhubarb [MD = −5.26, 95% CI: −7.09 to −3.43], Dahuang Fuzi Tang [MD = −3.66, 95% CI: −5.47 to −1.84], Tiaoqi Tongfu Tang [MD = −6.90, 95% CI: −9.66 to −4.13], and Yiqi Tang [MD = −5.64, 95% CI: −9.12 to −2.16]. Detailed results are presented in Supplementary Table S6C. Tiaoqi Tongfu Tang ranked highest in probability of effectiveness for reducing APACHE II scores (89.7%), as shown in Figure 5, with the detailed cumulative ranking probabilities summarized in Supplementary Table S7C. Figure 15 presents the comparative effect estimates across all interventions.

#### 3.4.4 AGI classification

All included comparisons passed the consistency assessment between direct and indirect evidence (all P-values >0.05), indicating acceptable consistency within the evidence network. There is no inconsistency in the data.

The results showed that, compared with conventional treatment in the control group, the following interventions were significantly more effective in improving AGI classification: Rhubarb [MD = 0.56, 95% CI: 0.26 to 1.19], Dahuang Fuzi Tang [MD = 0.27, 95% CI: 0.19 to 0.40], and Zengye Chengqi Tang [MD = 0.44, 95% CI: 0.18 to 1.08]. Detailed results are presented in Supplementary Table S6D. Dahuang Fuzi Tang ranked highest in probability of effectiveness for improving AGI classification (92.5%), as shown in Figure 6, with the detailed cumulative ranking probabilities summarized in Supplementary Table S7D. Figure 16 presents the comparative effect estimates across all interventions.

#### 3.4.5 D-lactic acid

All included comparisons passed the consistency assessment between direct and indirect evidence (all P-values >0.05), indicating acceptable consistency within the evidence network. Detailed results are provided in Supplementary Table S5D.

The results showed that, compared with conventional treatment in the control group, the following interventions were significantly more effective in reducing D-lactic acid levels: Sijunzi Tang [MD = −3.51, 95% CI: −6.73 to −0.29], Banxia Xiexin Tang [MD = −3.14, 95% CI: −5.40 to −0.88], Tiaoqi Tongfu Tang [MD = −2.59, 95% CI: −5.75 to 0.57], and Intestinal Comfort [MD = −2.43, 95% CI: −4.71 to −0.15]. Detailed results are presented in Supplementary Table S6E. Sijunzi Tang ranked highest in probability of effectiveness for reducing D-lactic acid levels (81.8%), as shown in Figure 7, with the detailed cumulative ranking probabilities summarized in Supplementary Table S7E. Figure 17 presents the comparative effect estimates across all interventions.

#### 3.4.6 DAO mean

All included comparisons passed the consistency assessment between direct and indirect evidence (all P-values >0.05), indicating acceptable consistency within the evidence network. Detailed results are provided in Supplementary Table S5E.

The results showed that, compared with conventional treatment in the control group, the following interventions were significantly more effective in reducing mean DAO levels: Xuebijing [MD = −4.57, 95% CI: −7.86 to −1.28], Houpo Heji [MD = −2.85, 95% CI: −6.06 to 0.37], Tiaoqi Tongfu Tang [MD = −2.30, 95% CI: −5.46 to 0.86], and Intestinal Comfort [MD = -2.15, 95% CI:-4.42 to 0.12]. Detailed results are presented in Supplementary Table S6F. Xuebijing ranked highest in probability of effectiveness for reducing DAO levels (90.2%), as shown in Figure 8, with the detailed cumulative ranking probabilities summarized in Supplementary Table S7F. Figure 13 presents the comparative effect estimates across all interventions.

#### 3.4.7 TNF-α

All included comparisons passed the consistency assessment between direct and indirect evidence (all P-values >0.05), indicating acceptable consistency within the evidence network. Detailed results are provided in Supplementary Table S5F.

The results showed that, compared with conventional treatment in the control group, the following interventions were significantly more effective in reducing TNF-α levels: Tiaoqi Tongfu Tang [MD = −7.65, 95% CI: −10.27 to −5.04], Houpo Heji [MD = −3.46, 95% CI: −5.94 to −0.97], Sijunzi Tang [MD = −2.13, 95% CI: −3.83 to −0.42], and Intestinal Comfort [MD = −2.09, 95% CI: −3.82 to −0.37]. Detailed results are presented in Supplementary Table S6G. Tiaoqi Tongfu Tang ranked highest in probability of effectiveness for reducing TNF-α levels (99.9%), as shown in Figure 9, with the detailed cumulative ranking probabilities summarized in Supplementary Table S7G. Figure 18 presents the comparative effect estimates across all interventions.

#### 3.4.8 IL-6

All included comparisons passed the consistency assessment between direct and indirect evidence (all P-values >0.05), indicating acceptable consistency within the evidence network. Detailed results are provided in Supplementary Table S5G.

Network meta-analysis results showed that, compared with conventional treatment in the control group, the following interventions were significantly more effective in reducing IL-6 levels: Houpo Heji [MD = −4.24, 95% CI: −5.34 to −3.14], Sijunzi Tang [MD = −3.63, 95% CI: −4.55 to −2.70], Tiaoqi Tongfu Tang [MD = −3.37, 95% CI: −4.12 to −2.62], and Yiqi Tang [MD = −3.19, 95% CI: −4.06 to −2.32]. Detailed results are presented in Supplementary Table S6H. Houpo Heji ranked highest in probability of effectiveness for reducing IL-6 levels (96.2%), as shown in Figure 10, with the detailed cumulative ranking probabilities summarized in Supplementary Table S7H. Figure 19 presents the comparative effect estimates across all interventions.

#### 3.4.9 IAP

All included comparisons passed the consistency assessment between direct and indirect evidence (all P-values >0.05), indicating acceptable consistency within the evidence network. Detailed results are provided in Supplementary Table S5H.

The results showed that, compared with conventional treatment in the control group, Tiaoqi Tongfu Tang was significantly more effective in reducing IAP [MD = −5.88, 95% CI: −12.26 to 0.50]. Detailed results are presented in Supplementary Table S6I. Tiaoqi Tongfu Tang ranked highest in probability of effectiveness for reducing IAP (79.2%), as shown in Figure 11, with the detailed cumulative ranking probabilities summarized in Supplementary Table S7I. Figure 20 presents the comparative effect estimates across all interventions.

#### 3.4.10 Adverse reactions

Adverse events related to gastrointestinal symptoms—including abdominal distension, diarrhea, nausea and vomiting, reflux, and excessive gastric residual volume—were reported by Hu Xiong, Liu Yongcheng, Liang Futing, Huang Dan, Wang Hui, and others. Adverse events associated with multiple organ dysfunction syndrome (MODS) were documented by Li Jun, Gao Haiyun, Hu Minglei, Yao Keyu, Fu Tian, and Huang Zengfeng. Results showed that the incidence of most adverse events did not significantly differ from that of the control group. No serious adverse events were reported in any of the included studies. Detailed results are provided in Supplementary Table S8.

### 3.5 Publication bias test

**FIGURE 21:**
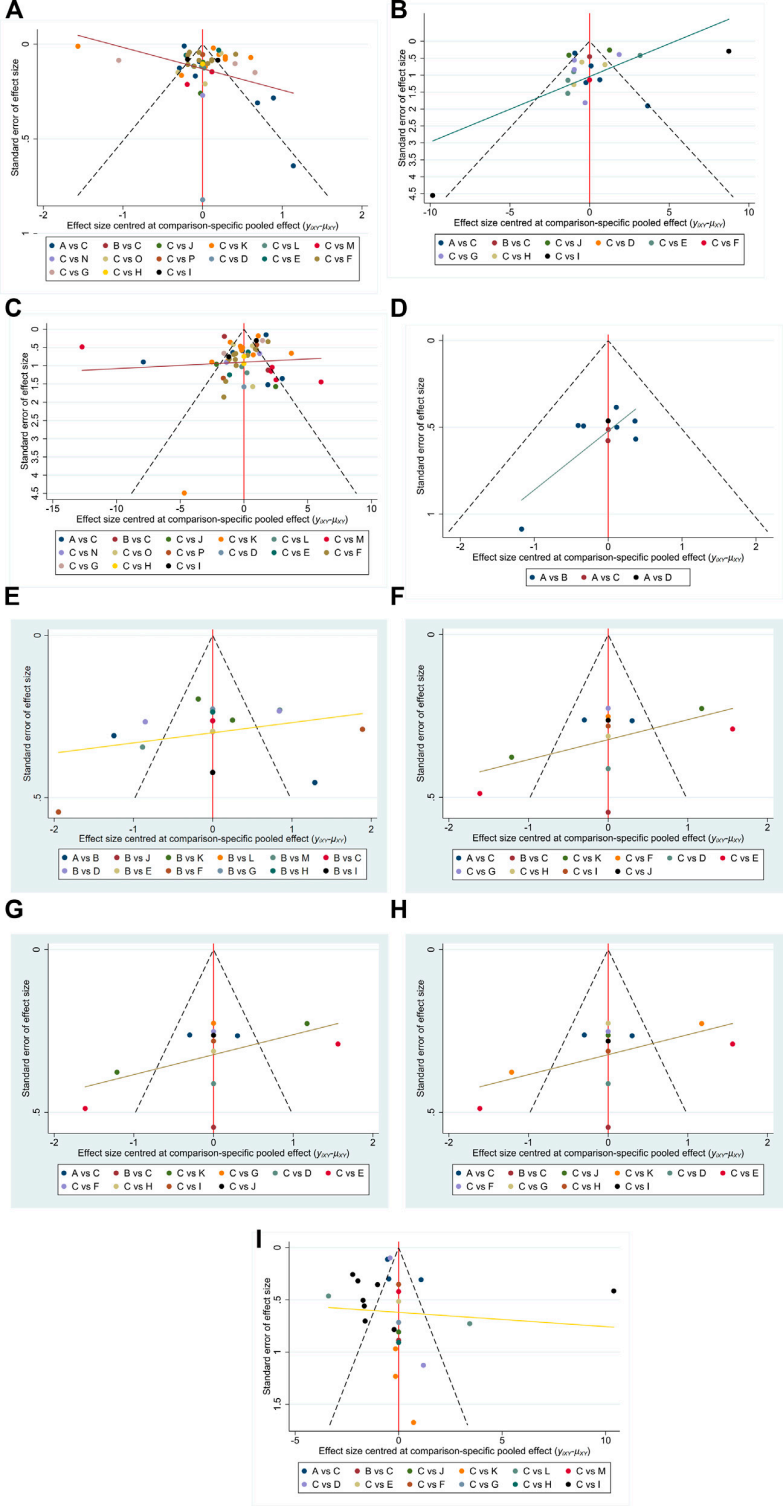
**(A–I)** Funnel plots on publication bias. Note: Funnel plots for each outcome (GID score, TCM symptom score, APACHE II, AGI, D-lactic acid, DAO mean, TNF-α, IL-6, IAP) to assess potential publication bias in the NMA. Symmetrical distribution suggests low risk of bias. NMA: Network Meta-Analysis; GID: Gastrointestinal Dysfunction; TCM: Traditional Chinese Medicine; APACHE II: Acute Physiology and Chronic Health Evaluation II; AGI: Acute Gastrointestinal Injury; DAO: Diamine Oxidase; TNF-α: Tumor Necrosis Factor-α; IL-6: Interleukin-6; IAP: Intra-abdominal Press.

Funnel plots were constructed for all outcome indicators to assess the potential presence of publication bias. Visual inspection of the funnel plots did not reveal any obvious evidence of publication bias (Wallace et al., 2009). Individual funnel plots are provided in Figure 21.

### 3.6 Heterogeneity and consistency

To further evaluate the robustness of the network, we calculated and summarized the τ^2^ estimates and network-level τ^2^/I^2^ statistics for each outcome. The results indicated that most clinical scale outcomes (e.g., AGI classification and gastrointestinal dysfunction score) exhibited relatively low heterogeneity, suggesting good overall network consistency. In contrast, some laboratory-based outcomes (such as D-lactic acid, TNF-α, and IL-6) showed higher levels of heterogeneity. This pattern is likely attributable to variations in assay methods, sampling time points, and patient-level differences rather than true inconsistency in treatment effects. Therefore, despite the presence of heterogeneity in certain biomarkers, the observed levels remain acceptable within the context of clinical research and do not undermine the overall validity of the conclusions.Detailed results are provided in Supplementary Table S9.

### 3.7 Design-by-treatment interaction

Global inconsistency across the network was evaluated using the design-by-treatment interaction model. The results indicated no significant inconsistency among comparisons. Detailed results are provided in Supplementary Table S1.

## 4 Discussion

This study conducted a network meta-analysis of 74 RCTs involving 5,898 patients to systematically evaluate the comparative efficacy of 16 TCM interventions for sepsis-induced gastrointestinal dysfunction. The results indicated that Dachaihu Tang showed the greatest improvement in gastrointestinal dysfunction scores, while Yiqi Tang was most effective in improving TCM syndrome scores. Tiaoqi Tongfu Tang demonstrated superior effects in reducing AGI grading and IAP. Regarding inflammatory response, Tiaoqi Tongfu Tang was most effective in reducing APACHE II scores and TNF-α levels, while Houpo Heji showed the strongest regulatory effect on IL-6. In terms of mucosal barrier repair, Sijunzi Tang was most effective in reducing D-lactic acid levels, and Xuebijing showed the greatest improvement in mean DAO levels.

Dachaihu Tang enhances gastrointestinal smooth muscle contractility through saikosaponin A/B and baicalin (activating cholinergic M3 receptors) and improves intestinal microcirculation via rhein (suppressing VCAM-1/ICAM-1) (Huang et al., 2023). Yiqi formulations strengthen intestinal barrier function by codonopsis polysaccharides (TLR4/MyD88-mediated epithelial proliferation) and atractylenolide III (NF-κB-inhibited tight junction restoration) (Zheng et al., 2014), while reducing IL-6 via NF-κB/MAPK pathway regulation (Song et al., 2023). Tiaoqi Tongfu Tang alleviates systemic inflammation by magnolol (blocking TNF-α-induced NF-κB activation) and forsythoside A (inhibiting p38/JNK/MAPK pathways), and enhances peristalsis via emodin (M3 receptor activation) (Zhang et al., 2021; Song and Lei, 2025). Dahuang Fuzi Tang repairs mucosal barriers by emodin (suppressing NLRP3 inflammasome) and aconitum alkaloids (upregulating ZO-1/Occludin via PI3K/AKT/NF-κB inhibition) (Fu et al., 2025), while Xuebijing modulates gut microbiota (increasing Akkermansia/*Lactobacillus*) and suppresses pro-inflammatory cytokines via PI3K-AKT/NF-κB pathways (Ding et al., 2023; Chen et al., 2024).Detailed mechanistic pathways and extended background information are provided in the Supplementary Material for completeness.

Looking ahead, several critical directions must be prioritized to advance the application of Chinese medicine in sepsis treatment. First, additional high-quality RCTs are needed to elucidate the efficacy differences and dose–response relationships among various Chinese botanical drug interventions, including key clinical endpoints such as organ function recovery time and long-term survival rates, in order to strengthen the evidence base for clinical decision-making. Second, integration of network pharmacology with multi-omics technologies such as proteomics and metabolomics is required to explore the synergistic mechanisms, molecular targets, and signaling pathways of multi-metabolite formulations. This will facilitate a systematic understanding of the multi-target integrative regulatory mechanisms inherent to TCM. Lastly, better integration between basic research and clinical application is needed to guide the development of scientifically rigorous and standardized efficacy evaluation systems. Such efforts will help advance the global academic recognition and clinical utilization of Chinese medicine. Collectively, these strategies will contribute to elucidating the mechanistic basis of Chinese therapies, optimizing integrative medicine strategies and clinical care pathways, and ultimately improving clinical outcomes for patients with sepsis.

## 5 Strengths and limitations

This study conducted a systematic review and network meta-analysis incorporating 74 RCTs, enrolling a total of 5,898 patients, to systematically evaluate the differential efficacy of Chinese botanical drug interventions for sepsis-associated gastrointestinal dysfunction. The primary strength lies in the large pooled sample size and the construction of a comprehensive evidence network encompassing multiple interventions, enabling a robust comparison of the relative efficacy and safety of Chinese medicine versus conventional therapy, as well as among various botanical drug formulas. These findings provide updated evidence-based guidance for clinical decision-making. Additionally, this study systematically integrates evidence on TCM interventions, addressing a critical gap in the evidence base for integrative medicine in the management of sepsis-related gastrointestinal dysfunction.

However, this study has several limitations. Although standardized diagnostic criteria were employed to control study quality, heterogeneity remained due to regional differences, inconsistencies in baseline patient characteristics, and variability in intervention protocols, such as differences in botanical drug formula composition and timing of administration. Some botanical drug formulas, including modified Chengqi Tang and Zengye Chengqi Tang, were supported by only a limited number of independent studies and were often administered as multi-botanical drug prescriptions, lacking detailed mechanistic evaluation of individual active metabolites. Moreover, a large proportion of included trials were small or moderate in sample size, and high-quality direct comparisons between specific interventions were scarce, which may limit the precision of effect estimates. In addition, most studies did not clearly report the exact timing between sepsis onset and intervention initiation or the precise treatment duration, which limits the assessment of temporal and dose–response effects. Moreover, most studies did not conduct systematic monitoring of adverse events or clearly define the observation period, which limits the assessment of potential herb–drug interactions and the overall safety of TCM interventions in ICU patients. At last, a limitation of this study is the lack of data on mortality, ventilator-free days, ICU/hospital LOS, and enteral feeding tolerance in the included studies.

Readers are advised to interpret the findings with caution in the context of clinical practice, taking into account the balance between clinical benefits and potential risks of the interventions. Future research should prioritize the conduct of high-quality, standardized RCTs to expand the direct comparative evidence across different Chinese botanical drug therapies. Additionally, studies should incorporate long-term outcome measures such as organ function recovery time and 28-day survival rates, in order to build a more comprehensive evidence base for evidence-based clinical practice.

## 6 Conclusion

This study performed a network meta-analysis to systematically evaluate RCTs on Chinese medicine interventions for sepsis-associated gastrointestinal dysfunction. The findings demonstrated significant clinical benefits over conventional therapy in improving gastrointestinal dysfunction, systemic inflammation, and mucosal barrier impairment. Subgroup analysis revealed distinctive efficacy profiles across botanical drug formulations: Dachaihu Tang, Yiqi Tang, and Tiaoqi Tongfu Tang showed the most pronounced effects in improving gastrointestinal motility disorders; Tiaoqi Tongfu Tang and Houpo Heji exhibited superior efficacy in controlling systemic inflammatory responses; while Sijunzi Tang and Xuebijing were effective in promoting mucosal barrier repair. These findings support a stratified and personalized treatment approach based on distinct pathophysiological features—recommending Dachaihu Tang as the first-line therapy for patients with gastrointestinal motility impairment, Houpo Heji and Tiaoqi Tongfu Tang for those with marked systemic inflammation, and Sijunzi Tang or Xuebijing for patients with significant mucosal barrier damage. However, these conclusions warrant further validation through rigorously designed, large-scale, and long-term multicenter RCTs to quantify the efficacy differentials more precisely, elucidate underlying mechanisms, and facilitate the standardized and personalized application of Chinese medicine in the management of sepsis.

## Data Availability

The original contributions presented in the study are included in the article/Supplementary Material, further inquiries can be directed to the corresponding author.
