# Supplementary material for: The impact of traditional Chinese medicine on gastrointestinal dysfunction in sepsis patients: a systematic review and network meta-analysis
Source: Front Pharmacol. 2025 Oct 10;16:1648809. doi: 10.3389/fphar.2025.1648809 (PMC12549693; doi:10.3389/fphar.2025.1648809)
Supplement: Supplementary file 1 [file Supplementaryfile1.docx]

**Supplementary Table 1. Population, Index, Comparator, Outcomes, Timing and Setting (PICOTS) framework for the systematic review’s scope and eligibility criteria for inclusion.**

| Population |
| --- |
| Adult sepsis patients with gastrointestinal dysfunction. |
| Intervention |
| 16 traditional Chinese medicine prescriptions for intervention (such as Dachaihu Tang, Tiaoqi Tongfu Tang, Yiqi Tang, Dahuang Fuzi Tang, Xuebijing Injection, etc.). |
| Comparator |
| Conventional Western medicine treatment (including early fluid resuscitation, antibiotics, vasoactive drugs, nutritional support, etc.). |
| Outcome |
| Gastrointestinal dysfunction score, TCM symptom score, Apache Ⅱ score, AGI classification, D-lactic acid, DAO mean, TNF-a, IL-6, IAP, etc. |
| Timing |
| The intervention time varies depending on the study, generally between 3-14 days |
| Setting |
| The intensive care unit of the hospital |

**Supplementary Table 2A. Search strategy on PubMed and EMBASE.**

| #1 | "Medicine, Chinese Traditional"[MeSH] |
| --- | --- |
| #2 | ((((((((((((((Chinese Traditional Medicine[Title/Abstract]) OR (Traditional Chinese Medicine[Title/Abstract])) OR (Chinese Medicine, Traditional[Title/Abstract])) OR (Drugs, Chinese Herbal[Title/Abstract])) OR (Medicine, Traditional[Title/Abstract])) OR (Medicine, East Asian Traditional[Title/Abstract])) OR (Medicine, Chinese Traditional[Title/Abstract])) OR (Chinese herbal medicine[Title/Abstract])) OR (Drugs, Chinese Herbal[Title/Abstract])) OR (Chinese Drugs, Plant[Title/Abstract])) OR (Chinese Herbal Drugs[Title/Abstract])) OR (Herbal Drugs, Chinese[Title/Abstract])) OR (Plant Extracts, Chinese[Title/Abstract])) OR (Chinese Plant Extracts[Title/Abstract])) OR (Extracts, Chinese Plant[Title/Abstract]) |
| #3 | #1 OR #2 |
| #4 | Gastrointestinal diseases[MeSH] |
| #5 | (((((((((Gastrointestinal dysfunction[Title/Abstract]) OR (Gastrointestinal function[Title/Abstract])) OR (Gastrointestinal Impairment[Title/Abstract])) OR (Gastrointestinal Abnormality[Title/Abstract])) OR (Gastrointestinal Malfunction[Title/Abstract])) OR (Gastrointestinal Derangement[Title/Abstract])) OR (Disruption[Title/Abstract])) OR (Failure[Title/Abstract])) OR (Dysregulation[Title/Abstract])) OR (Malfunctioning[Title/Abstract]) |
| #6 | #4 OR #5 |
| #7 | Sepsis[MeSH] |
|  | #3 AND #6 AND #7 |

**Note:** Search performed up to April 2025 in PubMed and EMBASE. No language restrictions applied. #1, #2, etc. indicate individual search steps using MeSH/EMTREE or Title/Abstract terms combined with Boolean operators.

**Supplementary Table 2B. Search strategy on the Cochrane Central Register of Controlled Trials (CENTRAL).**

| #1 | MeSH descriptor: [Medicine, Chinese Traditional] this term only |
| --- | --- |
| #2 | (chinese traditional medicine OR traditional chinese medicine OR chinese medicine, traditional OR drugs, chinese herbal OR medicine, traditional OR medicine, east asian traditional OR medicine, chinese traditional OR chinese herbal medicine OR drugs, chinese herbal OR chinese drugs, plant OR chinese herbal drugs OR herbal drugs, chinese OR plant extracts, chinese OR chinese plant extracts OR extracts, chinese plant):ti,ab,kw |
| #3 | #1 OR #2 |
| #4 | MeSH descriptor: [Gastrointestinal Diseases] this term only |
| #5 | (gastrointestinal dysfunction OR gastrointestinal function OR gastrointestinal impairment OR gastrointestinal abnormality OR gastrointestinal malfunction OR gastrointestinal derangement OR disruption OR failure OR dysregulation OR malfunctioning):ti,ab,kw |
| #6 | #4 OR #5 |
| #7 | MeSH descriptor: [Sepsis] this term only |
| #8 | #3 AND #6 AND #7 |

**Note**: Search performed up to April 2025 in CENTRAL. No language restrictions applied. #1, #2, etc. indicate individual search steps using Title/Abstract terms combined with Boolean operators.

**Supplementary Table 2C. Search strategy on Web of Science.**

| #1 | TS=("Medicine, Chinese Traditional" OR "Chinese Traditional Medicine" OR "Traditional Chinese Medicine" OR "Chinese Medicine, Traditional" OR "Drugs, Chinese Herbal" OR "Medicine, Traditional" OR "Medicine, East Asian Traditional" OR "Chinese herbal medicine" OR "Chinese Drugs, Plant" OR "Chinese Herbal Drugs" OR "Herbal Drugs, Chinese" OR "Plant Extracts, Chinese" OR "Chinese Plant Extracts" OR "Extracts, Chinese Plant") |
| --- | --- |
| #2 | TS=("Gastrointestinal diseases" OR "Gastrointestinal dysfunction" OR "Gastrointestinal function" OR "Gastrointestinal Impairment" OR "Gastrointestinal Abnormality" OR "Gastrointestinal Malfunction" OR "Gastrointestinal Derangement" OR "Disruption" OR "Failure" OR "Dysregulation" OR "Malfunctioning") |
| #3 | TS=(Sepsis) |
| #4 | #1 AND #2 AND #3 |

**Note:** Search performed up to April 2025 in Web of Science. No language restrictions applied. #1, #2, etc. indicate individual search steps using Title/Abstract terms combined with Boolean operators.

**Supplementary Table 2D. Search strategy on CNKI.**

| SU=('Traditional Chinese Medicine'+'Traditional Chinese Medicine'+'Traditional Chinese Medicine'+'Traditional Chinese Medicine'+'Chinese Herbal Medicine'+'Herbal Medicine'+'Plant Extract') AND SU=('Gastrointestinal Diseases'+' Gastrointestinal Diseases'+'Gastrointestinal Dysfunction'+'Gastrointestinal Dysfunction'+'Gastrointestinal Function'+'Gastrointestinal Injury'+'Gastrointestinal Dysfunction') AND SU=('Sepsis'+' Septicemia ') |
| --- |

**Note**: Search performed up to April 2025 in CNKI. No language restrictions applied. Search terms were entered directly in Title, Keywords, or Abstract fields using Boolean operators; step numbers are not applicable.

**Supplementary Table 3. Drug Composition and Preparation Details**

| Drug Name | Ingredients (Latin Name, Family) and Dosage | Source / Supplier | Preparation Method | Administration Route / Method of Administration | Dosage and Administration |
| --- | --- | --- | --- | --- | --- |
| Banxia Xiexin Tang | *Pinelliae Rhizoma* (Banxia), *Aurantii Fructus Immaturus* (Zhishi) 15 g each; *Zingiberis Rhizoma* (Ganjiang), *Scutellariae Radix* (Huangqin), *Coptidis Rhizoma* (Huanglian) 12 g each; *Ginseng Radix et Rhizoma* (Renshen), *Trichosanthis Fructus* (Gualou) 30 g each; *Rhei Radix et Rhizoma* (Dahuang) 9 g; *Notoginseng Radix et Rhizoma* (Sanqi) 3 g; *Glycyrrhizae Radix et Rhizoma Praeparata Cum Melle* (Zhigancao) 6 g; *Jujubae Fructus* (Dazao) 3 pieces. | Hospital Pharmacy | Add clear water, decoct over gentle heat, and collect 400 ml of the medicinal decoction. | Oral Administration or Nasogastric Feeding | 400ml qd |
| Intestinal Comfort | *Rhei Radix et Rhizoma* (Dahuang) 9 g; *Taraxaci Herba* (Pugongying) 20 g; *Angelicae Sinensis Radix* (Danggui) 15 g; *Paeoniae Radix Rubra* (Chishao) 12 g; *Paeoniae Radix Alba* (Baishao) 12 g; *Chuanxiong Rhizoma* (Chuanxiong) 9 g; *Agrimoniae Herba* (Xianhecao) 20 g; *Coptidis Rhizoma* (Chuanhuanglian, Sichuan-origin Coptis) 6 g; *Pinelliae Rhizoma Praeparatum* (Qingbanxia) 6 g; *Trichosanthis Fructus* (Gualou) 15 g; *Sanguisorbae Radix* (Diyu) 20 g; *Poria* (Fuling) 20 g. | Hospital Pharmacy | Decoct intensively to obtain 150 ml of concentrated medicinal decoction. | Oral Administration or Nasogastric Feeding | 75ml bid |
| Dachaihu Tang | *Bupleuri Radix* (Chaihu) 15 g; *Scutellariae Radix* (Huangqin) 9 g; *Paeoniae Radix Alba* (Baishao) 9 g; *Aurantii Fructus Immaturus* (Zhishi) 9 g; *Zingiberis Rhizoma Recens* (Shengjiang) 15 g; *Pinelliae Rhizoma* (Banxia) 9 g; *Rhei Radix et Rhizoma* (Dahuang) 6 g; *Jujubae Fructus* (Dazao) 4 pieces (approximately 12 g). | Hospital Pharmacy | Decocted in the Hospital Traditional Chinese Medicine Pharmacy, and decocted down to 200 ml. | Oral Administration or Nasogastric Feeding | 100ml bid |
| Dachengqi Tang | *Rhei Radix et Rhizoma* (Dahuang, added near the end of decoction) 12 g; *Natrii Sulfas* (Mangxiao, taken with decoction) 9 g; *Aurantii Fructus Immaturus* (Zhishi) 12 g; *Magnoliae Officinalis Cortex* (Houpo) 12 g; *Bupleuri Radix* (Chaihu) 9 g; *Paeoniae Radix Alba* (Baishao) 12 g; *Scutellariae Radix* (Huangqin) 12 g; *Scrophulariae Radix* (Xuanshen) 15 g; *Gardeniae Fructus* (Zhizi) 10 g; *Salviae Miltiorrhizae Radix et Rhizoma* (Danshen) 15 g. | Hospital Pharmacy | Decocted in the Hospital Traditional Chinese Medicine Pharmacy, and decocted down to 300 ml. | Oral Administration or Nasogastric Feeding | 150ml bid |
| Rhubarb | *Rhei Radix et Rhizoma* (Dahuang) 60 g. | Hospital Pharmacy | Add water for decoction, and obtain approximately 150–200 ml of the medicinal decoction. | Retention Enema | 150ml qd |
| Dahuang Fuzi Tang | *Aconiti Lateralis Radix Praeparata* (Fuzi) 12 g; *Rhei Radix et Rhizoma* (Dahuang) 9 g; *Asari Radix et Rhizoma* (Xixin) 3 g. | Hospital Pharmacy | Decoct with water down to 400 ml. | Oral Administration or Nasogastric Feeding | 200ml bid |
| Dahuang Gancao Tang | *Rhei Radix et Rhizoma* (Shengdahuang, unprocessed Rheum) 30 g; *Glycyrrhizae Radix et Rhizoma* (Shenggancao, unprocessed Glycyrrhiza) 15 g. | Hospital Pharmacy | Decoct with water down to 100 ml. | Retention Enema | 100ml qd |
| Dahuang Mudan Tang | *Rhei Radix et Rhizoma* (Dahuang) 12 g; *Moutan Cortex* (Mudanpi) 3 g; *Benincasae Semen* (Dongguaren) 30 g; *Persicae Semen* (Taoren), *Natrii Sulfas* (Mangxiao) 9 g each. | Hospital Pharmacy | Decoct with water down to 100 ml. | Oral Administration or Nasogastric Feeding | 100ml tid |
| Tiaoqi Tongfu Tang | *Lonicerae Japonicae Flos* (Jinyinhua) 20 g; *Forsythiae Fructus* (Lianqiao) 10 g; *Rhei Radix et Rhizoma* (Dahuang) 10 g; *Aurantii Fructus Immaturus* (Zhishi) 15 g; *Magnoliae Officinalis Cortex* (Houpo) 15 g; *Glycyrrhizae Radix et Rhizoma Praeparata Cum Melle* (Zhigancao) 15 g; *Aucklandiae Radix* (Muxiang) 10 g; *Arecae Semen* (Binglang) 10 g; *Salviae Miltiorrhizae Radix et Rhizoma* (Danshen) 15 g; *Persicae Semen* (Taoren) 15 g; *Crataegi Fructus Carbonisatus* (Jiaoshanzha, charred Hawthorn) 10 g; *Massae Fermentatae Medicinalis Carbonisatus* (Jiaoshenqu, charred Medicinal Leaven) 10 g; *Hordei Fructus Germinatus Carbonisatus* (Jiaomaiya, charred Germinated Barley) 10 g. | Hospital Pharmacy | Decoct with water down to 400 ml. | Oral Administration or Nasogastric Feeding | 200ml bid |
| Houpo Heji | *Magnoliae Officinalis Cortex Praeparata Cum Zingibere et Salibus* (Houpo, processed with ginger); *Aucklandiae Radix* (Muxiang); *Aurantii Fructus Immaturus Praeparata Cum Farinae Triticinae* (Zhishi, stir-fried with wheat bran); *Rhei Radix et Rhizoma* (Dahuang). | Ruiyang Pharmaceutical Co., Ltd. | Take (the medicinal powder or granule) with (warm) water. | Oral Administration or Nasogastric Feeding | 50ml bid |
| Sijunzi Tang | *Ginseng Radix et Rhizoma* (Renshen) 9 g; *Atractylodis Macrocephalae Rhizoma* (Baizhu) 9 g; *Poria* (Fuling) 9 g; *Glycyrrhizae Radix et Rhizoma Praeparata Cum Melle* (Zhigancao) 6 g. | Hospital Pharmacy | Decoct over gentle heat to obtain 300 ml of the medicinal decoction. | Oral Administration or Nasogastric Feeding | 150ml bid |
| Liangge San | *Hordei Fructus Germinatus* (Shengmaiya, unprocessed Germinated Barley) 15 g; *Gallus Gallus Domesticus Endothelium Corneum* (Shengjinneijin, unprocessed Chicken Gizzard Lining) 15 g; *Zingiberis Rhizoma* (Ganjiang) 6 g; *Scutellariae Radix* (Huangqin) 15 g; *Menthae Haplocalycis Herba* (Bohe, added at the beginning of decoction) 9 g; *Gardeniae Fructus* (Zhizi) 3 g; *Phyllostachydis Henonis Folium* (Zhuye) 12 g; *Rhei Radix et Rhizoma* (Dahuang, added near the end of decoction) 3 g; *Natrii Sulfas* (Mangxiao, taken with decoction) 6 g; *Glycyrrhizae Radix et Rhizoma Praeparata Cum Melle* (Zhigancao) 15 g. | Hospital Pharmacy | Decoct with water down to 250 ml. | Oral Administration or Nasogastric Feeding | 125ml bid |
| Xuebijing | *Carthami Flos* (Honghua); *Paeoniae Radix Rubra* (Chishao); *Chuanxiong Rhizoma* (Chuanxiong); *Salviae Miltiorrhizae Radix et Rhizoma* (Danshen); *Angelicae Sinensis Radix* (Danggui). | Tianjin Hongri Pharmaceutical Co., Ltd. | Add 50 ml (of the medicinal solution) to 100 ml of normal saline. | Intravenous Infusion | 50ml bid |
| Yantiao Fang | *Natrii Sulfas* (Mangxiao) 10 g; *Angelicae Sinensis Radix* (Danggui) 12 g; *Persicae Semen* (Taoren) 12 g; *Forsythiae Fructus* (Lianqiao) 15 g; *Scrophulariae Radix* (Xuanshen) 12 g; *Paeoniae Radix Rubra* (Chishao) 15 g; *Lonicerae Japonicae Flos* (Yinhua, Honeysuckle Flower) 15 g; *Rhei Radix et Rhizoma* (Shengdahuang, unprocessed Rheum, added near the end of decoction) 6 g; *Ophiopogonis Radix* (Maidong) 12 g. | Hospital Pharmacy | Decocted in the Hospital Traditional Chinese Medicine Pharmacy, and decocted down to 200 ml. | Oral Administration or Nasogastric Feeding | 100ml bid |
| Yiqi Tang | *Codonopsis Radix* (Dangshen) 30 g; *Astragali Radix* (Huangqi) 30 g; *Atractylodis Macrocephalae Rhizoma* (Baizhu) 30 g; *Poria* (Fuling) 30 g; *Chuanxiong Rhizoma* (Chuanxiong) 30 g; *Angelicae Sinensis Radix* (Danggui) 30 g; *Rehmanniae Radix Praeparata* (Shudihuang) 30 g; *Paeoniae Radix Alba* (Baishao) 30 g; *Glycyrrhizae Radix et Rhizoma Praeparata Cum Melle* (Zhigancao) 30 g; *Salviae Miltiorrhizae Radix et Rhizoma* (Danshen) 15 g; *Rhei Radix et Rhizoma* (Dahuang) 10 g; *Moutan Cortex* (Mudanpi) 5 g; *Persicae Semen* (Taoren) 10 g; *Benincasae Semen* (Dongguaren) 30 g. | Hospital Pharmacy | Decoct with water down to 300 ml. | Oral Administration or Nasogastric Feeding | 150ml bid |
| Zengye Chengqi Tang | *Scrophulariae Radix* (Xuanshen) 15 g; *Ophiopogonis Radix* (Maidong) 15 g; *Rehmanniae Radix* (Shengdihuang, unprocessed Rehmannia) 15 g; *Rhei Radix et Rhizoma* (Dahuang) 15 g; *Natrii Sulfas* (Mangxiao) 10 g. | Hospital Pharmacy | Decocted in the Hospital Traditional Chinese Medicine Pharmacy, and decocted down to 200 ml. | Oral Administration or Nasogastric Feeding | 200ml qd |

**Note:** All single herbs in the table are reported using the standard Latin names from the pharmacopeia.The formulas listed in the table are representative prescriptions of each category, with their composition and dosage reflecting the core structure and therapeutic rationale of the category. In practice, the prescriptions used in individual studies may vary according to clinical needs, including the addition or removal of single herbs, dosage adjustments, or differences in processing methods. This table provides a methodological overview and is not intended to list all included prescriptions in detail.

**Supplementary Table 4. Characteristics of the studies included in the meta-analysis**

| Author | Year | Total/Male/Female | Age（mean+SD） | Intervention | | Treatment  course | Outcome | Apache Ⅱ score |
| --- | --- | --- | --- | --- | --- | --- | --- | --- |
|  |  |  |  | Intervention | Control |  |  |  |
| Zhang Feihu | 2016 | T:40/22/18 C:40/21/19 | T:57.11(12.15) C:56.89(11.79) | Banxia Xiexin Tang | CON | 14d | ①③ | T:11.78 C:17.28 |
| Wang Lina | 2016 | T:30 C:30 | NA | Banxia Xiexin Tang | CON | NA | ①② | NA |
| He Shaowu | 2020 | T:154/75/79 C:154/80/74 | T:43.5(5.1) C:45.7(5.4) | Banxia Xiexin Tang | CON | 7d | ①③ | T:13.08 C:17.11 |
| Yang Hailing | 2019 | T:38/22/16 C:38/20/18 | T:45.28(4.89) C:46.71(4.05) | Banxia Xiexin Tang | CON | 14d | ① | NA |
| Li Guochen | 2020 | T:31/20/11 C:31/23/8 | T:76.70(12.13) C:75.38(12.55) | Banxia Xiexin Tang | CON | 7d | ①②③⑤⑥⑨ | T:11.58 C:13.00 |
| Liu Weihong | 2019 | T:30/18/12 C:30/15/15 | T:60.8(14.65) C:61.0(14.49) | Banxia Xiexin Tang | CON | 7d | ①②③⑦⑧⑨ | T:13.63 C:17.80 |
| Zhang Gaofeng | 2018 | T:34/19/15 C:33/18/15 | T:47.82(4.54) C:47.69(4.13) | Banxia Xiexin Tang | CON | 14d | ①③ | T:30.77 C:25.17 |
| Qin Fengzhou | 2023 | T:35/18/17 C:35/15/20 | T:60.67(10.93) C:60.15(10.33) | Banxia Xiexin Tang | CON | 14d | ①②③⑤⑥⑨ | T:11.61 C:13.78 |
| Li Wenjing | 2012 | T:40/22/18 C:40/24/16 | T:71.98(11.72) C:72.95(11.07) | Banxia Xiexin Tang | CON | 14d | ①②③ | T:11.98 C:17.28 |
| Gao Haiyun | 2016 | T:25/16/9 C:25/14/11 | T:47.56(8.37) C:48.96(10.25) | Intestinal Comfort | CON | 5d | ①③⑤⑥⑦⑧ | T:10.72 C:11.48 |
| Chen Tingting | 2017 | T:60/36/24 C:60/32/28 | T:55.33(14.59) C:56.26(13.94) | Intestinal Comfort | CON | 7d | ①③⑨ | T:11.56 C:14.48 |
| Chen Fenqiao | 2016 | T:25/12/13 C:25/10/15 | T:43.17(13.68) C:41.32(13.57) | Intestinal Comfort | CON | 7d | ⑤⑥⑦⑧ | NA |
| Zhong Yuanfang | 2016 | T:20/11/9 C:20/8/12 | T:52.50(1.06) C:52.30(1.40) | Dachaihu Tang | CON | 7d | ①②③ | T:10.63 C:16.32 |
| Chen Yanping | 2019 | T:46/30/16 C:46/29/17 | T:44.7(7.3) C:46.8(6.5) | Dachengqi Tang | CON | 4d | ①③⑨ | T:13.41 C:16.37 |
| Cheng Xiangjin | 2022 | T:17/10/7 C:17/9/8 | T:67.54(7.31) C:65.48(6.93) | Dachengqi Tang | CON | 7d | ①③⑨ | T:12.02 C:16.44 |
| Hu Minglei | 2019 | T:30/15/15 C:30/14/16 | T:57.3(7.3) C:58.2(7.6) | Rhubarb | CON | 14d | ①③⑨ | T:9.47 C:13.86 |
| Sun Jie | 2019 | T:41/22/19 C:41/20/21 | T:47.0(5.6) C:46.0(5.4) | Rhubarb | CON | 7d | ①③⑨ | T:8.30 C:13.70 |
| Zhang Li | 2017 | T:60/29/31 C:60/32/28 | T:57.26(7.31) C:58.04(7.42) | Rhubarb | CON | 7d | ①③⑨ | T:8.47 C:13.82 |
| Yan Yun | 2019 | T:38 C:38 | T:65(5) C:63(6) | Rhubarb | CON | 7d | ①③⑨ | T:11.00 C:15.00 |
| Fang Kun | 2009 | T:30/19/11 C:30/17/11 | T:58(14) C:60(15) | Rhubarb | CON | 5d | ⑦⑧ | NA |
| Zhou Wen | 2021 | T:28/16/12 C:35/20/15 | T:64.5(10.3) C:62.4(11.3) | Rhubarb | CON | 7d | ⑨ | NA |
| Yang Ligai | 2021 | T:35/25/10 C:35/24/11 | T:46.13(3.23) C:45.13(3.23) | Rhubarb | CON | 14d | ①③⑨ | T:8.52 C:13.75 |
| Wu Shubing | 2023 | T:30/17/13 C:32/17/15 | T:65.5(5.3) C:62.4(3.3) | Rhubarb | CON | 7d | ③④⑨ | T:16.30 C:17.70 |
| Fu Tian | 2016 | T:47/29/18 C:47/31/16 | T:57.63(7.82) C:58.19(6.74) | Rhubarb | CON | 7d | ①③⑨ | T:8.41 C:13.71 |
| Yin Zhong | 2021 | T:45/23/22 C:45/24/21 | T:56.63(7.72) C:57.32(6.32) | Rhubarb | CON | 7d | ①③ | T:7.34 C:13.54 |
| Yin Chao | 2015 | T:32/19/13 C:36/21/15 | T:50.0(7.1) C:53.0(7.9) | Rhubarb | CON | 7d | ⑦⑧ | NA |
| Li Duolei | 2013 | T+C:48/26/22 | T+C:55.4 | Rhubarb | CON | 7d | ③ | T:9.20 C:15.60 |
| Huang Lu | 2021 | T:48/29/19 C:48/27/21 | T:47.57(4.35) C:48.36(5.69) | Rhubarb | CON | 7d | ①③⑤⑥⑧ | T:12.09 C:19.73 |
| Zhou Gang | 2011 | T+C:48/26/22 | T+C:49.22(14.20) | Rhubarb | CON | 7d | ①③ | T:11.22 C:21.02 |
| Hu Xiong | 2019 | T:40/27/13 C:40/28/12 | T:54.83(11.27) C:54.27(11.08) | Dahuang Fuzi Tang | CON | 14d | ①③④ | T:8.48 C:12.74 |
| Liu Jin | 2022 | T:30/14/6 C:30/15/5 | T:80.93(9.42) C:81.63(8.64) | Dahuang Fuzi Tang | CON | 6d | ④⑤ | NA |
| Liu Yongcheng | 2019 | T:40/21/19 C:40/22/18 | T:51.6(1.5) C:51.3(1.4) | Dahuang Fuzi Tang | CON | 14d | ①③④ | T:8.49 C:13.11 |
| Liang Futing | 2017 | T:39/23/16 C:39/25/14 | T:57.27(2.86) C:57.21(2.83) | Dahuang Fuzi Tang | CON | 14d | ①③④ | T:8.51 C:12.65 |
| Wang Kaiwu | 2022 | T:42/24/18 C:42/25/17 | T:47.69(5.38) C:47.72(5.36) | Dahuang Fuzi Tang | CON | 14d | ①②③ | T:10.35 C:14.42 |
| Luo Xiaopei | 2023 | T:30/17/13 C:30/19/11 | T:55.35(3.05) C:55.28(2.98) | Dahuang Fuzi Tang | CON | 7d | ①③ | T:10.48 C:13.65 |
| Li Jun | 2019 | T:50/27/23 C:50/26/24 | T:51.87(8.58) C:51.34(8.21) | Dahuang Fuzi Tang | CON | 7d | ①②③ | T:10.54 C:15.43 |
| Xu Jieru | 2021 | T:50/26/24 C:50/28/22 | T:53.23(5.63) C:55.32(3.12) | Dahuang Fuzi Tang | CON | 15d | ①③ | T:10.04 C:12.52 |
| Li Xionghui | 2017 | T:60/38/22 C:60/33/27 | T:57.16(2.78) C:56.23(3.14) | Dahuang Fuzi Tang | CON | 14d | ①③④ | T:8.53 C:12.64 |
| Han Dan | 2022 | T:35/21/14 C:35/24/11 | T:60.13(16.54) C:NA | Dahuang Fuzi Tang | CON | 14d | ②③④⑦⑧⑨ | T:5.2 C:6.77 |
| Liu Zhiyong | 2022 | T:35/18/17 C:35/19/16 | T:54.03(5.63) C:53.87(5.72) | Dahuang Fuzi Tang | CON | 7d | ①③④ | T:8.75 C:11.31 |
| Luo Yuanfeng | 2015 | T:30/17/13 C:30/16/14 | T:51.51(13.96) C:51.28(14.05) | Dahuang Fuzi Tang | CON | 7d | ①②③ | T:10.52 C:15.56 |
| Qin Wenbo | 2014 | T+C:60/38/22 | T+C:46.58(8.99) | Dahuang Gancao Tang | CON | 3d | ① | NA |
| Fu Zheng | 2007 | T:30/16/14 C:30/13/17 | NA | Dahuang Gancao Tang | CON | 3d | ① | NA |
| Yao Keyu | 2008 | T+C:87/47/40 | NA | Dahuang Gancao Tang | CON | 7d | ① | NA |
| Xie Changhui | 2023 | T:38/23/15 C:38/26/12 | T:58.32(5.48) C:59.61(7.32) | Dahuang Mudan Tang | CON | 7d | ①③⑤⑦ | T:11.15 C:16.23 |
| Zhang Xiaoyun | 2023 | T:50/28/22 C:50/31/19 | T:48.23(7.23) C:48.17(7.22) | Dahuang Mudan Tang | CON | 7d | ③ | T:9.37 C:11.42 |
| Dangping | 2025 | T:46/26/20 C:46/24/22 | T:56.18(7.46) C:55.36(7.92) | Dahuang Mudan Tang | CON | 14d | ⑤ | NA |
| Hu Yongjie | 2025 | T:31/18/13 C:31/19/12 | T:53.15(2.06) C:52.78(2.94) | Dahuang Mudan Tang | CON | 7d | ⑦ | NA |
| Wu Xinyi | 2022 | T:33/18/15 C:33/15/18 | NA | Tiaoqi Tongfu Tang | CON | 7d | ③⑨ | T:21.09 C:21.56 |
| Sun Ke | 2019 | T:43/23/20 C:43/25/18 | T:55.02(5.31) C:54.95(5.28) | Tiaoqi Tongfu Tang | CON | 7d | ①⑤⑥ | NA |
| Le Lan | 2013 | NA | NA | Tiaoqi Tongfu Tang | CON | 7d | ②③⑨ | T:11.50 C:15.90 |
| Huang Zhiyong | 2012 | T:34/18/16 C:34/17/17 | T:61.2(15.3) C:60.5(17.2) | Tiaoqi Tongfu Tang | CON | 7d | ②③⑨ | T:11.40 C:15.40 |
| Li Xingxing | 2022 | T:30/16/14 C:30/13/17 | T:65.83(10.45) C:65.58(10.63) | Tiaoqi Tongfu Tang | CON | 7d | ①②③ | T:13.45 C:17.76 |
| Wang Hui | 2024 | T:45/22/23 C:45/25/20 | T:74.13(2.16) C:74.09(2.14) | Tiaoqi Tongfu Tang | CON | 7d | ③⑦⑧ | T:40.12 C:59.34 |
| Shen Limin | 2021 | T:68/28/40 C:68/30/38 | T:63(8) C:62(9) | Houpo Heji | CON | 7d | ①⑨ | NA |
| Li Wei | 2022 | T:20/13/7 C:20/12/8 | T:63.35(12.54) C:69.10(8.20) | Houpo Heji | CON | 7d | ③ | T:13.40 C:17.50 |
| He Xinghua | 2024 | T:30/17/13 C:30/18/12 | T:70.20(5.88) C:70.23(5.86) | Houpo Heji | CON | 7d | ①③ | T:13.26 C:17.35 |
| Mao Shihao | 2024 | T:25/12/13 C:25/13/12 | T:59.16(13.22) C:58.68(12.14) | Houpo Heji | CON | 7d | ⑤⑥⑦⑧ | NA |
| Liu Yang | 2013 | T:30/17/13 C:30/16/14 | T:57.53(10.79) C:57.93(10.74) | Sijunzi Tang | CON | 7d | ①③⑥⑦⑧ | T:10.00 C:13.03 |
| Xu Lingqi | 2023 | T:30/16/14 C:30/17/13 | T:47.21(5.54) C:47.81(5.93) | Sijunzi Tang | CON | 14d | ⑤⑦ | NA |
| Nie Sha | 2022 | T:28/17/11 C:29/13/16 | T:59.96(12.79) C:61.59(13.12) | Sijunzi Tang | CON | 7d | ①②③⑨ | T:12.04 C:14.66 |
| Jin Yue | 2025 | T:41/19/22 C:41/20/21 | T:67.78(11.24) C:67.32(11.61) | Liangge San | CON | 7d | ③⑤⑥⑦⑧⑨ | T:11.15 C:16.63 |
| Shen Qing | 2016 | T:25/14/11 C:25/18/7 | T:71.4(11.82) C:70.12(13.88) | Liangge San | CON | 7d | ②③⑦ | T:17.56 C:18.40 |
| Zhou Ben | 2023 | T:27/15/12 C:25/12/13 | T:62.33(8.24) C:63.08(8.78) | Xuebijing | CON | 5d | ②③⑨ | T:12.67 C:15.68 |
| Zhang Jian | 2019 | T:27/15/12 C:25/15/10 | T:72.41(2.44) C:71.36(3.60) | Xuebijing | CON | 7d | ①③⑥ | T:13.77 C:13.35 |
| Chen Qian | 2020 | T:31/19/12 C:31/25/6 | T:67.26(16.26) C:68.74(16.87) | Yantiao Fang | CON | 10d | ②③⑤⑥⑦⑧⑨ | T:14.35 C:17.13 |
| Jin Yuanyuan | 2021 | T:60/34/26 C:60/44/16 | T:67.93(16.04) C:70.35(14.91) | Yantiao Fang | CON | 7-14d | ②⑤⑦⑨ | NA |
| Wang Liu | 2021 | T:30/18/12 C:30/20/10 | T:73.73(8.88) C:72.53(10.75) | Yantiao Fang | CON | 7d | ①②③ | T:5.89 C:6.07 |
| Hou Yinan | 2024 | T:29/15/14 C:28/11/17 | T:66.41(9.94) C:65.29(10.79) | Yiqi Tang | CON | 7d | ①②③ | T:10.28 C:15.39 |
| Huang Zengfeng | 2019 | T:30/18/12 C:30/17/13 | T:57.32(8.62) C:56.71(9.14) | Yiqi Tang | CON | 7d | ①③⑦⑧ | T:12.58 C:17.67 |
| Zhang Zhiguang | 2022 | T:53/34/19 C:53/36/17 | T:47.06(8.71) C:46.82(9.03) | Yiqi Tang | CON | 7d | ①②③⑤ | T:12.14 C:18.75 |
| Du Yongming | 2022 | T:30/18/12 C:30/17/13 | T:42.29(5.57) C:42.17(5.94) | Zengye Chengqi Tang | CON | 14d | ①②③⑦ | T:11.25 C:14.06 |
| Xia Yichun | 2024 | T:40/27/13 C:40/26/14 | T:64.06(11.32) C:63.47(9.58) | Zengye Chengqi Tang | CON | 7d | ④⑤⑥ | / |
| Gao Weiwei | 2019 | T:30/17/13 C:30/15/14 | T:72.33(10.72) C:72.14(10.43) | Zengye Chengqi Tang | CON | 10d | ②③⑤⑥⑨ | T:10.07 C:15.50 |

**Note**: CON: conventional treatment, included early fluid resuscitation combined with close hemodynamic monitoring (e.g., monitoring of hemodynamic parameters); initial empiric broad-spectrum antimicrobial therapy with prompt switch to pathogen-sensitive antibiotics immediately upon confirmation of the pathogenic microorganism; administration of vasoactive agents where norepinephrine was the first-line choice and vasopressin could be added as an adjunctive agent to achieve the target mean arterial pressure (MAP); comprehensive supportive care encompassing host immune modulation, sedation management, continuous renal replacement therapy (CRRT), and nutritional support; as well as symptomatic treatments tailored to clinical needs, including improvement of intragastric pH value, protection of the gastrointestinal mucosal barrier, and correction of intestinal flora imbalance. T: experimental group, C: control group,T+C: The study did not separately report the age and gender ratios, only the overall age and gender ratios,NA: Not available,①:Gastrointestinal dysfunction score,②:Traditional Chinese Medicine Syndrome Score,③:APACHE II rating,④:AGI grading,⑤:D-lactic acid,⑥:DAO,⑦:TNF - α,⑧:IL-6,⑨:IAP

**Supplementary Table 5A. Node-specific Gastrointestinal dysfunction score**

| Side | Direct |  | Indirect |  | Difference |  |  | tau |
| --- | --- | --- | --- | --- | --- | --- | --- | --- |
| Coef. | Std. Err. | Coef. | Std. Err. | Coef. | Std. Err. | P>\|z\| |  |  |
| A C * | 0.7635856 | 0.1653985 | 0.0080914 | 49.95115 | 0.7554942 | 49.95145 | 0.988 | 0.4462251 |
| B C * | 0.3099999 | 0.4495037 | 1.217911 | 631.3658 | -0.9079107 | 631.366 | 0.999 | 0.4462248 |
| C D * | -2.03 | 0.9354767 | -3.557149 | 631.4634 | 1.527149 | 631.463 | 0.998 | 0.4462248 |
| C E * | -0.9137639 | 0.3172832 | -2.440312 | 440.7232 | 1.526548 | 440.7233 | 0.997 | 0.4462256 |
| C F * | -0.4964099 | 0.1434396 | -2.013063 | 203.183 | 1.516653 | 203.1831 | 0.994 | 0.4462249 |
| C G * | -1.030674 | 0.2660777 | -2.557048 | 361.3155 | 1.526374 | 361.3156 | 0.997 | 0.4462248 |
| C H * | -0.4400004 | 0.4583428 | -1.974955 | 623.9486 | 1.534954 | 623.9487 | 0.998 | 0.4462259 |
| C I * | -0.2503602 | 0.3208838 | -1.772416 | 448.1807 | 1.522056 | 448.1808 | 0.997 | 0.4462248 |
| C J * | -0.543165 | 0.3418224 | -2.073393 | 445.7065 | 1.530228 | 445.7066 | 0.997 | 0.446225 |
| C K * | -0.8355433 | 0.1513208 | -2.362818 | 215.4021 | 1.527275 | 215.4022 | 0.994 | 0.4462249 |
| C L * | -0.3250824 | 0.326931 | -1.852335 | 449.2921 | 1.527252 | 449.2922 | 0.997 | 0.4462248 |
| C M * | -0.4370762 | 0.3403212 | -1.964186 | 448.2218 | 1.52711 | 448.2219 | 0.997 | 0.4462249 |
| C N * | -0.66 | 0.521498 | -2.203341 | 627.764 | 1.543341 | 627.7641 | 0.998 | 0.4462251 |
| C O * | -0.579964 | 0.2711686 | -2.107102 | 361.7798 | 1.527138 | 361.7799 | 0.997 | 0.4462249 |
| C P * | -0.63 | 0.4496664 | -2.160167 | 629.7114 | 1.530167 | 629.7115 | 0.998 | 0.4462248 |

**Note**: **Side**: treatment comparison; **Direct/Indirect Coef. & Std. Err**.: effect estimates and standard errors from direct or indirect comparisons; **Difference**: difference between direct and indirect estimates, with SE and p-value; **tau**: between-study heterogeneity.

**Supplementary Table 5B. Node-specific TCM symptom score**

| Side | Direct |  | Indirect |  | Difference |  |  | tau |
| --- | --- | --- | --- | --- | --- | --- | --- | --- |
| Coef. | Std. Err. | Coef. | Std. Err. | Coef. | Std. Err. | P>\|z\| |  |  |
| A B * | 3.47624 | 0.9880952 | -0.000000356 | 74.53859 | 3.47624 | 74.54518 | 0.963 | 1.91143 |
| B C * | -4.17 | 2.225717 | -11.12132 | 632.3802 | 6.951316 | 632.3821 | 0.991 | 1.911365 |
| B D * | -5.309859 | 1.091327 | -12.26138 | 316.1136 | 6.95152 | 316.1146 | 0.982 | 1.911374 |
| B E * | -3.56 | 2.22406 | -10.51132 | 632.459 | 6.951318 | 632.4608 | 0.991 | 1.911367 |
| B F * | -4.02 | 2.634973 | -10.97134 | 632.5453 | 6.951337 | 632.5456 | 0.991 | 1.911364 |
| B G * | -3.705031 | 1.159452 | -10.65649 | 365.0726 | 6.951454 | 365.074 | 0.985 | 1.911374 |
| B H * | -1.74 | 1.96477 | -8.691312 | 632.324 | 6.951312 | 632.3267 | 0.991 | 1.911366 |
| B I * | -3.472416 | 1.213859 | -10.42387 | 365.312 | 6.951452 | 365.3133 | 0.985 | 1.911368 |
| B J * | -5.702547 | 2.111968 | -12.65393 | 447.1728 | 6.951386 | 447.1713 | 0.988 | 1.911369 |
| B K * | -4.037836 | 1.37326 | -10.98922 | 447.5869 | 6.951382 | 447.5889 | 0.988 | 1.911369 |

**Note**: **Side**: treatment comparison; **Direct/Indirect Coef. & Std. Err**.: effect estimates and standard errors from direct or indirect comparisons; **Difference**: difference between direct and indirect estimates, with SE and p-value; **tau**: between-study heterogeneity.

**Supplementary Table 5C. Node-specific Apache Ⅱ score**

| Side | Direct |  | Indirect |  | Difference |  |  | tau |
| --- | --- | --- | --- | --- | --- | --- | --- | --- |
| Coef. | Std. Err. | Coef. | Std. Err. | Coef. | Std. Err. | P>\|z\| |  |  |
| A C * | 2.293481 | 1.172974 | 0.0482486 | 45.18095 | 2.245233 | 45.19617 | 0.96 | 2.935232 |
| B C * | 1.182101 | 2.147816 | 3.4019 | 447.2545 | -2.219799 | 447.2603 | 0.996 | 2.935199 |
| C D * | -5.69 | 3.332851 | -10.274 | 632.5001 | 4.584 | 632.505 | 0.994 | 2.935199 |
| C E * | -3.645456 | 2.186385 | -8.229503 | 447.199 | 4.584047 | 447.2032 | 0.992 | 2.935197 |
| C F * | -3.655003 | 0.9268516 | -8.23963 | 190.8471 | 4.584627 | 190.849 | 0.981 | 2.935203 |
| C G * | -3.535907 | 2.107189 | -8.119968 | 447.2685 | 4.584061 | 447.2732 | 0.992 | 2.935199 |
| C H * | -4.094911 | 2.160714 | -8.679025 | 447.5402 | 4.584114 | 447.5446 | 0.992 | 2.935196 |
| C I * | -1.811399 | 2.114781 | -6.395461 | 447.1814 | 4.584062 | 447.186 | 0.992 | 2.9352 |
| C J * | -3.33449 | 2.26466 | -7.918551 | 447.2355 | 4.58406 | 447.2394 | 0.992 | 2.935201 |
| C K * | -5.25908 | 0.9336321 | -9.84373 | 190.6574 | 4.584649 | 190.6592 | 0.981 | 2.935203 |
| C L * | -2.828904 | 2.219595 | -7.412945 | 447.1918 | 4.58404 | 447.1959 | 0.992 | 2.935198 |
| C M * | -6.896225 | 1.410576 | -11.48051 | 282.9238 | 4.584283 | 282.9263 | 0.987 | 2.935201 |
| C N * | -1.453514 | 2.150306 | -6.037575 | 447.3865 | 4.58406 | 447.391 | 0.992 | 2.9352 |
| C O * | -5.642285 | 1.775479 | -10.22641 | 365.3039 | 4.584124 | 365.3075 | 0.99 | 2.9352 |
| C P * | -4.009624 | 2.18421 | -8.593691 | 447.058 | 4.584067 | 447.0623 | 0.992 | 2.935196 |

**Note**: **Side**: treatment comparison; **Direct/Indirect Coef. & Std. Err**.: effect estimates and standard errors from direct or indirect comparisons; **Difference**: difference between direct and indirect estimates, with SE and p-value; **tau**: between-study heterogeneity.

**Supplementary Table 5D. Node-specific D-lactic acid**

| Side | Direct |  | Indirect |  | Difference |  |  | tau |
| --- | --- | --- | --- | --- | --- | --- | --- | --- |
|  | Coef. | Std. Err. | Coef. | Std. Err. | Coef. | Std. Err. | P>\|z\| |  |
| A B * | 3.140663 | 1.154811 | -0.0000416 | 81.66911 | 3.140705 | 81.67728 | 0.969 | 1.586587 |
| B C * | -0.5295 | 1.608213 | -6.809619 | 632.2037 | 6.280119 | 632.2056 | 0.992 | 1.586552 |
| B D * | -1.339272 | 1.13573 | -7.619467 | 447.275 | 6.280195 | 447.2763 | 0.989 | 1.586554 |
| B E * | -0.8182323 | 1.61386 | -7.098378 | 631.5755 | 6.280146 | 631.5774 | 0.992 | 1.586552 |
| B F * | -2.427648 | 1.163452 | -8.707354 | 447.5617 | 6.279705 | 447.563 | 0.989 | 1.586554 |
| B G * | -0.6530742 | 1.602713 | -6.933257 | 632.6492 | 6.280182 | 632.6511 | 0.992 | 1.586552 |
| B H * | -1.601878 | 1.604019 | -7.88203 | 632.4575 | 6.280152 | 632.4594 | 0.992 | 1.586552 |
| B I * | -3.511805 | 1.641772 | -9.791955 | 632.621 | 6.28015 | 632.6229 | 0.992 | 1.586552 |
| B J * | -2.588933 | 1.613972 | -8.869032 | 632.2481 | 6.280099 | 632.25 | 0.992 | 1.586552 |
| B K * | -0.8714211 | 1.133672 | -7.151657 | 447.2266 | 6.280236 | 447.228 | 0.989 | 1.586554 |
| B L * | -1.839652 | 1.603619 | -8.119389 | 632.6407 | 6.279737 | 632.6426 | 0.992 | 1.586552 |
| B M * | -1.514839 | 1.140723 | -7.795035 | 447.4382 | 6.280195 | 447.4396 | 0.989 | 1.586554 |

**Note**: **Side**: treatment comparison; **Direct/Indirect Coef. & Std. Err**.: effect estimates and standard errors from direct or indirect comparisons; **Difference**: difference between direct and indirect estimates, with SE and p-value; **tau**: between-study heterogeneity.

**Supplementary Table 5E. Node-specific DAO mean**

| Side | Direct |  | Indirect |  | Difference |  |  | tau |
| --- | --- | --- | --- | --- | --- | --- | --- | --- |
|  | Coef. | Std. Err. | Coef. | Std. Err. | Coef. | Std. Err. | P>\|z\| |  |
| A C | 1.008882 | 1.138616 | 0.4153831 | 95.38873 | 0.5934993 | 95.39553 | 0.995 | 1.588534 |
| B C | 4.569251 | 1.679662 | -2.551092 | 632.1513 | 7.120342 | 632.154 | 0.991 | 1.588495 |
| C D | -2.848578 | 1.641029 | -4.865918 | 632.6632 | 2.01734 | 632.6651 | 0.997 | 1.588497 |
| C E | -2.153521 | 1.158681 | -4.170327 | 446.0976 | 2.016806 | 446.0989 | 0.996 | 1.588501 |
| C F | -1.49812 | 1.6083 | -3.515979 | 633.2939 | 2.017858 | 633.2959 | 0.997 | 1.588497 |
| C G | -1.32043 | 1.604539 | -3.338074 | 632.3652 | 2.017644 | 632.3671 | 0.997 | 1.588497 |
| C H | -1.842387 | 1.61886 | -3.860035 | 631.4208 | 2.017648 | 631.4227 | 0.997 | 1.588497 |
| C I | -2.29729 | 1.613169 | -4.31499 | 633.488 | 2.0177 | 633.49 | 0.997 | 1.588498 |
| C J | -0.7359752 | 1.610146 | -2.753595 | 632.2214 | 2.01762 | 632.2233 | 0.997 | 1.588497 |
| C K | -1.669484 | 1.14453 | -3.687234 | 447.1499 | 2.01775 | 447.1513 | 0.996 | 1.588501 |

Note: Coef.: Coefficient, Std. Err.: Standard Error, P>|z|: P-value for |z| statistic

**Supplementary Table 5F. Node-specific TNF-a**

| Side | Direct |  | Indirect |  | Difference |  |  | tau |
| --- | --- | --- | --- | --- | --- | --- | --- | --- |
|  | Coef. | Std. Err. | Coef. | Std. Err. | Coef. | Std. Err. | P>\|z\| |  |
| A B * | 0.5352474 | 1.208563 | -0.0001372 | 76.69987 | 0.5353846 | 76.70939 | 0.994 | 1.179581 |
| B C * | -0.5813228 | 1.204609 | -1.651713 | 634.2246 | 1.070391 | 634.2256 | 0.999 | 1.179556 |
| B D * | -1.211402 | 0.8552168 | -2.282092 | 446.6189 | 1.07069 | 446.6196 | 0.998 | 1.179557 |
| B E * | -3.456176 | 1.266225 | -4.523491 | 631.6118 | 1.067315 | 631.6127 | 0.999 | 1.179554 |
| B F * | -2.094754 | 0.8808798 | -3.165139 | 448.3798 | 1.070385 | 448.3805 | 0.998 | 1.179557 |
| B G * | -0.5063325 | 0.8537499 | -1.576614 | 446.2803 | 1.070281 | 446.2811 | 0.998 | 1.179557 |
| B H * | -0.8049684 | 0.8546878 | -1.875219 | 446.3789 | 1.07025 | 446.3797 | 0.998 | 1.179557 |
| B I * | -2.125118 | 0.8717344 | -3.195363 | 447.6593 | 1.070245 | 447.66 | 0.998 | 1.179557 |
| B J * | -7.652424 | 1.333883 | -8.722588 | 630.129 | 1.070163 | 630.1298 | 0.999 | 1.179556 |
| B K * | -0.5486026 | 0.8492704 | -1.61888 | 446.9732 | 1.070278 | 446.9739 | 0.998 | 1.179557 |
| B L * | -2.177252 | 1.225119 | -3.247241 | 632.8092 | 1.06999 | 632.8102 | 0.999 | 1.179557 |
| B M * | -0.3997904 | 1.208082 | -1.4607 | 633.7405 | 1.060909 | 633.7416 | 0.999 | 1.179555 |

**Note**: **Side**: treatment comparison; **Direct/Indirect Coef. & Std. Err**.: effect estimates and standard errors from direct or indirect comparisons; **Difference**: difference between direct and indirect estimates, with SE and p-value; **tau**: between-study heterogeneity.

**Supplementary Table 5G. Node-specific IL-6**

| Side | Direct |  | Indirect |  | Difference |  |  | tau |
| --- | --- | --- | --- | --- | --- | --- | --- | --- |
|  | Coef. | Std. Err. | Coef. | Std. Err. | Coef. | Std. Err. | P>\|z\| |  |
| A B * | 0.6 | 0.3 | 0.0 | 91.00008 | 0.5524898 | 91.00067 | 0.995 | 0.1919169 |
| B C * | -0.5 | 0.3 | -1.6 | 614.4375 | 1.081892 | 614.4375 | 0.999 | 0.1919154 |
| B D * | -4.2 | 0.6 | -5.4 | 622.1954 | 1.117564 | 622.1952 | 0.999 | 0.1919152 |
| B E * | -0.9 | 0.3 | -2.0 | 440.2502 | 1.124124 | 440.2501 | 0.998 | 0.1919153 |
| B F * | -0.5 | 0.3 | -1.7 | 635.5315 | 1.184979 | 635.5315 | 0.999 | 0.1919156 |
| B G * | -1.0 | 0.2 | -2.1 | 363.6909 | 1.095344 | 363.6909 | 0.998 | 0.1919159 |
| B H * | -3.6 | 0.5 | -4.7 | 599.5921 | 1.104967 | 599.592 | 0.999 | 0.1919156 |
| B I * | -3.4 | 0.4 | -4.5 | 607.8245 | 1.104954 | 607.8245 | 0.999 | 0.1919156 |
| B J * | -0.7 | 0.3 | -1.8 | 601.2954 | 1.101306 | 601.2954 | 0.999 | 0.1919155 |
| B K * | -3.2 | 0.4 | -4.3 | 619.2386 | 1.103638 | 619.2385 | 0.999 | 0.1919156 |

**Note**: **Side**: treatment comparison; **Direct/Indirect Coef. & Std. Err**.: effect estimates and standard errors from direct or indirect comparisons; **Difference**: difference between direct and indirect estimates, with SE and p-value; **tau**: between-study heterogeneity.

**Supplementary Table 5H. Node-specific IAP**

| Side | Direct |  | Indirect |  | Difference |  |  | tau |
| --- | --- | --- | --- | --- | --- | --- | --- | --- |
| Coef. | Std. Err. | Coef. | Std. Err. | Coef. | Std. Err. | P>\|z\| |  |  |
| A B * | 2.948308 | 1.988966 | -0.0000138 | 67.41762 | 2.948322 | 67.44695 | 0.965 | 3.435474 |
| B C * | -1.295741 | 2.490964 | -7.187697 | 446.9454 | 5.891955 | 446.9516 | 0.989 | 3.435365 |
| B D * | -1.79 | 3.473713 | -7.68172 | 632.4537 | 5.891721 | 632.4628 | 0.993 | 3.435363 |
| B E * | -1.99 | 3.453335 | -7.881721 | 632.6409 | 5.891721 | 632.6502 | 0.993 | 3.43536 |
| B F * | -2.9 | 3.50916 | -8.791722 | 632.5303 | 5.891723 | 632.5392 | 0.993 | 3.43536 |
| B G * | -2.05 | 3.553364 | -7.941727 | 632.345 | 5.891727 | 632.3537 | 0.993 | 3.43536 |
| B H * | -2.279305 | 1.228161 | -8.172657 | 223.6442 | 5.893353 | 223.6474 | 0.979 | 3.435398 |
| B I * | -2.84 | 3.528957 | -8.731716 | 632.379 | 5.891716 | 632.3878 | 0.993 | 3.43536 |
| B J * | -4.931693 | 2.122167 | -10.82388 | 365.163 | 5.892188 | 365.1676 | 0.987 | 3.435371 |
| B K * | -2.300001 | 3.548088 | -8.191688 | 632.5018 | 5.891687 | 632.5105 | 0.993 | 3.435362 |
| B L * | 0.9459137 | 2.467051 | -4.946047 | 447.3902 | 5.89196 | 447.3966 | 0.989 | 3.435365 |
| B M * | -3.28 | 3.461044 | -9.17175 | 632.5039 | 5.891749 | 632.5131 | 0.993 | 3.43536 |

**Note**: **Side**: treatment comparison; **Direct/Indirect Coef. & Std. Err**.: effect estimates and standard errors from direct or indirect comparisons; **Difference**: difference between direct and indirect estimates, with SE and p-value; **tau**: between-study heterogeneity.

**Supplementary Table 6A. League table of Gastrointestinal dysfunction score**

| Dachaihu Tang | Dahuang Gancao Tang | Dachengqi Tang | Rhubarb | Banxia Xiexin Tang | Yantiao Fang | Zengye Chengqi Tang | Yiqi Tang | Intestinal Comfort | Dahuang Fuzi Tang | Dahuang Mudan Tang | Tiaoqi Tongfu Tang | Xuebijing | Sijunzi Tang | Houpo Heji | Conventional Therapy |
| --- | --- | --- | --- | --- | --- | --- | --- | --- | --- | --- | --- | --- | --- | --- | --- |
| Dachaihu Tang | 1.00 (-0.91,2.91) | 1.12 (-0.82,3.05) | 1.19 (-0.66,3.05) | 1.27 (-0.60,3.13) | 1.37 (-0.73,3.47) | 1.40 (-0.63,3.43) | 1.45 (-0.46,3.36) | 1.49 (-0.47,3.44) | 1.53 (-0.32,3.39) | 1.59 (-0.45,3.63) | 1.59 (-0.36,3.54) | 1.72 (-0.31,3.75) | 1.70 (-0.24,3.65) | 1.78 (-0.16,3.72) | 2.03 (0.20,3.86) |
| -1.00 (-2.91,0.91) | Dahuang Gancao Tang | 0.12 (-0.69,0.93) | 0.20 (-0.40,0.80) | 0.27 (-0.35,0.88) | 0.37 (-0.78,1.52) | 0.40 (-0.62,1.42) | 0.45 (-0.29,1.20) | 0.49 (-0.36,1.34) | 0.53 (-0.06,1.13) | 0.59 (-0.45,1.63) | 0.59 (-0.25,1.44) | 0.72 (-0.30,1.74) | 0.71 (-0.12,1.53) | 0.78 (-0.04,1.60) | 1.03 (0.51,1.55) |
| -1.12 (-3.05,0.82) | -0.12 (-0.93,0.69) | Dachengqi Tang | 0.08 (-0.61,0.77) | 0.15 (-0.55,0.85) | 0.25 (-0.94,1.45) | 0.28 (-0.79,1.36) | 0.33 (-0.48,1.15) | 0.37 (-0.54,1.28) | 0.42 (-0.27,1.10) | 0.47 (-0.62,1.57) | 0.48 (-0.44,1.39) | 0.60 (-0.47,1.68) | 0.59 (-0.30,1.48) | 0.66 (-0.22,1.55) | 0.91 (0.29,1.54) |
| -1.19 (-3.05,0.66) | -0.20 (-0.80,0.40) | -0.08 (-0.77,0.61) | Rhubarb | 0.07 (-0.37,0.51) | 0.18 (-0.89,1.24) | 0.21 (-0.72,1.14) | 0.26 (-0.35,0.86) | 0.29 (-0.44,1.03) | 0.34 (-0.07,0.75) | 0.40 (-0.55,1.34) | 0.40 (-0.33,1.13) | 0.53 (-0.40,1.46) | 0.51 (-0.20,1.22) | 0.59 (-0.11,1.28) | 0.84 (0.54,1.13) |
| -1.27 (-3.13,0.60) | -0.27 (-0.88,0.35) | -0.15 (-0.85,0.55) | -0.07 (-0.51,0.37) | Banxia Xiexin Tang | 0.10 (-0.97,1.18) | 0.13 (-0.81,1.07) | 0.18 (-0.44,0.81) | 0.22 (-0.52,0.96) | 0.27 (-0.16,0.70) | 0.32 (-0.63,1.28) | 0.33 (-0.41,1.07) | 0.45 (-0.49,1.39) | 0.44 (-0.28,1.16) | 0.51 (-0.19,1.22) | 0.76 (0.44,1.09) |
| -1.37 (-3.47,0.73) | -0.37 (-1.52,0.78) | -0.25 (-1.45,0.94) | -0.18 (-1.24,0.89) | -0.10 (-1.18,0.97) | Yantiao Fang | 0.03 (-1.32,1.38) | 0.08 (-1.07,1.23) | 0.12 (-1.11,1.34) | 0.16 (-0.90,1.22) | 0.22 (-1.14,1.58) | 0.22 (-1.00,1.44) | 0.35 (-1.00,1.70) | 0.33 (-0.87,1.54) | 0.41 (-0.79,1.61) | 0.66 (-0.36,1.68) |
| -1.40 (-3.43,0.63) | -0.40 (-1.42,0.62) | -0.28 (-1.36,0.79) | -0.21 (-1.14,0.72) | -0.13 (-1.07,0.81) | -0.03 (-1.38,1.32) | Zengye Chengqi Tang | 0.05 (-0.98,1.08) | 0.09 (-1.02,1.19) | 0.13 (-0.79,1.06) | 0.19 (-1.07,1.45) | 0.19 (-0.91,1.30) | 0.32 (-0.93,1.57) | 0.30 (-0.78,1.39) | 0.38 (-0.70,1.46) | 0.63 (-0.25,1.51) |
| -1.45 (-3.36,0.46) | -0.45 (-1.20,0.29) | -0.33 (-1.15,0.48) | -0.26 (-0.86,0.35) | -0.18 (-0.81,0.44) | -0.08 (-1.23,1.07) | -0.05 (-1.08,0.98) | Yiqi Tang | 0.04 (-0.82,0.89) | 0.08 (-0.52,0.68) | 0.14 (-0.90,1.18) | 0.14 (-0.71,1.00) | 0.27 (-0.76,1.30) | 0.25 (-0.58,1.09) | 0.33 (-0.49,1.15) | 0.58 (0.05,1.11) |
| -1.49 (-3.44,0.47) | -0.49 (-1.34,0.36) | -0.37 (-1.28,0.54) | -0.29 (-1.03,0.44) | -0.22 (-0.96,0.52) | -0.12 (-1.34,1.11) | -0.09 (-1.19,1.02) | -0.04 (-0.89,0.82) | Intestinal Comfort | 0.05 (-0.68,0.77) | 0.10 (-1.02,1.22) | 0.11 (-0.84,1.05) | 0.23 (-0.87,1.34) | 0.22 (-0.71,1.15) | 0.29 (-0.63,1.21) | 0.54 (-0.13,1.21) |
| -1.53 (-3.39,0.32) | -0.53 (-1.13,0.06) | -0.42 (-1.10,0.27) | -0.34 (-0.75,0.07) | -0.27 (-0.70,0.16) | -0.16 (-1.22,0.90) | -0.13 (-1.06,0.79) | -0.08 (-0.68,0.52) | -0.05 (-0.77,0.68) | Dahuang Fuzi Tang | 0.06 (-0.88,1.00) | 0.06 (-0.66,0.78) | 0.19 (-0.74,1.11) | 0.17 (-0.53,0.87) | 0.25 (-0.44,0.93) | 0.50 (0.22,0.78) |
| -1.59 (-3.63,0.45) | -0.59 (-1.63,0.45) | -0.47 (-1.57,0.62) | -0.40 (-1.34,0.55) | -0.32 (-1.28,0.63) | -0.22 (-1.58,1.14) | -0.19 (-1.45,1.07) | -0.14 (-1.18,0.90) | -0.10 (-1.22,1.02) | -0.06 (-1.00,0.88) | Dahuang Mudan Tang | 0.00 (-1.12,1.12) | 0.13 (-1.13,1.39) | 0.11 (-0.99,1.22) | 0.19 (-0.91,1.29) | 0.44 (-0.46,1.34) |
| -1.59 (-3.54,0.36) | -0.59 (-1.44,0.25) | -0.48 (-1.39,0.44) | -0.40 (-1.13,0.33) | -0.33 (-1.07,0.41) | -0.22 (-1.44,1.00) | -0.19 (-1.30,0.91) | -0.14 (-1.00,0.71) | -0.11 (-1.05,0.84) | -0.06 (-0.78,0.66) | -0.00 (-1.12,1.12) | Tiaoqi Tongfu Tang | 0.13 (-0.98,1.23) | 0.11 (-0.81,1.04) | 0.19 (-0.73,1.10) | 0.44 (-0.23,1.10) |
| -1.72 (-3.75,0.31) | -0.72 (-1.74,0.30) | -0.60 (-1.68,0.47) | -0.53 (-1.46,0.40) | -0.45 (-1.39,0.49) | -0.35 (-1.70,1.00) | -0.32 (-1.57,0.93) | -0.27 (-1.30,0.76) | -0.23 (-1.34,0.87) | -0.19 (-1.11,0.74) | -0.13 (-1.39,1.13) | -0.13 (-1.23,0.98) | Xuebijing | -0.02 (-1.10,1.07) | 0.06 (-1.02,1.14) | 0.31 (-0.57,1.19) |
| -1.70 (-3.65,0.24) | -0.71 (-1.53,0.12) | -0.59 (-1.48,0.30) | -0.51 (-1.22,0.20) | -0.44 (-1.16,0.28) | -0.33 (-1.54,0.87) | -0.30 (-1.39,0.78) | -0.25 (-1.09,0.58) | -0.22 (-1.15,0.71) | -0.17 (-0.87,0.53) | -0.11 (-1.22,0.99) | -0.11 (-1.04,0.81) | 0.02 (-1.07,1.10) | Sijunzi Tang | 0.07 (-0.82,0.97) | 0.33 (-0.32,0.97) |
| -1.78 (-3.72,0.16) | -0.78 (-1.60,0.04) | -0.66 (-1.55,0.22) | -0.59 (-1.28,0.11) | -0.51 (-1.22,0.19) | -0.41 (-1.61,0.79) | -0.38 (-1.46,0.70) | -0.33 (-1.15,0.49) | -0.29 (-1.21,0.63) | -0.25 (-0.93,0.44) | -0.19 (-1.29,0.91) | -0.19 (-1.10,0.73) | -0.06 (-1.14,1.02) | -0.07 (-0.97,0.82) | Houpo Heji | 0.25 (-0.38,0.88) |
| -2.03 (-3.86,-0.20) | -1.03 (-1.55,-0.51) | -0.91 (-1.54,-0.29) | -0.84 (-1.13,-0.54) | -0.76 (-1.09,-0.44) | -0.66 (-1.68,0.36) | -0.63 (-1.51,0.25) | -0.58 (-1.11,-0.05) | -0.54 (-1.21,0.13) | -0.50 (-0.78,-0.22) | -0.44 (-1.34,0.46) | -0.44 (-1.10,0.23) | -0.31 (-1.19,0.57) | -0.33 (-0.97,0.32) | -0.25 (-0.88,0.38) | Conventional Therapy |

**Note:** League table showing pairwise comparisons of treatments for Gastrointestinal Dysfunction (GID) score. Values represent mean differences (MD) with 95% confidence intervals (CI). Positive values favor the treatment in the row, negative values favor the treatment in the column.

**Supplementary Table 6B. League table of TCM symptom score**

| Yiqi Tang | Dahuang Fuzi Tang | Dachaihu Tang | Zengye Chengqi Tang | Sijunzi Tang | Tiaoqi Tongfu Tang | Liangge San | Yantiao Fang | Banxia Xiexin Tang | Xuebijing | Conventional Therapy |
| --- | --- | --- | --- | --- | --- | --- | --- | --- | --- | --- |
| Yiqi Tang | 0.39 (-4.14,4.93) | 1.53 (-4.48,7.55) | 1.66 (-3.26,6.59) | 1.68 (-4.94,8.30) | 2.00 (-2.70,6.69) | 2.14 (-3.87,8.15) | 2.23 (-2.52,6.98) | 2.23 (-2.24,6.69) | 3.96 (-1.69,9.62) | 5.70 (1.56,9.84) |
| -0.39 (-4.93,4.14) | Dahuang Fuzi Tang | 1.14 (-3.72,6.00) | 1.27 (-2.16,4.71) | 1.29 (-4.30,6.88) | 1.60 (-1.51,4.72) | 1.75 (-3.11,6.61) | 1.84 (-1.36,5.03) | 1.83 (-1.03,4.70) | 3.57 (-0.84,7.97) | 5.31 (3.17,7.45) |
| -1.53 (-7.55,4.48) | -1.14 (-6.00,3.72) | Dachaihu Tang | 0.13 (-4.99,5.26) | 0.15 (-6.61,6.91) | 0.46 (-4.45,5.38) | 0.61 (-5.56,6.78) | 0.70 (-4.27,5.67) | 0.69 (-4.08,5.47) | 2.43 (-3.39,8.25) | 4.17 (-0.19,8.53) |
| -1.66 (-6.59,3.26) | -1.27 (-4.71,2.16) | -0.13 (-5.26,4.99) | Zengye Chengqi Tang | 0.02 (-5.81,5.84) | 0.33 (-3.19,3.85) | 0.48 (-4.65,5.60) | 0.57 (-3.03,4.16) | 0.56 (-2.75,3.88) | 2.30 (-2.40,7.00) | 4.04 (1.35,6.73) |
| -1.68 (-8.30,4.94) | -1.29 (-6.88,4.30) | -0.15 (-6.91,6.61) | -0.02 (-5.84,5.81) | Sijunzi Tang | 0.31 (-5.33,5.96) | 0.46 (-6.30,7.22) | 0.55 (-5.14,6.23) | 0.54 (-4.97,6.06) | 2.28 (-4.16,8.72) | 4.02 (-1.14,9.18) |
| -2.00 (-6.69,2.70) | -1.60 (-4.72,1.51) | -0.46 (-5.38,4.45) | -0.33 (-3.85,3.19) | -0.31 (-5.96,5.33) | Tiaoqi Tongfu Tang | 0.15 (-4.77,5.06) | 0.23 (-3.06,3.52) | 0.23 (-2.75,3.21) | 1.97 (-2.51,6.44) | 3.71 (1.43,5.98) |
| -2.14 (-8.15,3.87) | -1.75 (-6.61,3.11) | -0.61 (-6.78,5.56) | -0.48 (-5.60,4.65) | -0.46 (-7.22,6.30) | -0.15 (-5.06,4.77) | Liangge San | 0.09 (-4.88,5.05) | 0.08 (-4.69,4.85) | 1.82 (-4.00,7.64) | 3.56 (-0.80,7.92) |
| -2.23 (-6.98,2.52) | -1.84 (-5.03,1.36) | -0.70 (-5.67,4.27) | -0.57 (-4.16,3.03) | -0.55 (-6.23,5.14) | -0.23 (-3.52,3.06) | -0.09 (-5.05,4.88) | Yantiao Fang | -0.00 (-3.07,3.06) | 1.73 (-2.79,6.26) | 3.47 (1.09,5.85) |
| -2.23 (-6.69,2.24) | -1.83 (-4.70,1.03) | -0.69 (-5.47,4.08) | -0.56 (-3.88,2.75) | -0.54 (-6.06,4.97) | -0.23 (-3.21,2.75) | -0.08 (-4.85,4.69) | 0.00 (-3.06,3.07) | Banxia Xiexin Tang | 1.74 (-2.57,6.05) | 3.48 (1.54,5.41) |
| -3.96 (-9.62,1.69) | -3.57 (-7.97,0.84) | -2.43 (-8.25,3.39) | -2.30 (-7.00,2.40) | -2.28 (-8.72,4.16) | -1.97 (-6.44,2.51) | -1.82 (-7.64,4.00) | -1.73 (-6.26,2.79) | -1.74 (-6.05,2.57) | Xuebijing | 1.74 (-2.11,5.59) |
| -5.70 (-9.84,-1.56) | -5.31 (-7.45,-3.17) | -4.17 (-8.53,0.19) | -4.04 (-6.73,-1.35) | -4.02 (-9.18,1.14) | -3.71 (-5.98,-1.43) | -3.56 (-7.92,0.80) | -3.47 (-5.85,-1.09) | -3.48 (-5.41,-1.54) | -1.74 (-5.59,2.11) | Conventional Therapy |

**Note:** League table showing pairwise comparisons of treatments for Gastrointestinal Dysfunction (GID) score. Values represent mean differences (MD) with 95% confidence intervals (CI). Positive values favor the treatment in the row, negative values favor the treatment in the column.

**Supplementary Table 6C. League table of Apache Ⅱ score**

| Tiaoqi Tongfu Tang | Yiqi Tang | Rhubarb | Dachaihu Tang | Houpo Heji | Zengye Chengqi Tang | Dahuang Fuzi Tang | Dachengqi Tang | Dahuang Mudan Tang | Liangge San | Sijunzi Tang | Banxia Xiexin Tang | Intestinal Comfort | Yantiao Fang | Xuebijing | Conventional Therapy |
| --- | --- | --- | --- | --- | --- | --- | --- | --- | --- | --- | --- | --- | --- | --- | --- |
| Tiaoqi Tongfu Tang | 1.25 (-3.19,5.70) | 1.64 (-1.68,4.96) | 1.21 (-5.89,8.30) | 2.80 (-2.26,7.86) | 2.89 (-2.21,7.98) | 3.24 (-0.07,6.55) | 3.25 (-1.85,8.35) | 3.36 (-1.61,8.33) | 3.56 (-1.67,8.79) | 4.07 (-1.09,9.22) | 4.60 (1.01,8.20) | 5.08 (0.10,10.07) | 5.44 (0.40,10.48) | 5.71 (0.68,10.75) | 6.90 (4.13,9.66) |
| -1.25 (-5.70,3.19) | Yiqi Tang | 0.38 (-3.55,4.32) | -0.05 (-7.45,7.35) | 1.55 (-3.93,7.03) | 1.63 (-3.88,7.15) | 1.99 (-1.94,5.91) | 2.00 (-3.52,7.52) | 2.11 (-3.29,7.51) | 2.31 (-3.33,7.95) | 2.81 (-2.76,8.38) | 3.35 (-0.82,7.52) | 3.83 (-1.58,9.24) | 4.19 (-1.28,9.65) | 4.46 (-1.00,9.92) | 5.64 (2.16,9.12) |
| -1.64 (-4.96,1.68) | -0.38 (-4.32,3.55) | Rhubarb | -0.43 (-7.21,6.35) | 1.16 (-3.45,5.78) | 1.25 (-3.41,5.90) | 1.60 (-0.97,4.18) | 1.61 (-3.05,6.27) | 1.72 (-2.79,6.24) | 1.92 (-2.88,6.73) | 2.43 (-2.29,7.15) | 2.97 (0.03,5.90) | 3.45 (-1.08,7.98) | 3.81 (-0.79,8.40) | 4.08 (-0.51,8.67) | 5.26 (3.43,7.09) |
| -1.21 (-8.30,5.89) | 0.05 (-7.35,7.45) | 0.43 (-6.35,7.21) | Dachaihu Tang | 1.60 (-6.19,9.38) | 1.68 (-6.13,9.49) | 2.03 (-4.75,8.82) | 2.04 (-5.77,9.86) | 2.15 (-5.57,9.88) | 2.36 (-5.54,10.25) | 2.86 (-4.99,10.71) | 3.40 (-3.53,10.32) | 3.88 (-3.86,11.61) | 4.24 (-3.54,12.01) | 4.51 (-3.26,12.28) | 5.69 (-0.84,12.22) |
| -2.80 (-7.86,2.26) | -1.55 (-7.03,3.93) | -1.16 (-5.78,3.45) | -1.60 (-9.38,6.19) | Houpo Heji | 0.09 (-5.94,6.11) | 0.44 (-4.17,5.05) | 0.45 (-5.58,6.47) | 0.56 (-5.36,6.47) | 0.76 (-5.37,6.90) | 1.27 (-4.81,7.34) | 1.80 (-3.02,6.62) | 2.28 (-3.64,8.21) | 2.64 (-3.33,8.62) | 2.91 (-3.06,8.88) | 4.10 (-0.14,8.33) |
| -2.89 (-7.98,2.21) | -1.63 (-7.15,3.88) | -1.25 (-5.90,3.41) | -1.68 (-9.49,6.13) | -0.09 (-6.11,5.94) | Zengye Chengqi Tang | 0.35 (-4.30,5.00) | 0.36 (-5.69,6.42) | 0.47 (-5.47,6.42) | 0.68 (-5.49,6.84) | 1.18 (-4.92,7.28) | 1.72 (-3.14,6.58) | 2.20 (-3.76,8.16) | 2.56 (-3.45,8.56) | 2.83 (-3.18,8.83) | 4.01 (-0.27,8.29) |
| -3.24 (-6.55,0.07) | -1.99 (-5.91,1.94) | -1.60 (-4.18,0.97) | -2.03 (-8.82,4.75) | -0.44 (-5.05,4.17) | -0.35 (-5.00,4.30) | Dahuang Fuzi Tang | 0.01 (-4.64,4.66) | 0.12 (-4.39,4.63) | 0.32 (-4.48,5.12) | 0.83 (-3.89,5.54) | 1.36 (-1.57,4.29) | 1.84 (-2.68,6.37) | 2.20 (-2.39,6.79) | 2.47 (-2.11,7.06) | 3.66 (1.84,5.47) |
| -3.25 (-8.35,1.85) | -2.00 (-7.52,3.52) | -1.61 (-6.27,3.05) | -2.04 (-9.86,5.77) | -0.45 (-6.47,5.58) | -0.36 (-6.42,5.69) | -0.01 (-4.66,4.64) | Dachengqi Tang | 0.11 (-5.84,6.06) | 0.31 (-5.86,6.48) | 0.82 (-5.29,6.92) | 1.35 (-3.51,6.22) | 1.83 (-4.13,7.80) | 2.19 (-3.82,8.20) | 2.46 (-3.54,8.47) | 3.65 (-0.64,7.93) |
| -3.36 (-8.33,1.61) | -2.11 (-7.51,3.29) | -1.72 (-6.24,2.79) | -2.15 (-9.88,5.57) | -0.56 (-6.47,5.36) | -0.47 (-6.42,5.47) | -0.12 (-4.63,4.39) | -0.11 (-6.06,5.84) | Dahuang Mudan Tang | 0.20 (-5.86,6.26) | 0.71 (-5.29,6.71) | 1.24 (-3.48,5.97) | 1.72 (-4.13,7.58) | 2.08 (-3.82,7.98) | 2.35 (-3.54,8.25) | 3.54 (-0.59,7.67) |
| -3.56 (-8.79,1.67) | -2.31 (-7.95,3.33) | -1.92 (-6.73,2.88) | -2.36 (-10.25,5.54) | -0.76 (-6.90,5.37) | -0.68 (-6.84,5.49) | -0.32 (-5.12,4.48) | -0.31 (-6.48,5.86) | -0.20 (-6.26,5.86) | Liangge San | 0.51 (-5.71,6.72) | 1.04 (-3.96,6.04) | 1.52 (-4.55,7.60) | 1.88 (-4.24,8.00) | 2.15 (-3.97,8.27) | 3.33 (-1.10,7.77) |
| -4.07 (-9.22,1.09) | -2.81 (-8.38,2.76) | -2.43 (-7.15,2.29) | -2.86 (-10.71,4.99) | -1.27 (-7.34,4.81) | -1.18 (-7.28,4.92) | -0.83 (-5.54,3.89) | -0.82 (-6.92,5.29) | -0.71 (-6.71,5.29) | -0.51 (-6.72,5.71) | Sijunzi Tang | 0.54 (-4.38,5.46) | 1.02 (-4.99,7.03) | 1.38 (-4.68,7.43) | 1.65 (-4.41,7.70) | 2.83 (-1.52,7.18) |
| -4.60 (-8.20,-1.01) | -3.35 (-7.52,0.82) | -2.97 (-5.90,-0.03) | -3.40 (-10.32,3.53) | -1.80 (-6.62,3.02) | -1.72 (-6.58,3.14) | -1.36 (-4.29,1.57) | -1.35 (-6.22,3.51) | -1.24 (-5.97,3.48) | -1.04 (-6.04,3.96) | -0.54 (-5.46,4.38) | Banxia Xiexin Tang | 0.48 (-4.26,5.22) | 0.84 (-3.96,5.64) | 1.11 (-3.69,5.91) | 2.29 (-0.01,4.59) |
| -5.08 (-10.07,-0.10) | -3.83 (-9.24,1.58) | -3.45 (-7.98,1.08) | -3.88 (-11.61,3.86) | -2.28 (-8.21,3.64) | -2.20 (-8.16,3.76) | -1.84 (-6.37,2.68) | -1.83 (-7.80,4.13) | -1.72 (-7.58,4.13) | -1.52 (-7.60,4.55) | -1.02 (-7.03,4.99) | -0.48 (-5.22,4.26) | Intestinal Comfort | 0.36 (-5.55,6.27) | 0.63 (-5.28,6.54) | 1.81 (-2.33,5.96) |
| -5.44 (-10.48,-0.40) | -4.19 (-9.65,1.28) | -3.81 (-8.40,0.79) | -4.24 (-12.01,3.54) | -2.64 (-8.62,3.33) | -2.56 (-8.56,3.45) | -2.20 (-6.79,2.39) | -2.19 (-8.20,3.82) | -2.08 (-7.98,3.82) | -1.88 (-8.00,4.24) | -1.38 (-7.43,4.68) | -0.84 (-5.64,3.96) | -0.36 (-6.27,5.55) | Yantiao Fang | 0.27 (-5.69,6.23) | 1.45 (-2.76,5.67) |
| -5.71 (-10.75,-0.68) | -4.46 (-9.92,1.00) | -4.08 (-8.67,0.51) | -4.51 (-12.28,3.26) | -2.91 (-8.88,3.06) | -2.83 (-8.83,3.18) | -2.47 (-7.06,2.11) | -2.46 (-8.47,3.54) | -2.35 (-8.25,3.54) | -2.15 (-8.27,3.97) | -1.65 (-7.70,4.41) | -1.11 (-5.91,3.69) | -0.63 (-6.54,5.28) | -0.27 (-6.23,5.69) | Xuebijing | 1.18 (-3.03,5.39) |
| -6.90 (-9.66,-4.13) | -5.64 (-9.12,-2.16) | -5.26 (-7.09,-3.43) | -5.69 (-12.22,0.84) | -4.10 (-8.33,0.14) | -4.01 (-8.29,0.27) | -3.66 (-5.47,-1.84) | -3.65 (-7.93,0.64) | -3.54 (-7.67,0.59) | -3.33 (-7.77,1.10) | -2.83 (-7.18,1.52) | -2.29 (-4.59,0.01) | -1.81 (-5.96,2.33) | -1.45 (-5.67,2.76) | -1.18 (-5.39,3.03) | Conventional Therapy |

**Note:** League table showing pairwise comparisons of treatments for Gastrointestinal Dysfunction (GID) score. Values represent mean differences (MD) with 95% confidence intervals (CI). Positive values favor the treatment in the row, negative values favor the treatment in the column.

**Supplementary Table 6D. League table of AGI classification**

| Dahuang Fuzi Tang | Zengye Chengqi Tang | Rhubarb | Conventional Therapy |
| --- | --- | --- | --- |
| Dahuang Fuzi Tang | 1.59 (0.60,4.24) | 2.05 (0.89,4.74) | 3.65 (2.51,5.30) |
| 0.63 (0.24,1.68) | Zengye Chengqi Tang | 1.29 (0.40,4.18) | 2.30 (0.93,5.69) |
| 0.49 (0.21,1.13) | 0.78 (0.24,2.52) | Rhubarb | 1.78 (0.84,3.77) |
| 0.27 (0.19,0.40) | 0.44 (0.18,1.08) | 0.56 (0.26,1.19) | Conventional Therapy |

**Note:** League table showing pairwise comparisons of treatments for Gastrointestinal Dysfunction (GID) score. Values represent mean differences (MD) with 95% confidence intervals (CI). Positive values favor the treatment in the row, negative values favor the treatment in the column.

**Supplementary Table 6E. League table of D-lactic acid**

| Sijunzi Tang | Banxia Xiexin Tang | Tiaoqi Tongfu Tang | Intestinal Comfort | Yiqi Tang | Rhubarb | Zengye Chengqi Tang | Dahuang Mudan Tang | Houpo Heji | Yantiao Fang | Liangge San | Dahuang Fuzi Tang | Conventional Therapy |
| --- | --- | --- | --- | --- | --- | --- | --- | --- | --- | --- | --- | --- |
| Sijunzi Tang | 0.37 (-3.56,4.31) | 0.92 (-3.59,5.44) | 1.08 (-2.86,5.03) | 1.67 (-2.83,6.17) | 1.91 (-2.59,6.41) | 2.00 (-1.92,5.92) | 2.17 (-1.74,6.09) | 2.69 (-1.82,7.21) | 2.64 (-1.27,6.55) | 2.86 (-1.64,7.36) | 2.98 (-1.52,7.49) | 3.51 (0.29,6.73) |
| -0.37 (-4.31,3.56) | Banxia Xiexin Tang | 0.55 (-3.34,4.44) | 0.71 (-2.50,3.92) | 1.30 (-2.57,5.17) | 1.54 (-2.34,5.41) | 1.63 (-1.56,4.81) | 1.80 (-1.37,4.98) | 2.32 (-1.57,6.21) | 2.27 (-0.90,5.44) | 2.49 (-1.38,6.36) | 2.61 (-1.27,6.49) | 3.14 (0.88,5.40) |
| -0.92 (-5.44,3.59) | -0.55 (-4.44,3.34) | Tiaoqi Tongfu Tang | 0.16 (-3.74,4.06) | 0.75 (-3.71,5.21) | 0.99 (-3.47,5.45) | 1.07 (-2.80,4.95) | 1.25 (-2.62,5.12) | 1.77 (-2.70,6.24) | 1.72 (-2.15,5.58) | 1.94 (-2.52,6.39) | 2.06 (-2.41,6.53) | 2.59 (-0.57,5.75) |
| -1.08 (-5.03,2.86) | -0.71 (-3.92,2.50) | -0.16 (-4.06,3.74) | Intestinal Comfort | 0.59 (-3.30,4.47) | 0.83 (-3.06,4.71) | 0.91 (-2.28,4.11) | 1.09 (-2.10,4.27) | 1.61 (-2.29,5.51) | 1.56 (-1.63,4.74) | 1.77 (-2.11,5.66) | 1.90 (-1.99,5.79) | 2.43 (0.15,4.71) |
| -1.67 (-6.17,2.83) | -1.30 (-5.17,2.57) | -0.75 (-5.21,3.71) | -0.59 (-4.47,3.30) | Yiqi Tang | 0.24 (-4.21,4.68) | 0.32 (-3.53,4.18) | 0.50 (-3.35,4.35) | 1.02 (-3.44,5.48) | 0.97 (-2.88,4.82) | 1.19 (-3.26,5.63) | 1.31 (-3.14,5.76) | 1.84 (-1.30,4.98) |
| -1.91 (-6.41,2.59) | -1.54 (-5.41,2.34) | -0.99 (-5.45,3.47) | -0.83 (-4.71,3.06) | -0.24 (-4.68,4.21) | Rhubarb | 0.09 (-3.77,3.94) | 0.26 (-3.59,4.11) | 0.78 (-3.68,5.24) | 0.73 (-3.12,4.58) | 0.95 (-3.50,5.39) | 1.07 (-3.38,5.52) | 1.60 (-1.54,4.75) |
| -2.00 (-5.92,1.92) | -1.63 (-4.81,1.56) | -1.07 (-4.95,2.80) | -0.91 (-4.11,2.28) | -0.32 (-4.18,3.53) | -0.09 (-3.94,3.77) | Zengye Chengqi Tang | 0.18 (-2.98,3.33) | 0.70 (-3.18,4.57) | 0.64 (-2.51,3.80) | 0.86 (-2.99,4.72) | 0.99 (-2.88,4.85) | 1.51 (-0.72,3.75) |
| -2.17 (-6.09,1.74) | -1.80 (-4.98,1.37) | -1.25 (-5.12,2.62) | -1.09 (-4.27,2.10) | -0.50 (-4.35,3.35) | -0.26 (-4.11,3.59) | -0.18 (-3.33,2.98) | Dahuang Mudan Tang | 0.52 (-3.35,4.39) | 0.47 (-2.68,3.61) | 0.69 (-3.16,4.54) | 0.81 (-3.05,4.67) | 1.34 (-0.89,3.57) |
| -2.69 (-7.21,1.82) | -2.32 (-6.21,1.57) | -1.77 (-6.24,2.70) | -1.61 (-5.51,2.29) | -1.02 (-5.48,3.44) | -0.78 (-5.24,3.68) | -0.70 (-4.57,3.18) | -0.52 (-4.39,3.35) | Houpo Heji | -0.05 (-3.92,3.81) | 0.17 (-4.29,4.62) | 0.29 (-4.18,4.75) | 0.82 (-2.34,3.98) |
| -2.64 (-6.55,1.27) | -2.27 (-5.44,0.90) | -1.72 (-5.58,2.15) | -1.56 (-4.74,1.63) | -0.97 (-4.82,2.88) | -0.73 (-4.58,3.12) | -0.64 (-3.80,2.51) | -0.47 (-3.61,2.68) | 0.05 (-3.81,3.92) | Yantiao Fang | 0.22 (-3.63,4.07) | 0.34 (-3.51,4.20) | 0.87 (-1.35,3.09) |
| -2.86 (-7.36,1.64) | -2.49 (-6.36,1.38) | -1.94 (-6.39,2.52) | -1.77 (-5.66,2.11) | -1.19 (-5.63,3.26) | -0.95 (-5.39,3.50) | -0.86 (-4.72,2.99) | -0.69 (-4.54,3.16) | -0.17 (-4.62,4.29) | -0.22 (-4.07,3.63) | Liangge San | 0.12 (-4.33,4.57) | 0.65 (-2.49,3.79) |
| -2.98 (-7.49,1.52) | -2.61 (-6.49,1.27) | -2.06 (-6.53,2.41) | -1.90 (-5.79,1.99) | -1.31 (-5.76,3.14) | -1.07 (-5.52,3.38) | -0.99 (-4.85,2.88) | -0.81 (-4.67,3.05) | -0.29 (-4.75,4.18) | -0.34 (-4.20,3.51) | -0.12 (-4.57,4.33) | Dahuang Fuzi Tang | 0.53 (-2.62,3.68) |
| -3.51 (-6.73,-0.29) | -3.14 (-5.40,-0.88) | -2.59 (-5.75,0.57) | -2.43 (-4.71,-0.15) | -1.84 (-4.98,1.30) | -1.60 (-4.75,1.54) | -1.51 (-3.75,0.72) | -1.34 (-3.57,0.89) | -0.82 (-3.98,2.34) | -0.87 (-3.09,1.35) | -0.65 (-3.79,2.49) | -0.53 (-3.68,2.62) | Conventional Therapy |

**Note:** League table showing pairwise comparisons of treatments for Gastrointestinal Dysfunction (GID) score. Values represent mean differences (MD) with 95% confidence intervals (CI). Positive values favor the treatment in the row, negative values favor the treatment in the column.

**Supplementary Table 6F. League table of DAO mean**

| Xuebijing | Houpo Heji | Tiaoqi Tongfu Tang | Intestinal Comfort | Sijunzi Tang | Zengye Chengqi Tang | Liangge San | Rhubarb | Banxia Xiexin Tang | Yantiao Fang | Conventional Therapy |
| --- | --- | --- | --- | --- | --- | --- | --- | --- | --- | --- |
| Xuebijing | 1.72 (-2.88,6.32) | 2.27 (-2.29,6.84) | 2.42 (-1.58,6.42) | 2.73 (-1.85,7.30) | 2.90 (-1.08,6.88) | 3.07 (-1.49,7.63) | 3.25 (-1.30,7.80) | 3.56 (-0.42,7.54) | 3.83 (-0.73,8.39) | 4.57 (1.28,7.86) |
| -1.72 (-6.32,2.88) | Houpo Heji | 0.55 (-3.96,5.06) | 0.70 (-3.24,4.63) | 1.01 (-3.51,5.52) | 1.18 (-2.74,5.10) | 1.35 (-3.15,5.85) | 1.53 (-2.97,6.03) | 1.84 (-2.07,5.75) | 2.11 (-2.39,6.62) | 2.85 (-0.37,6.06) |
| -2.27 (-6.84,2.29) | -0.55 (-5.06,3.96) | Tiaoqi Tongfu Tang | 0.14 (-3.75,4.04) | 0.45 (-4.02,4.93) | 0.63 (-3.25,4.50) | 0.80 (-3.67,5.26) | 0.98 (-3.48,5.44) | 1.29 (-2.58,5.16) | 1.56 (-2.91,6.03) | 2.30 (-0.86,5.46) |
| -2.42 (-6.42,1.58) | -0.70 (-4.63,3.24) | -0.14 (-4.04,3.75) | Intestinal Comfort | 0.31 (-3.59,4.21) | 0.48 (-2.71,3.68) | 0.66 (-3.23,4.54) | 0.83 (-3.05,4.71) | 1.14 (-2.04,4.33) | 1.42 (-2.47,5.31) | 2.15 (-0.12,4.42) |
| -2.73 (-7.30,1.85) | -1.01 (-5.52,3.51) | -0.45 (-4.93,4.02) | -0.31 (-4.21,3.59) | Sijunzi Tang | 0.17 (-3.71,4.06) | 0.34 (-4.13,4.82) | 0.52 (-3.95,4.99) | 0.83 (-3.05,4.71) | 1.11 (-3.37,5.58) | 1.84 (-1.33,5.02) |
| -2.90 (-6.88,1.08) | -1.18 (-5.10,2.74) | -0.63 (-4.50,3.25) | -0.48 (-3.68,2.71) | -0.17 (-4.06,3.71) | Zengye Chengqi Tang | 0.17 (-3.70,4.04) | 0.35 (-3.51,4.21) | 0.66 (-2.50,3.82) | 0.93 (-2.94,4.81) | 1.67 (-0.57,3.91) |
| -3.07 (-7.63,1.49) | -1.35 (-5.85,3.15) | -0.80 (-5.26,3.67) | -0.66 (-4.54,3.23) | -0.34 (-4.82,4.13) | -0.17 (-4.04,3.70) | Liangge San | 0.18 (-4.27,4.63) | 0.49 (-3.37,4.35) | 0.76 (-3.70,5.22) | 1.50 (-1.65,4.65) |
| -3.25 (-7.80,1.30) | -1.53 (-6.03,2.97) | -0.98 (-5.44,3.48) | -0.83 (-4.71,3.05) | -0.52 (-4.99,3.95) | -0.35 (-4.21,3.51) | -0.18 (-4.63,4.27) | Rhubarb | 0.31 (-3.54,4.17) | 0.58 (-3.87,5.04) | 1.32 (-1.82,4.47) |
| -3.56 (-7.54,0.42) | -1.84 (-5.75,2.07) | -1.29 (-5.16,2.58) | -1.14 (-4.33,2.04) | -0.83 (-4.71,3.05) | -0.66 (-3.82,2.50) | -0.49 (-4.35,3.37) | -0.31 (-4.17,3.54) | Banxia Xiexin Tang | 0.27 (-3.59,4.14) | 1.01 (-1.22,3.24) |
| -3.83 (-8.39,0.73) | -2.11 (-6.62,2.39) | -1.56 (-6.03,2.91) | -1.42 (-5.31,2.47) | -1.11 (-5.58,3.37) | -0.93 (-4.81,2.94) | -0.76 (-5.22,3.70) | -0.58 (-5.04,3.87) | -0.27 (-4.14,3.59) | Yantiao Fang | 0.74 (-2.42,3.89) |
| -4.57 (-7.86,-1.28) | -2.85 (-6.06,0.37) | -2.30 (-5.46,0.86) | -2.15 (-4.42,0.12) | -1.84 (-5.02,1.33) | -1.67 (-3.91,0.57) | -1.50 (-4.65,1.65) | -1.32 (-4.47,1.82) | -1.01 (-3.24,1.22) | -0.74 (-3.89,2.42) | Conventional Therapy |

**Note:** League table showing pairwise comparisons of treatments for Gastrointestinal Dysfunction (GID) score. Values represent mean differences (MD) with 95% confidence intervals (CI). Positive values favor the treatment in the row, negative values favor the treatment in the column.

**Supplementary Table 6G. League table of TNF-a**

| Tiaoqi Tongfu Tang | Houpo Heji | Sijunzi Tang | Intestinal Comfort | Yiqi Tang | Dahuang Mudan Tang | Rhubarb | Dahuang Fuzi Tang | Banxia Xiexin Tang | Yantiao Fang | Liangge San | Zengye Chengqi Tang | Conventional Therapy |
| --- | --- | --- | --- | --- | --- | --- | --- | --- | --- | --- | --- | --- |
| Tiaoqi Tongfu Tang | 4.20 (0.59,7.80) | 5.53 (2.40,8.65) | 5.56 (2.42,8.69) | 5.48 (1.93,9.02) | 6.44 (3.34,9.55) | 6.85 (3.74,9.95) | 7.07 (3.55,10.59) | 7.12 (3.59,10.64) | 7.10 (4.00,10.20) | 7.15 (4.04,10.25) | 7.25 (3.73,10.78) | 7.65 (5.04,10.27) |
| -4.20 (-7.80,-0.59) | Houpo Heji | 1.33 (-1.68,4.34) | 1.36 (-1.66,4.38) | 1.28 (-2.17,4.73) | 2.24 (-0.75,5.24) | 2.65 (-0.34,5.65) | 2.87 (-0.55,6.30) | 2.92 (-0.51,6.35) | 2.91 (-0.08,5.90) | 2.95 (-0.04,5.94) | 3.06 (-0.37,6.49) | 3.46 (0.97,5.94) |
| -5.53 (-8.65,-2.40) | -1.33 (-4.34,1.68) | Sijunzi Tang | 0.03 (-2.40,2.46) | -0.05 (-3.00,2.89) | 0.91 (-1.48,3.31) | 1.32 (-1.07,3.71) | 1.54 (-1.37,4.46) | 1.59 (-1.33,4.51) | 1.58 (-0.81,3.96) | 1.62 (-0.77,4.01) | 1.73 (-1.19,4.65) | 2.13 (0.42,3.83) |
| -5.56 (-8.69,-2.42) | -1.36 (-4.38,1.66) | -0.03 (-2.46,2.40) | Intestinal Comfort | -0.08 (-3.04,2.87) | 0.88 (-1.52,3.29) | 1.29 (-1.12,3.70) | 1.51 (-1.41,4.44) | 1.56 (-1.37,4.49) | 1.55 (-0.85,3.94) | 1.59 (-0.82,3.99) | 1.69 (-1.24,4.63) | 2.09 (0.37,3.82) |
| -5.48 (-9.02,-1.93) | -1.28 (-4.73,2.17) | 0.05 (-2.89,3.00) | 0.08 (-2.87,3.04) | Yiqi Tang | 0.97 (-1.96,3.89) | 1.37 (-1.56,4.30) | 1.60 (-1.77,4.96) | 1.64 (-1.73,5.01) | 1.63 (-1.29,4.55) | 1.67 (-1.26,4.60) | 1.78 (-1.59,5.15) | 2.18 (-0.22,4.58) |
| -6.44 (-9.55,-3.34) | -2.24 (-5.24,0.75) | -0.91 (-3.31,1.48) | -0.88 (-3.29,1.52) | -0.97 (-3.89,1.96) | Dahuang Mudan Tang | 0.41 (-1.96,2.78) | 0.63 (-2.27,3.53) | 0.68 (-2.23,3.58) | 0.66 (-1.70,3.03) | 0.71 (-1.66,3.07) | 0.81 (-2.09,3.71) | 1.21 (-0.46,2.89) |
| -6.85 (-9.95,-3.74) | -2.65 (-5.65,0.34) | -1.32 (-3.71,1.07) | -1.29 (-3.70,1.12) | -1.37 (-4.30,1.56) | -0.41 (-2.78,1.96) | Rhubarb | 0.22 (-2.67,3.12) | 0.27 (-2.63,3.17) | 0.26 (-2.11,2.62) | 0.30 (-2.07,2.67) | 0.41 (-2.50,3.31) | 0.80 (-0.87,2.48) |
| -7.07 (-10.59,-3.55) | -2.87 (-6.30,0.55) | -1.54 (-4.46,1.37) | -1.51 (-4.44,1.41) | -1.60 (-4.96,1.77) | -0.63 (-3.53,2.27) | -0.22 (-3.12,2.67) | Dahuang Fuzi Tang | 0.05 (-3.30,3.39) | 0.03 (-2.86,2.92) | 0.07 (-2.82,2.97) | 0.18 (-3.16,3.53) | 0.58 (-1.78,2.94) |
| -7.12 (-10.64,-3.59) | -2.92 (-6.35,0.51) | -1.59 (-4.51,1.33) | -1.56 (-4.49,1.37) | -1.64 (-5.01,1.73) | -0.68 (-3.58,2.23) | -0.27 (-3.17,2.63) | -0.05 (-3.39,3.30) | Banxia Xiexin Tang | -0.01 (-2.91,2.88) | 0.03 (-2.87,2.93) | 0.14 (-3.21,3.48) | 0.54 (-1.83,2.90) |
| -7.10 (-10.20,-4.00) | -2.91 (-5.90,0.08) | -1.58 (-3.96,0.81) | -1.55 (-3.94,0.85) | -1.63 (-4.55,1.29) | -0.66 (-3.03,1.70) | -0.26 (-2.62,2.11) | -0.03 (-2.92,2.86) | 0.01 (-2.88,2.91) | Yantiao Fang | 0.04 (-2.32,2.40) | 0.15 (-2.75,3.04) | 0.55 (-1.12,2.21) |
| -7.15 (-10.25,-4.04) | -2.95 (-5.94,0.04) | -1.62 (-4.01,0.77) | -1.59 (-3.99,0.82) | -1.67 (-4.60,1.26) | -0.71 (-3.07,1.66) | -0.30 (-2.67,2.07) | -0.07 (-2.97,2.82) | -0.03 (-2.93,2.87) | -0.04 (-2.40,2.32) | Liangge San | 0.11 (-2.79,3.01) | 0.51 (-1.17,2.18) |
| -7.25 (-10.78,-3.73) | -3.06 (-6.49,0.37) | -1.73 (-4.65,1.19) | -1.69 (-4.63,1.24) | -1.78 (-5.15,1.59) | -0.81 (-3.71,2.09) | -0.41 (-3.31,2.50) | -0.18 (-3.53,3.16) | -0.14 (-3.48,3.21) | -0.15 (-3.04,2.75) | -0.11 (-3.01,2.79) | Zengye Chengqi Tang | 0.40 (-1.97,2.77) |
| -7.65 (-10.27,-5.04) | -3.46 (-5.94,-0.97) | -2.13 (-3.83,-0.42) | -2.09 (-3.82,-0.37) | -2.18 (-4.58,0.22) | -1.21 (-2.89,0.46) | -0.80 (-2.48,0.87) | -0.58 (-2.94,1.78) | -0.54 (-2.90,1.83) | -0.55 (-2.21,1.12) | -0.51 (-2.18,1.17) | -0.40 (-2.77,1.97) | Conventional Therapy |

**Note:** League table showing pairwise comparisons of treatments for Gastrointestinal Dysfunction (GID) score. Values represent mean differences (MD) with 95% confidence intervals (CI). Positive values favor the treatment in the row, negative values favor the treatment in the column.

**Supplementary Table 6H. League table of IL-6**

| Houpo Heji | Sijunzi Tang | Tiaoqi Tongfu Tang | Yiqi Tang | Rhubarb | Intestinal Comfort | Yantiao Fang | Banxia Xiexin Tang | Liangge San | Dahuang Fuzi Tang | Conventional Therapy |
| --- | --- | --- | --- | --- | --- | --- | --- | --- | --- | --- |
| Houpo Heji | 0.61 (-0.83,2.05) | 0.87 (-0.47,2.20) | 1.05 (-0.36,2.45) | 3.20 (2.05,4.36) | 3.38 (2.18,4.59) | 3.56 (2.29,4.84) | 3.68 (2.41,4.95) | 3.70 (2.46,4.94) | 3.76 (2.51,5.02) | 4.24 (3.14,5.34) |
| -0.61 (-2.05,0.83) | Sijunzi Tang | 0.26 (-0.94,1.45) | 0.44 (-0.83,1.70) | 2.60 (1.60,3.59) | 2.77 (1.73,3.82) | 2.96 (1.83,4.08) | 3.07 (1.95,4.20) | 3.09 (2.00,4.18) | 3.16 (2.05,4.26) | 3.63 (2.70,4.55) |
| -0.87 (-2.20,0.47) | -0.26 (-1.45,0.94) | Tiaoqi Tongfu Tang | 0.18 (-0.97,1.33) | 2.34 (1.50,3.17) | 2.52 (1.62,3.42) | 2.70 (1.71,3.69) | 2.82 (1.83,3.81) | 2.84 (1.89,3.79) | 2.90 (1.93,3.86) | 3.37 (2.62,4.12) |
| -1.05 (-2.45,0.36) | -0.44 (-1.70,0.83) | -0.18 (-1.33,0.97) | Yiqi Tang | 2.16 (1.22,3.10) | 2.34 (1.34,3.33) | 2.52 (1.44,3.59) | 2.64 (1.56,3.71) | 2.66 (1.61,3.70) | 2.72 (1.66,3.78) | 3.19 (2.32,4.06) |
| -3.20 (-4.36,-2.05) | -2.60 (-3.59,-1.60) | -2.34 (-3.17,-1.50) | -2.16 (-3.10,-1.22) | Rhubarb | 0.18 (-0.43,0.79) | 0.36 (-0.37,1.09) | 0.48 (-0.25,1.21) | 0.50 (-0.18,1.18) | 0.56 (-0.14,1.26) | 1.03 (0.67,1.39) |
| -3.38 (-4.59,-2.18) | -2.77 (-3.82,-1.73) | -2.52 (-3.42,-1.62) | -2.34 (-3.33,-1.34) | -0.18 (-0.79,0.43) | Intestinal Comfort | 0.18 (-0.62,0.99) | 0.30 (-0.50,1.11) | 0.32 (-0.44,1.08) | 0.38 (-0.40,1.16) | 0.85 (0.36,1.35) |
| -3.56 (-4.84,-2.29) | -2.96 (-4.08,-1.83) | -2.70 (-3.69,-1.71) | -2.52 (-3.59,-1.44) | -0.36 (-1.09,0.37) | -0.18 (-0.99,0.62) | Yantiao Fang | 0.12 (-0.78,1.02) | 0.14 (-0.72,1.00) | 0.20 (-0.68,1.08) | 0.67 (0.03,1.31) |
| -3.68 (-4.95,-2.41) | -3.07 (-4.20,-1.95) | -2.82 (-3.81,-1.83) | -2.64 (-3.71,-1.56) | -0.48 (-1.21,0.25) | -0.30 (-1.11,0.50) | -0.12 (-1.02,0.78) | Banxia Xiexin Tang | 0.02 (-0.84,0.88) | 0.08 (-0.80,0.96) | 0.55 (-0.09,1.19) |
| -3.70 (-4.94,-2.46) | -3.09 (-4.18,-2.00) | -2.84 (-3.79,-1.89) | -2.66 (-3.70,-1.61) | -0.50 (-1.18,0.18) | -0.32 (-1.08,0.44) | -0.14 (-1.00,0.72) | -0.02 (-0.88,0.84) | Liangge San | 0.06 (-0.78,0.90) | 0.53 (-0.05,1.11) |
| -3.76 (-5.02,-2.51) | -3.16 (-4.26,-2.05) | -2.90 (-3.86,-1.93) | -2.72 (-3.78,-1.66) | -0.56 (-1.26,0.14) | -0.38 (-1.16,0.40) | -0.20 (-1.08,0.68) | -0.08 (-0.96,0.80) | -0.06 (-0.90,0.78) | Dahuang Fuzi Tang | 0.47 (-0.13,1.08) |
| -4.24 (-5.34,-3.14) | -3.63 (-4.55,-2.70) | -3.37 (-4.12,-2.62) | -3.19 (-4.06,-2.32) | -1.03 (-1.39,-0.67) | -0.85 (-1.35,-0.36) | -0.67 (-1.31,-0.03) | -0.55 (-1.19,0.09) | -0.53 (-1.11,0.05) | -0.47 (-1.08,0.13) | Conventional Therapy |

**Note:** League table showing pairwise comparisons of treatments for Gastrointestinal Dysfunction (GID) score. Values represent mean differences (MD) with 95% confidence intervals (CI). Positive values favor the treatment in the row, negative values favor the treatment in the column.

**Supplementary Table 6I. League table of IAP**

| Tiaoqi Tongfu Tang | Zengye Chengqi Tang | Banxia Xiexin Tang | Intestinal Comfort | Sijunzi Tang | Rhubarb | Xuebijing | Houpo Heji | Liangge San | Dahuang Fuzi Tang | Dachengqi Tang | Conventional Therapy | Yantiao Fang |
| --- | --- | --- | --- | --- | --- | --- | --- | --- | --- | --- | --- | --- |
| Tiaoqi Tongfu Tang | 1.65 (-6.31,9.61) | 1.99 (-3.71,7.69) | 2.03 (-6.01,10.07) | 2.09 (-5.98,10.16) | 2.65 (-2.15,7.46) | 2.63 (-5.47,10.73) | 2.94 (-5.00,10.89) | 2.88 (-5.23,10.99) | 3.14 (-4.84,11.12) | 3.64 (-2.78,10.05) | 4.93 (0.77,9.09) | 5.88 (-0.50,12.26) |
| -1.65 (-9.61,6.31) | Zengye Chengqi Tang | 0.33 (-7.49,8.16) | 0.38 (-9.28,10.04) | 0.44 (-9.25,10.13) | 1.00 (-6.20,8.20) | 0.98 (-8.73,10.69) | 1.29 (-8.29,10.87) | 1.23 (-8.49,10.95) | 1.49 (-8.12,11.10) | 1.98 (-6.37,10.34) | 3.28 (-3.50,10.06) | 4.23 (-4.10,12.56) |
| -1.99 (-7.69,3.71) | -0.33 (-8.16,7.49) | Banxia Xiexin Tang | 0.05 (-7.86,7.95) | 0.11 (-7.83,8.04) | 0.67 (-3.91,5.25) | 0.65 (-7.33,8.62) | 0.96 (-6.85,8.77) | 0.90 (-7.08,8.88) | 1.16 (-6.69,9.00) | 1.65 (-4.60,7.90) | 2.95 (-0.95,6.84) | 3.89 (-2.32,10.10) |
| -2.03 (-10.07,6.01) | -0.38 (-10.04,9.28) | -0.05 (-7.95,7.86) | Intestinal Comfort | 0.06 (-9.69,9.81) | 0.62 (-6.67,7.91) | 0.60 (-9.18,10.38) | 0.91 (-8.74,10.56) | 0.85 (-8.94,10.64) | 1.11 (-8.57,10.79) | 1.60 (-6.83,10.04) | 2.90 (-3.98,9.78) | 3.85 (-4.56,12.25) |
| -2.09 (-10.16,5.98) | -0.44 (-10.13,9.25) | -0.11 (-8.04,7.83) | -0.06 (-9.81,9.69) | Sijunzi Tang | 0.56 (-6.76,7.88) | 0.54 (-9.27,10.35) | 0.85 (-8.83,10.53) | 0.79 (-9.03,10.61) | 1.05 (-8.66,10.76) | 1.54 (-6.92,10.01) | 2.84 (-4.08,9.76) | 3.79 (-4.65,12.22) |
| -2.65 (-7.46,2.15) | -1.00 (-8.20,6.20) | -0.67 (-5.25,3.91) | -0.62 (-7.91,6.67) | -0.56 (-7.88,6.76) | Rhubarb | -0.02 (-7.38,7.34) | 0.29 (-6.89,7.47) | 0.23 (-7.14,7.60) | 0.49 (-6.73,7.71) | 0.98 (-4.46,6.43) | 2.28 (-0.13,4.69) | 3.23 (-2.18,8.63) |
| -2.63 (-10.73,5.47) | -0.98 (-10.69,8.73) | -0.65 (-8.62,7.33) | -0.60 (-10.38,9.18) | -0.54 (-10.35,9.27) | 0.02 (-7.34,7.38) | Xuebijing | 0.31 (-9.39,10.01) | 0.25 (-9.59,10.09) | 0.51 (-9.22,10.24) | 1.00 (-7.49,9.50) | 2.30 (-4.65,9.25) | 3.25 (-5.22,11.72) |
| -2.94 (-10.89,5.00) | -1.29 (-10.87,8.29) | -0.96 (-8.77,6.85) | -0.91 (-10.56,8.74) | -0.85 (-10.53,8.83) | -0.29 (-7.47,6.89) | -0.31 (-10.01,9.39) | Houpo Heji | -0.06 (-9.77,9.65) | 0.20 (-9.40,9.80) | 0.69 (-7.65,9.04) | 1.99 (-4.78,8.76) | 2.94 (-5.38,11.25) |
| -2.88 (-10.99,5.23) | -1.23 (-10.95,8.49) | -0.90 (-8.88,7.08) | -0.85 (-10.64,8.94) | -0.79 (-10.61,9.03) | -0.23 (-7.60,7.14) | -0.25 (-10.09,9.59) | 0.06 (-9.65,9.77) | Liangge San | 0.26 (-9.48,10.00) | 0.75 (-7.75,9.26) | 2.05 (-4.91,9.01) | 3.00 (-5.48,11.47) |
| -3.14 (-11.12,4.84) | -1.49 (-11.10,8.12) | -1.16 (-9.00,6.69) | -1.11 (-10.79,8.57) | -1.05 (-10.76,8.66) | -0.49 (-7.71,6.73) | -0.51 (-10.24,9.22) | -0.20 (-9.80,9.40) | -0.26 (-10.00,9.48) | Dahuang Fuzi Tang | 0.49 (-7.88,8.87) | 1.79 (-5.02,8.60) | 2.74 (-5.61,11.09) |
| -3.64 (-10.05,2.78) | -1.98 (-10.34,6.37) | -1.65 (-7.90,4.60) | -1.60 (-10.04,6.83) | -1.54 (-10.01,6.92) | -0.98 (-6.43,4.46) | -1.00 (-9.50,7.49) | -0.69 (-9.04,7.65) | -0.75 (-9.26,7.75) | -0.49 (-8.87,7.88) | Dachengqi Tang | 1.30 (-3.59,6.18) | 2.24 (-4.63,9.11) |
| -4.93 (-9.09,-0.77) | -3.28 (-10.06,3.50) | -2.95 (-6.84,0.95) | -2.90 (-9.78,3.98) | -2.84 (-9.76,4.08) | -2.28 (-4.69,0.13) | -2.30 (-9.25,4.65) | -1.99 (-8.76,4.78) | -2.05 (-9.01,4.91) | -1.79 (-8.60,5.02) | -1.30 (-6.18,3.59) | Conventional Therapy | 0.95 (-3.89,5.78) |
| -5.88 (-12.26,0.50) | -4.23 (-12.56,4.10) | -3.89 (-10.10,2.32) | -3.85 (-12.25,4.56) | -3.79 (-12.22,4.65) | -3.23 (-8.63,2.18) | -3.25 (-11.72,5.22) | -2.94 (-11.25,5.38) | -3.00 (-11.47,5.48) | -2.74 (-11.09,5.61) | -2.24 (-9.11,4.63) | -0.95 (-5.78,3.89) | Yantiao Fang |

**Note:** League table showing pairwise comparisons of treatments for Gastrointestinal Dysfunction (GID) score. Values represent mean differences (MD) with 95% confidence intervals (CI). Positive values favor the treatment in the row, negative values favor the treatment in the column.

**Supplementary Table 7A. Cumulative probability table of Gastrointestinal dysfunction score**

| Treatmt | SUCRA | PrBest | MeanRank |
| --- | --- | --- | --- |
| Banxia Xiexin Tang | 64.5 | 0.4 | 6.3 |
| Xuebijing | 32.0 | 0.6 | 11.2 |
| Conventional Therapy | 7.7 | 0.0 | 14.8 |
| Dachaihu Tang | 92.7 | 78.3 | 2.1 |
| Dachengqi Tang | 73.3 | 5.0 | 5.0 |
| Dahuang Fuzi Tang | 41.3 | 0.0 | 9.8 |
| Dahuang Gancao Tang | 80.6 | 6.2 | 3.9 |
| Dahuang Mudan Tang | 40.7 | 1.2 | 9.9 |
| Houpo Heji | 25.7 | 0.0 | 12.1 |
| Intestinal Comfort | 46.6 | 0.5 | 9.0 |
| Rhubarb | 70.7 | 0.6 | 5.4 |
| Sijunzi Tang | 30.6 | 0.1 | 11.4 |
| Tiaoqi Tongfu Tang | 38.4 | 0.3 | 10.2 |
| Yantiao Fang | 53.6 | 3.9 | 8.0 |
| Yiqi Tang | 48.9 | 0.3 | 8.7 |
| Zengye Chengqi Tang | 52.6 | 2.5 | 8.1 |

**Note:** Cumulative probability table for Gastrointestinal dysfunction score showing treatment rankings; **SUCRA**: surface under the cumulative ranking curve, **PrBest**: probability of being the best, **MeanRank**: average rank. Higher values indicate better performance.

**Supplementary Table 7B. Cumulative probability table of TCM symptom score**

| Treatmt | SUCRA | PrBest | MeanRank |
| --- | --- | --- | --- |
| Banxia Xiexin Tang | 46.70 | 0.60 | 6.30 |
| Conventional Therapy | 3.50 | 0.00 | 10.70 |
| Dachaihu Tang | 57.80 | 13.60 | 5.20 |
| Dahuang Fuzi Tang | 78.30 | 16.40 | 3.20 |
| Liangge San | 49.80 | 8.60 | 6.00 |
| Sijunzi Tang | 55.60 | 15.80 | 5.40 |
| Tiaoqi Tongfu Tang | 51.00 | 1.80 | 5.90 |
| Xuebijing | 24.80 | 0.90 | 8.50 |
| Yantiao Fang | 47.10 | 1.50 | 6.30 |
| Yiqi Tang | 78.80 | 36.30 | 3.10 |
| Zengye Chengqi Tang | 56.50 | 4.60 | 5.30 |

**Note:** Cumulative probability table for TCM symptom score showing treatment rankings; **SUCRA**: surface under the cumulative ranking curve, **PrBest**: probability of being the best, **MeanRank**: average rank. Higher values indicate better performance.

**Supplementary Table 7C. Cumulative probability table of Apache Ⅱ score**

| Treatmt | SUCRA | PrBest | MeanRank |
| --- | --- | --- | --- |
| Banxia Xiexin Tang | 33.90 | 0.00 | 10.90 |
| Xuebijing | 24.20 | 0.30 | 12.40 |
| Conventional Therapy | 7.50 | 0.00 | 14.90 |
| Dachaihu Tang | 71.70 | 28.30 | 5.20 |
| Dachengqi Tang | 52.50 | 3.50 | 8.10 |
| Dahuang Fuzi Tang | 52.90 | 0.10 | 8.10 |
| Dahuang Mudan Tang | 51.20 | 2.90 | 8.30 |
| Houpo Heji | 58.30 | 5.50 | 7.30 |
| Intestinal Comfort | 30.50 | 0.50 | 11.40 |
| Liangge San | 48.90 | 3.10 | 8.70 |
| Rhubarb | 75.10 | 2.80 | 4.70 |
| Sijunzi Tang | 42.60 | 1.60 | 9.60 |
| Tiaoqi Tongfu Tang | 89.70 | 33.20 | 2.50 |
| Yantiao Fang | 27.10 | 0.40 | 11.90 |
| Yiqi Tang | 76.70 | 13.00 | 4.50 |
| Zengye Chengqi Tang | 57.30 | 4.90 | 7.40 |

**Note:** Cumulative probability table for Apache Ⅱ score showing treatment rankings; **SUCRA**: surface under the cumulative ranking curve, **PrBest**: probability of being the best, **MeanRank**: average rank. Higher values indicate better performance.

**Supplementary Table 7D. Cumulative probability table of AGI classification**

| Treatmt | SUCRA | PrBest | MeanRank |
| --- | --- | --- | --- |
| Conventional Therapy | 3.4 | 0.0 | 3.9 |
| Dahuang Fuzi Tang | 92.5 | 78.7 | 1.2 |
| Rhubarb | 43.7 | 3.8 | 2.7 |
| Zengye Chengqi Tang | 60.4 | 17.5 | 2.2 |

**Note:** Cumulative probability table for AGI classification showing treatment rankings; **SUCRA**: surface under the cumulative ranking curve, **PrBest**: probability of being the best, **MeanRank**: average rank. Higher values indicate better performance.

**Supplementary Table 7E. Cumulative probability table of D-lactic acid**

| Treatmt | SUCRA | PrBest | MeanRank |
| --- | --- | --- | --- |
| Banxia Xiexin Tang | 79.6 | 19.5 | 3.4 |
| Conventional Therapy | 15.3 | 0 | 11.2 |
| Dahuang Fuzi Tang | 31.5 | 1.4 | 9.2 |
| Dahuang Mudan Tang | 45.6 | 1 | 7.5 |
| Houpo Heji | 36.4 | 1.9 | 8.6 |
| Intestinal Comfort | 67.5 | 7.9 | 4.9 |
| Liangge San | 33.5 | 1.7 | 9 |
| Rhubarb | 50.5 | 5.7 | 6.9 |
| Sijunzi Tang | 81.8 | 35.9 | 3.2 |
| Tiaoqi Tongfu Tang | 68 | 15.8 | 4.8 |
| Yantiao Fang | 36.3 | 0.4 | 8.6 |
| Yiqi Tang | 54.8 | 7.3 | 6.4 |
| Zengye Chengqi Tang | 49.1 | 1.5 | 7.1 |

**Note:** Cumulative probability table for D-lactic acid showing treatment rankings; **SUCRA**: surface under the cumulative ranking curve, **PrBest**: probability of being the best, **MeanRank**: average rank. Higher values indicate better performance.

**Supplementary Table 7F. Cumulative probability table of DAO mean**

| Treatmt | SUCRA | PrBest | MeanRank |
| --- | --- | --- | --- |
| Banxia Xiexin Tang | 36.2 | 0.4 | 7.4 |
| Xuebijing | 90.2 | 58.3 | 2.0 |
| Conventional Therapy | 12.3 | 0.0 | 9.8 |
| Houpo Heji | 69.4 | 14.9 | 4.1 |
| Intestinal Comfort | 58.9 | 3.3 | 5.1 |
| Liangge San | 46.3 | 3.3 | 6.4 |
| Rhubarb | 42.5 | 2.8 | 6.7 |
| Sijunzi Tang | 52.6 | 5.5 | 5.7 |
| Tiaoqi Tongfu Tang | 59.8 | 8.8 | 5.0 |
| Yantiao Fang | 32.4 | 1.2 | 7.8 |
| Zengye Chengqi Tang | 49.5 | 1.4 | 6.0 |

**Note:** Cumulative probability table for DAO mean showing treatment rankings; **SUCRA**: surface under the cumulative ranking curve, **PrBest**: probability of being the best, **MeanRank**: average rank. Higher values indicate better performance.

**Supplementary Table 7G. Cumulative probability table of TNF-a**

| Treatmt | SUCRA | PrBest | MeanRank |
| --- | --- | --- | --- |
| Banxia Xiexin Tang | 33.2 | 0.0 | 9.0 |
| Conventional Therapy | 15.5 | 0.0 | 11.1 |
| Dahuang Fuzi Tang | 34.2 | 0.0 | 8.9 |
| Dahuang Mudan Tang | 48.5 | 0.0 | 7.2 |
| Houpo Heji | 83.9 | 1.1 | 2.9 |
| Intestinal Comfort | 67.7 | 0.0 | 4.9 |
| Liangge San | 31.0 | 0.0 | 9.3 |
| Rhubarb | 38.4 | 0.0 | 8.4 |
| Sijunzi Tang | 68.5 | 0.0 | 4.8 |
| Tiaoqi Tongfu Tang | 99.9 | 98.8 | 1.0 |
| Yantiao Fang | 32.2 | 0.0 | 9.1 |
| Yiqi Tang | 67.0 | 0.1 | 5.0 |
| Zengye Chengqi Tang | 30.0 | 0.0 | 9.4 |

**Note:** Cumulative probability table for TNF-a showing treatment rankings; **SUCRA**: surface under the cumulative ranking curve, **PrBest**: probability of being the best, **MeanRank**: average rank. Higher values indicate better performance.

**Supplementary Table 7H. Cumulative probability table of IL-6**

| Treatment | SUCRA | PrBest | MeanRank |
| --- | --- | --- | --- |
| Banxia Xiexin Tang | 27.8 | 0.0 | 8.2 |
| Conventional Therapy | 1.7 | 0.0 | 10.8 |
| Dahuang Fuzi Tang | 23.4 | 0.0 | 8.7 |
| Houpo Heji | 96.2 | 73.8 | 1.4 |
| Intestinal Comfort | 43.8 | 0.0 | 6.6 |
| Liangge San | 26.5 | 0.0 | 8.3 |
| Rhubarb | 53.0 | 0.0 | 5.7 |
| Sijunzi Tang | 86.1 | 17.4 | 2.4 |
| Tiaoqi Tongfu Tang | 80.6 | 5.4 | 2.9 |
| Yantiao Fang | 33.9 | 0.0 | 7.6 |
| Yiqi Tang | 77.1 | 3.5 | 3.3 |

**Note:** Cumulative probability table for IL-6 showing treatment rankings; **SUCRA**: surface under the cumulative ranking curve, **PrBest**: probability of being the best, **MeanRank**: average rank. Higher values indicate better performance.

**Supplementary Table 7I. Cumulative probability table of IAP**

| Treatmt | SUCRA | PrBest | MeanRank |
| --- | --- | --- | --- |
| Banxia Xiexin Tang | 60.1 | 4.3 | 5.8 |
| Conventional Therapy | 22.9 | 0.0 | 10.3 |
| Dachengqi Tang | 41.7 | 1.9 | 8.0 |
| Dahuang Fuzi Tang | 47.3 | 7.4 | 7.3 |
| Houpo Heji | 49.2 | 7.8 | 7.1 |
| Intestinal Comfort | 57.4 | 12.4 | 6.1 |
| Liangge San | 49.8 | 8.6 | 7.0 |
| Rhubarb | 53.1 | 0.3 | 6.6 |
| Sijunzi Tang | 56.6 | 12.2 | 6.2 |
| Tiaoqi Tongfu Tang | 79.2 | 20.6 | 3.5 |
| Xuebijing | 52.1 | 10.2 | 6.7 |
| Yantiao Fang | 19.8 | 0.2 | 10.6 |
| Zengye Chengqi Tang | 60.9 | 14.2 | 5.7 |

**Note:** Cumulative probability table for IAP showing treatment rankings; **SUCRA**: surface under the cumulative ranking curve, **PrBest**: probability of being the best, **MeanRank**: average rank. Higher values indicate better performance.

**Supplementary Table 8. Adverse reaction**

| Author | Intervention | | Adverse reaction | | | | |
| --- | --- | --- | --- | --- | --- | --- | --- |
|  | Intervention | Control | Nausea and Vomiting | Regurgitation | Abdominal distension | Diarrhea | Gastric residue>500ml |
| Hu Xiong | Dahuang Fuzi Tang | CON | T：6 C：7 | T：2 C：8 | T：5 C：13 | T：3 C：2 | T：5 C：7 |
| Liu Yongcheng | Dahuang Fuzi Tang | CON | T：1 C：3 | T：1 C：4 | T：1 C：2 | T：2 C：5 | / |
| Liang Futing | Dahuang Fuzi Tang | CON | T：6 C：8 | T：2 C：9 | T：6 C：14 | T：3 C：2 | T：5 C：8 |
| Huang Dan | Dahuang Fuzi Tang | CON | T：5 C：7 | T：3 C：8 | T：5 C：12 | T：3 C：2 | T：4 C：7 |

**Note:** Summary of reported adverse reactions in the included studies. Values represent the number of patients experiencing each type of adverse event in each treatment group. **Con**:conventional treatment, **T**: experimental group, **C**: control group.

**Supplementary Table 9. τ² estimator and network-level τ²/I²**

|  | Gastrointestinal dysfunction score | TCM symptom score | Apache Ⅱ score | AGI classification | D-lactic acid | DAO mean | TNF-a | IL-6 | IAP |
| --- | --- | --- | --- | --- | --- | --- | --- | --- | --- |
| τ² estimator | 0.199 | 3.293​ | 8.615​ | 1.168E-25 | 1.6246​ | 1.6170​ | 44.1546​ | 17.0047​ | 11.8016​ |
| network-level τ²/I² | 0.335 | 5.010 | 9.310 | 0.330 | 201.810 | 2.310 | 69.160 | 102.300 | 12.340 |

**Note:** τ² indicates the between-study variance in the network meta-analysis. I² represents the proportion of total variation due to heterogeneity rather than chance. Values are reported for each outcome at the network level.

**Supplementary Table 10. design-by-treatment interaction**

|  | Gastrointestinal dysfunction score | TCM symptom score | Apache Ⅱ score | AGI classification | D-lactic acid | DAO mean | TNF-a | IL-6 | IAP |
| --- | --- | --- | --- | --- | --- | --- | --- | --- | --- |
| χ²(1) | 1.96 | 9.55 | 3.37 | 3.22 | 1.76 | 2.13 | 0.11 | 52.03 | 0.90 |
| Prob > χ² | 0.16 | 0.00 | 0.07 | 0.07 | 0.18 | 0.14 | 0.74 | 0.00 | 0.34 |

**Note:** Design by treatment interaction test evaluates the impact of different research designs on the effect size; Prob>χ ²<0.05 indicates a significant interaction between design type and intervention effect.

**Supplementary Table 11. Begg's Test**

|  | Gastrointestinal dysfunction score | TCM symptom score | Apache Ⅱ score | AGI classification | D-lactic acid | DAO mean | TNF-a | IL-6 | IAP |
| --- | --- | --- | --- | --- | --- | --- | --- | --- | --- |
| P>\|t\| | 0.415 | 0.719 | 0.012 | 0.443 | 0.951 | 0.765 | 0.000 | 0.001 | 0.436 |

**Note:** Begg's rank correlation test is used to evaluate whether there is publication bias in each outcome measure; A P>| t | value less than 0.05 is considered a significant risk of bias. Only the P-values of "Apache II score", "TNF - α", and "IL-6" in the table are less than 0.05, indicating possible publication bias. There is no significant evidence of asymmetry in other indicators.
