# Supplementary material for: The impact of traditional Chinese medicine on gastrointestinal dysfunction in sepsis patients: a systematic review and network meta-analysis
Source: Front Pharmacol. 2025 Oct 10;16:1648809. doi: 10.3389/fphar.2025.1648809 (PMC12549693; doi:10.3389/fphar.2025.1648809)
Supplement: Supplementary file 2 [file Supplementaryfile2.docx]

**Supplementary Material 1: Extended Mechanistic Discussion**

In Dachaihu Tang, anthraquinones from Rheum palmatum—such as rhein—suppress the expression of vascular adhesion molecules (VCAM-1 and ICAM-1), thereby improving intestinal microcirculatory perfusion (Jin et al., 2020).

In Yiqi Tang, codonopsis polysaccharides promote intestinal epithelial proliferation and mucus secretion via the TLR4/MyD88 signaling pathway, while atractylenolide III restores epithelial tight junction integrity and reduces mucosal permeability by inhibiting the NF-κB pathway. These metabolites work synergistically to enhance gastrointestinal motility and reduce bacterial translocation, contributing to the protection of intestinal barrier function in sepsis (Miao et al., 2022).

Tiaoqi Tongfu Tang exerts its effects through magnolol, which alleviates abdominal pain by inhibiting L-type calcium channels in gastrointestinal smooth muscle (Xu et al., 2012; Zhang et al., 2013). Collectively, these three formulations exert integrated regulatory effects across the “motility–inflammation–barrier” axis central to the pathophysiology of sepsis-induced gastrointestinal dysfunction.

In Tiaoqi Tongfu Tang, magnolol inhibits IKK kinase activity, thereby blocking TNF-α-induced IκBα phosphorylation and degradation, and suppressing phosphorylation and nuclear translocation of the NF-κB p65 subunit. This cascade ultimately downregulates the expression of NF-κB-dependent pro-inflammatory genes (Tse et al., 2005). In addition, forsythoside A inhibits the phosphorylation of p38, JNK, ERK, and p65, thereby blocking the p38/JNK/MAPK/ERK and IL-1β signaling pathways, resulting in decreased release of inflammatory cytokines such as TNF-α, IL-6, and IL-1β (Song and Lei, 2025).

In Yiqi Tang, rhubarb exhibits anti-inflammatory activity by modulating the NF-κB and MAPK signaling pathways, effectively reducing the secretion of IL-6 and other pro-inflammatory mediators. Moreover, polysaccharides derived from Codonopsis pilosula modulate the TLR4 pathway, inhibit excessive activation of regulatory T (Treg) cells, restore CD4⁺ T cell function, and promote Th1 immune responses. This corrects Th2 polarization, thereby improving systemic inflammatory responses and restoring immune homeostasis (Zheng et al., 2014). Both formulations exert synergistic regulatory effects across the inflammation–immunity–injury axis, thereby contributing to the alleviation of sepsis-associated gastrointestinal dysfunction.

In Dahuang Fuzi Tang, emodin also enhances M3 cholinergic neurotransmission and smooth muscle contractility, improving gastrointestinal motility in the context of sepsis-induced dysfunction (Fu et al., 2025). Additionally, alkaloids derived from Aconitum species downregulate the PI3K/AKT/NF-κB signaling pathway, effectively inhibiting the production of TNF-α and IL-6, and mitigating local inflammation. These metabolites also upregulate tight junction proteins such as ZO-1, Occludin, and Claudin-1, thereby strengthening the intestinal epithelial barrier and preventing translocation of harmful substances (Zhang et al., 2022).

Xuebijing Injection modulates the PI3K-AKT, NF-κB, MAPK, and Toll-like receptor signaling pathways, resulting in a significant reduction in pro-inflammatory cytokines including IL-2, IL-4, and TNF-α. It also alleviates oxidative stress and mucosal injury (Chen et al., 2024). Both agents promote mucosal repair in sepsis-associated gastrointestinal dysfunction through anti-inflammatory, antioxidant, and barrier-enhancing mechanisms.

**References**

Chen, M., Shu, W., Zhang, J., Huang, H., and Liu, J. (2024). Mechanisms and clinical application of Xuebijing injection, a traditional Chinese herbal medicine–a systematic review. *Advances in Traditional Medicine* 24(2)**,** 403-412. doi: 10.1007/s13596-023-00702-5.

Fu, W., Liu, S.-c., Xu, T.-x., Liu, Y., Zhang, T., Liu, D.-j., et al. (2025). Emodin Inhibits NLRP3 Inflammasome Activation and Protects Against Sepsis via Promoting FUNDC1-Mediated Mitophagy. *International Journal of Biological Sciences* 21(8)**,** 3631-3648. doi: 10.7150/ijbs.110904.

Jin, L., Schmiech, M., El Gaafary, M., Zhang, X., Syrovets, T., and Simmet, T. (2020). A comparative study on root and bark extracts of Eleutherococcus senticosus and their effects on human macrophages. *Phytomedicine* 68**,** 153181. doi: 10.1016/j.phymed.2020.153181.

Miao, Y., Zhang, C., Yang, L., Zeng, X., Hu, Y., Xue, X., et al. (2022). The activation of PPARγ enhances Treg responses through up-regulating CD36/CPT1-mediated fatty acid oxidation and subsequent N-glycan branching of TβRII/IL-2Rα. *Cell Commun Signal* 20(1)**,** 48. doi: 10.1186/s12964-022-00849-9.

Song, L., and Lei, Y. (2025). Forsythoside A inhibited inflammatory response by inhibiting p38 JNK/MAPK/ERK and NF-κB signaling in Staphylococcus aureus pneumonia. *J Mol Histol* 56(3)**,** 147. doi: 10.1007/s10735-025-10418-2.

Tse, A.K., Wan, C.K., Shen, X.L., Yang, M., and Fong, W.F. (2005). Honokiol inhibits TNF-alpha-stimulated NF-kappaB activation and NF-kappaB-regulated gene expression through suppression of IKK activation. *Biochem Pharmacol* 70(10)**,** 1443-1457. doi: 10.1016/j.bcp.2005.08.011.

Xu, J.D., Liu, S., Wang, W., Li, L.S., Li, X.F., Li, Y., et al. (2012). Emodin induces chloride secretion in rat distal colon through activation of mast cells and enteric neurons. *Br J Pharmacol* 165(1)**,** 197-207. doi: 10.1111/j.1476-5381.2011.01573.x.

Zhang, M., Lian, B., Zhang, R., Guo, Y., Zhao, J., He, S., et al. (2022). Emodin Ameliorates Intestinal Dysfunction by Maintaining Intestinal Barrier Integrity and Modulating the Microbiota in Septic Mice. *Mediators Inflamm* 2022**,** 5026103. doi: 10.1155/2022/5026103.

Zhang, M., Zang, K.H., Luo, J.L., Leung, F.P., Huang, Y., Lin, C.Y., et al. (2013). Magnolol inhibits colonic motility through down-regulation of voltage-sensitive L-type Ca2+ channels of colonic smooth muscle cells in rats. *Phytomedicine* 20(14)**,** 1272-1279. doi: 10.1016/j.phymed.2013.07.008.

Zheng, Y.S., Wu, Z.S., Ni, H.B., Ke, L., Tong, Z.H., Li, W.Q., et al. (2014). Codonopsis pilosula polysaccharide attenuates cecal ligation and puncture sepsis via circuiting regulatory T cells in mice. *Shock* 41(3)**,** 250-255. doi: 10.1097/shk.0000000000000091.

**Supplementary Material 2: Sensitivity analysis**

In this study, we conducted sensitivity analysis on all primary and secondary outcome measures, using influence analysis (meta-analysis) to evaluate the impact of individual studies on the combined effect size one by one. The results showed that all outcome indicators remained stable during the analysis process, indicating that the overall conclusion has good robustness.


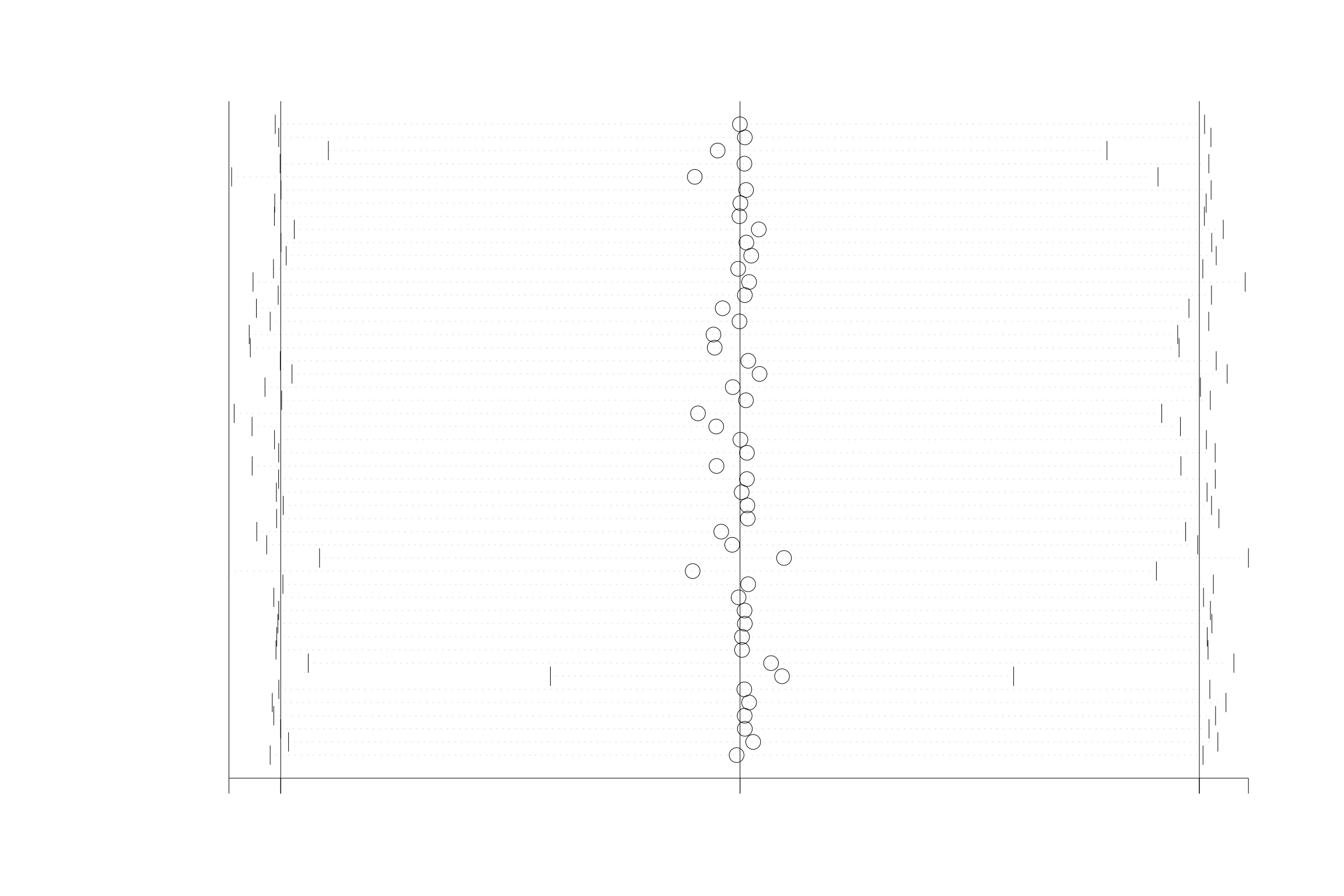


Figure 1. Sensitivity Analysis of Gastrointestinal dysfunction score


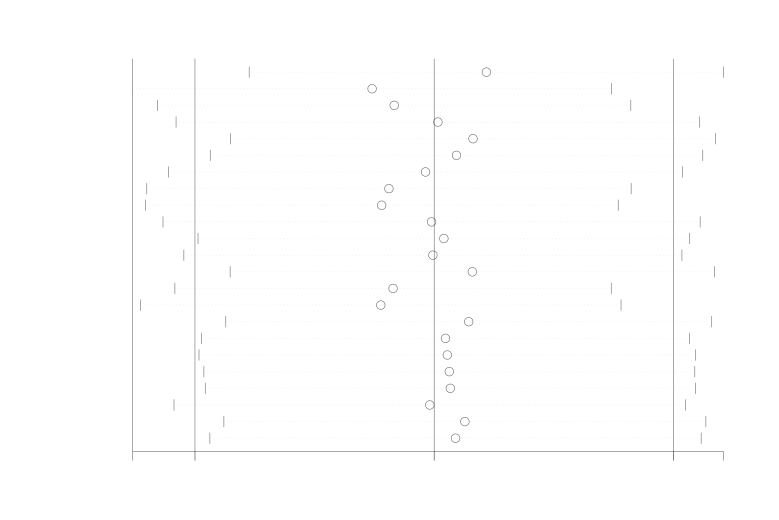


Figure 2. Sensitivity Analysis of TCM symptom score


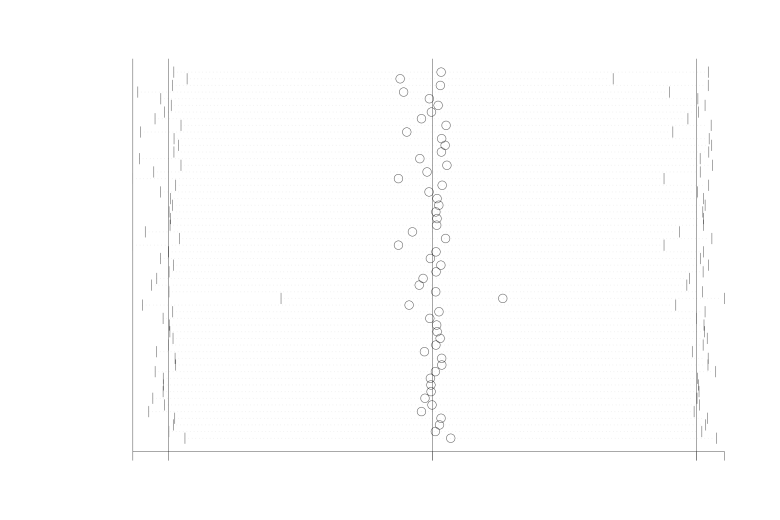


Figure 3. Sensitivity Analysis of Apache Ⅱ score


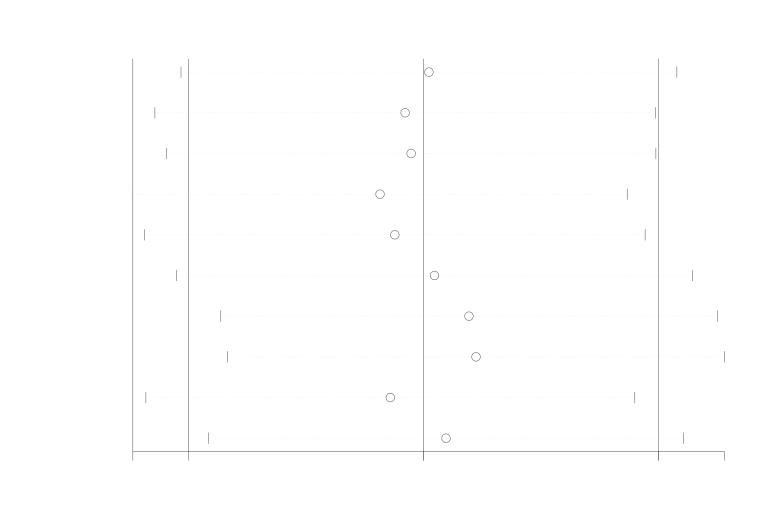


Figure 4. Sensitivity Analysis of AGI classification


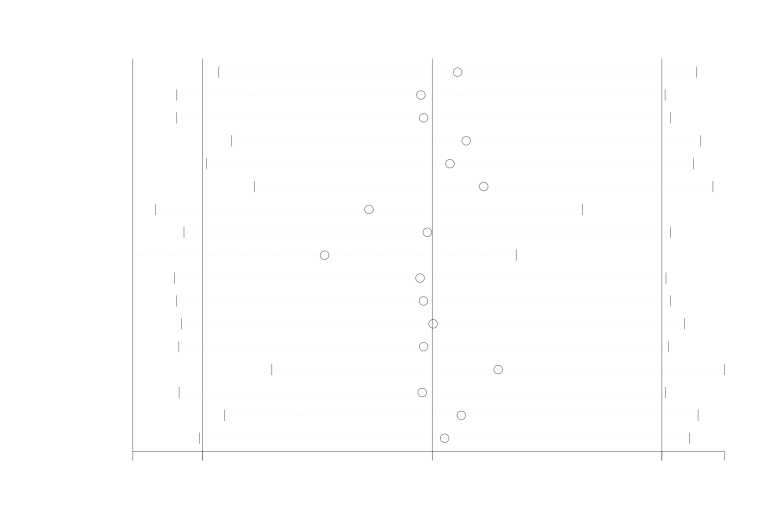


Figure 5. Sensitivity Analysis of D-lactic acid


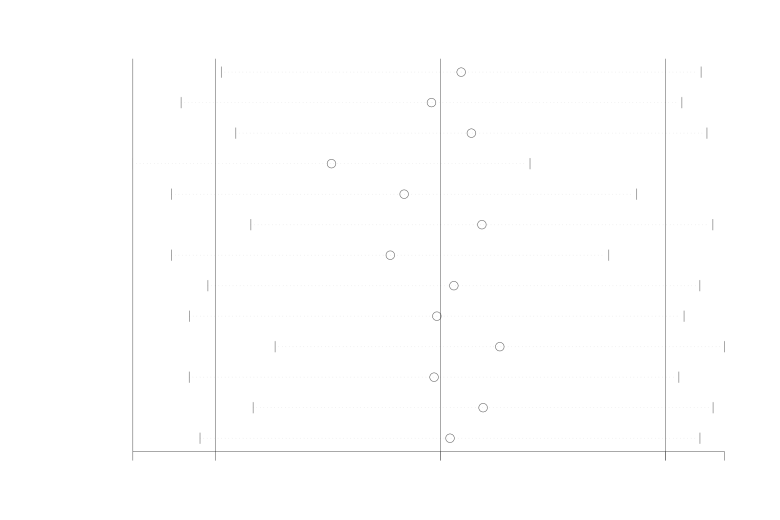


Figure 6. Sensitivity Analysis of DAO mean


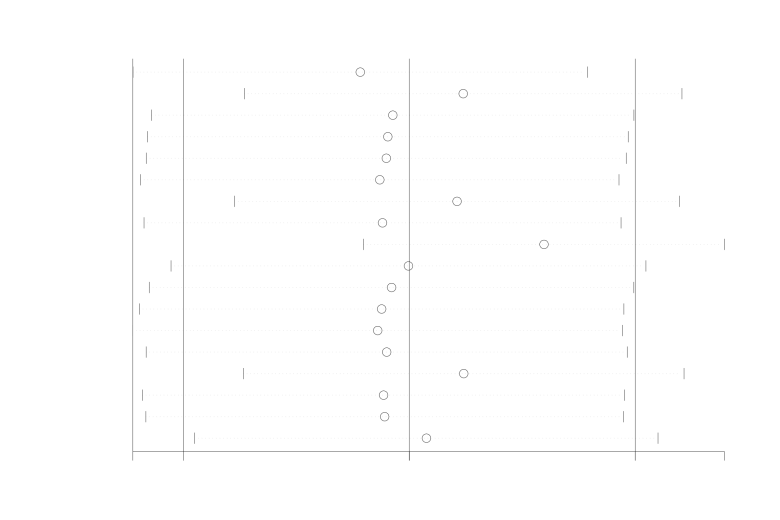


Figure 7. Sensitivity Analysis of TNF-a


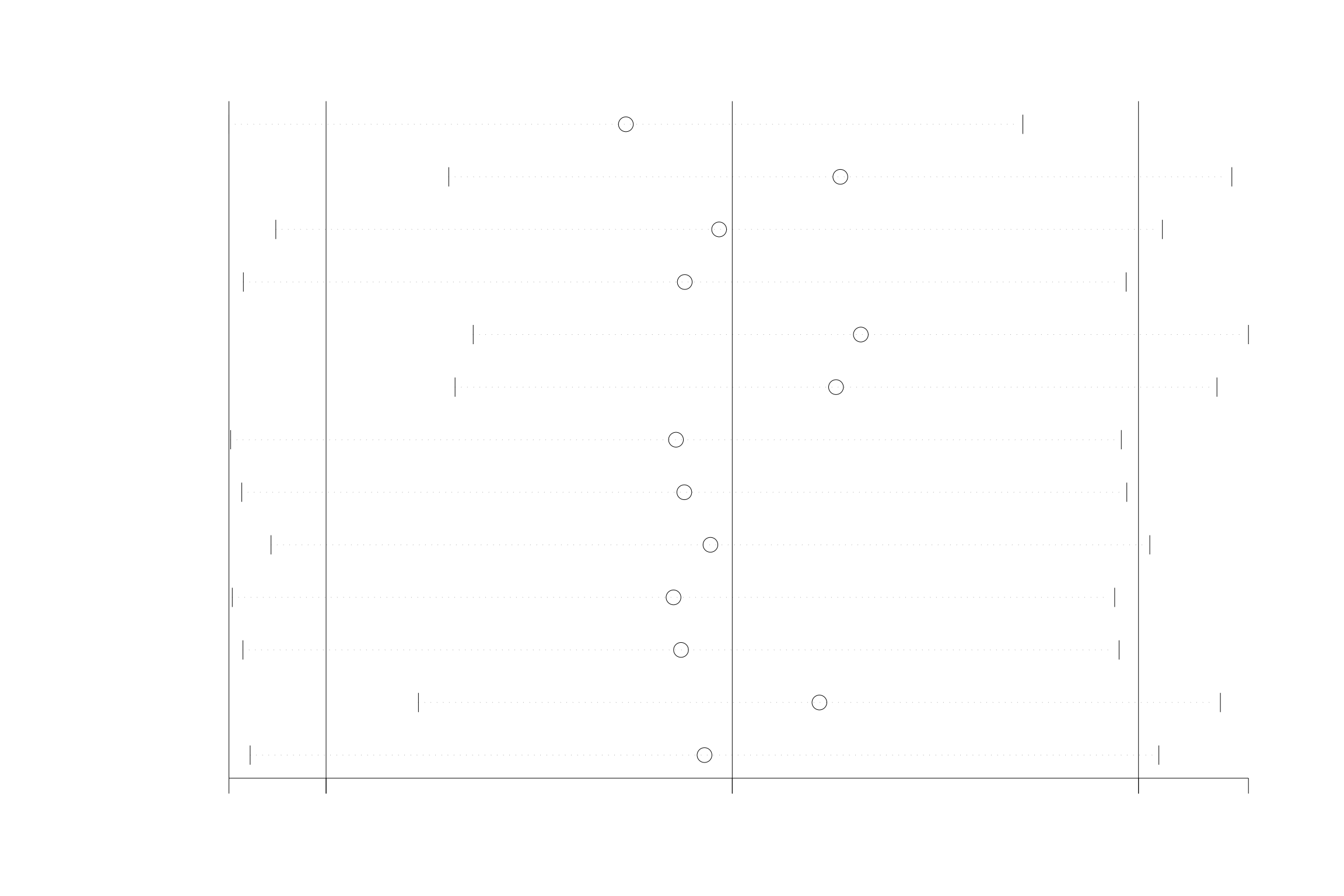

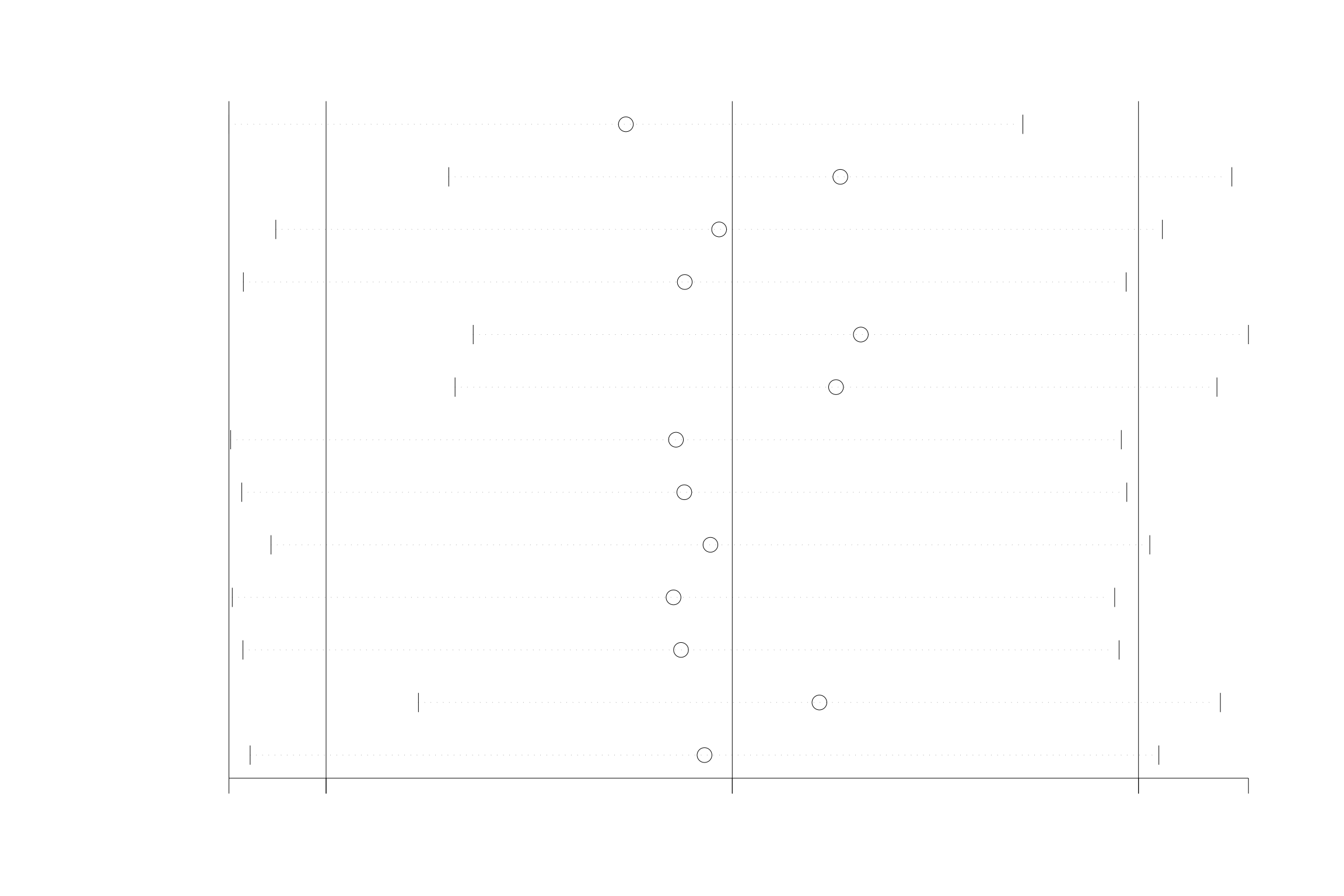


Figure 8. Sensitivity Analysis of IL-6


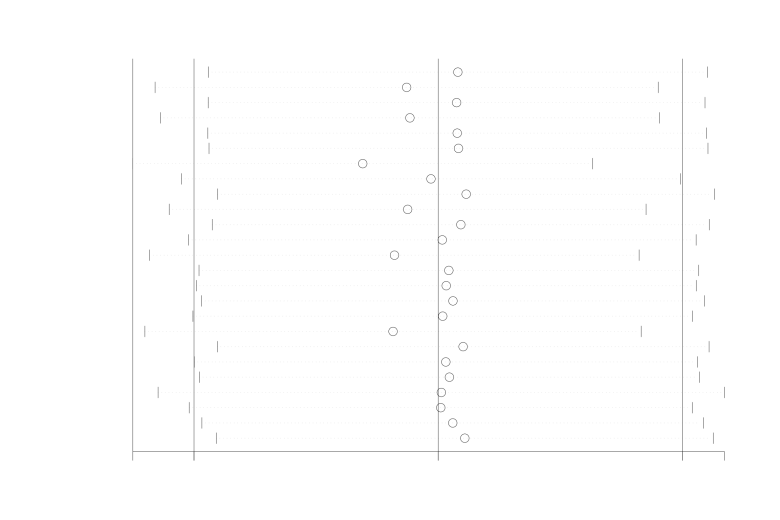


Figure 9. Sensitivity Analysis of IAP

**Supplementary Material 3：Trim-and-fill analyze**

**
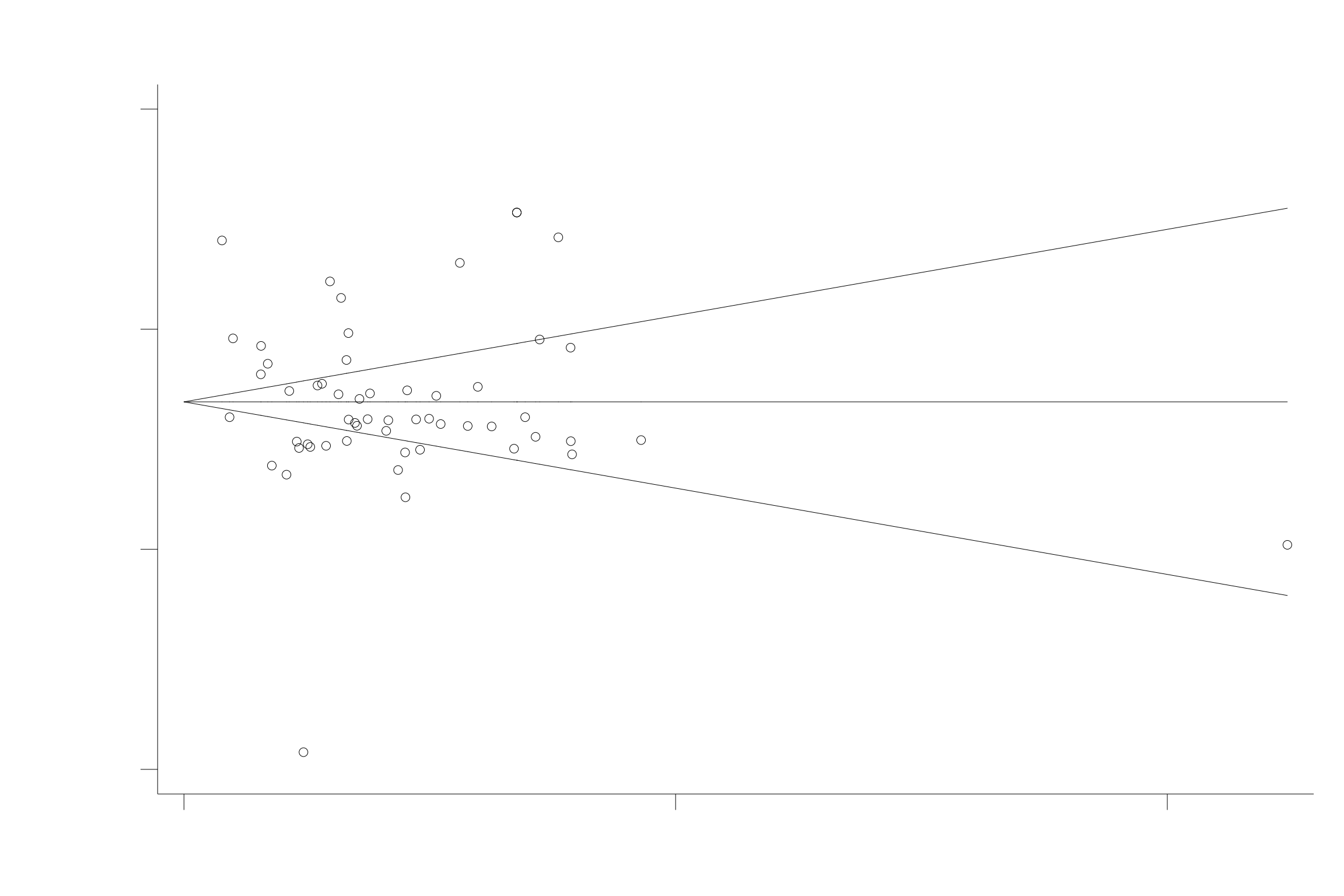
**

Figure 1. Trim-and-fill analyze of Apache Ⅱ score


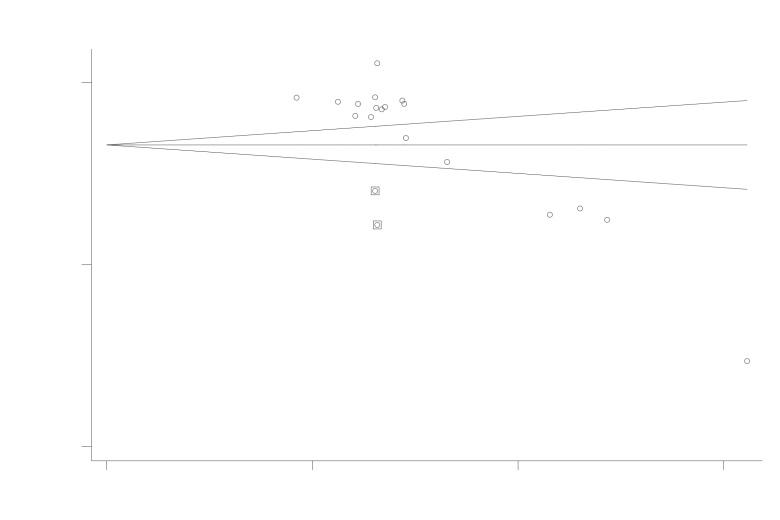


Figure 2. Trim-and-fill analyze of TNF-a


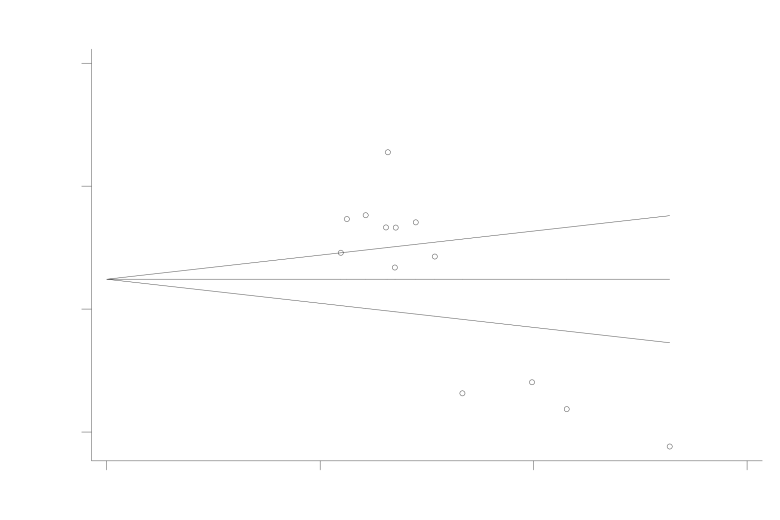


Figure 3. Trim-and-fill analyze of IL-6

**Supplementary Material 4：RoB informed sensitivity analysis**

**
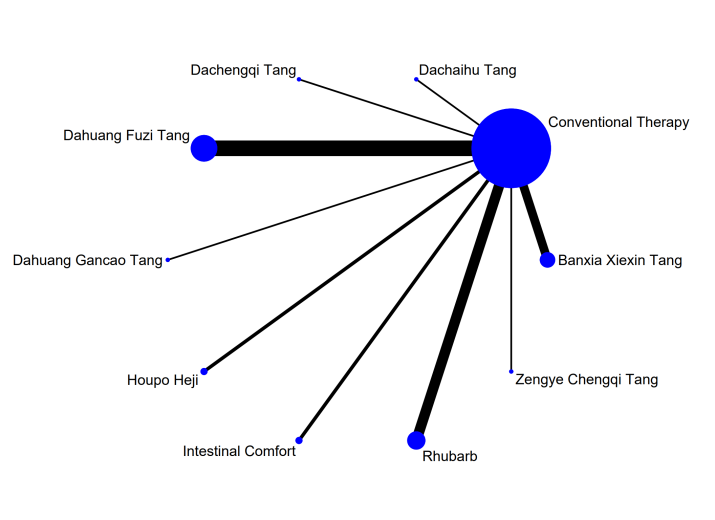
**

Figure 1. NMA figure

**
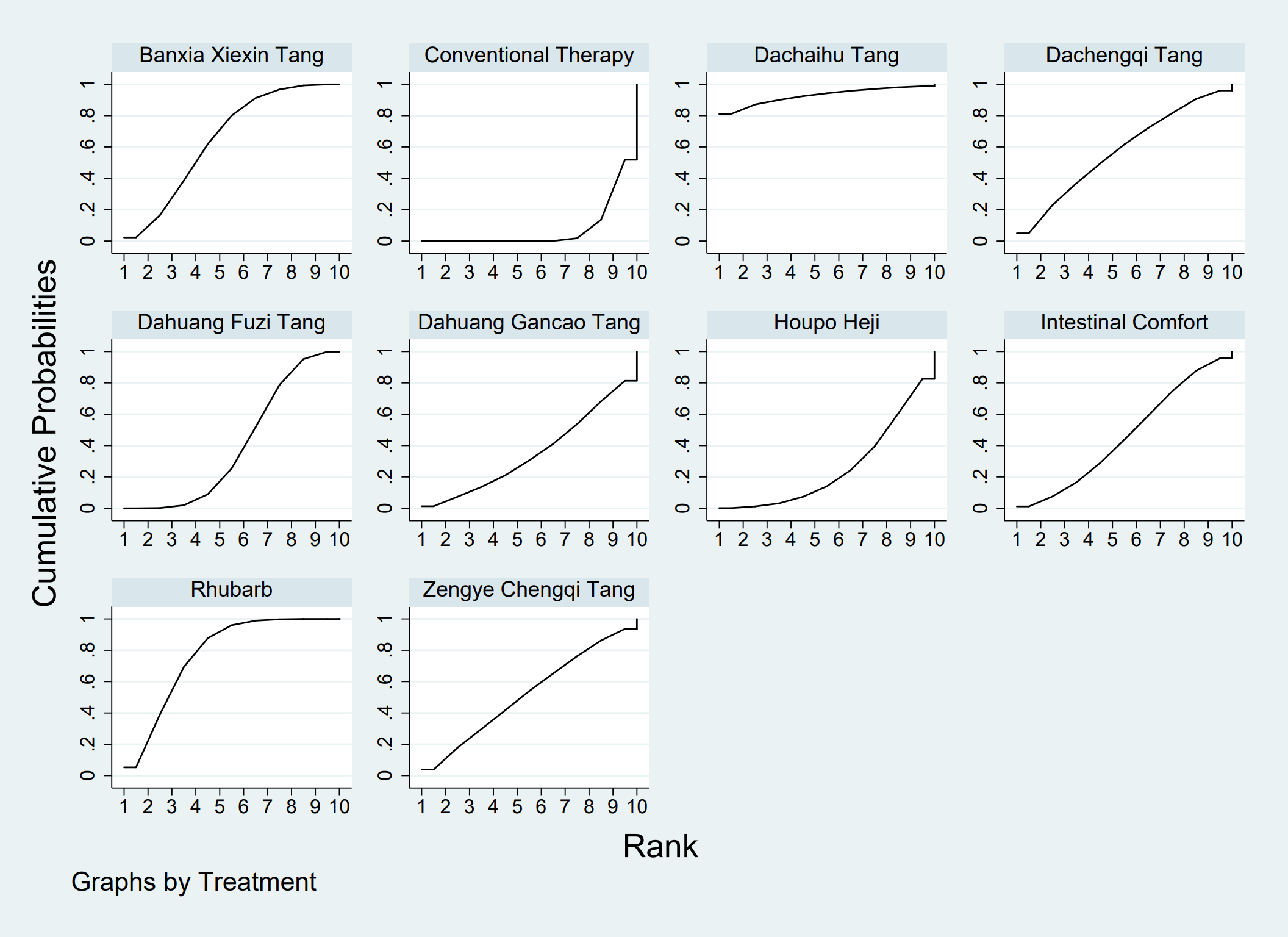
**

Figure 2. SUCRA plot

**
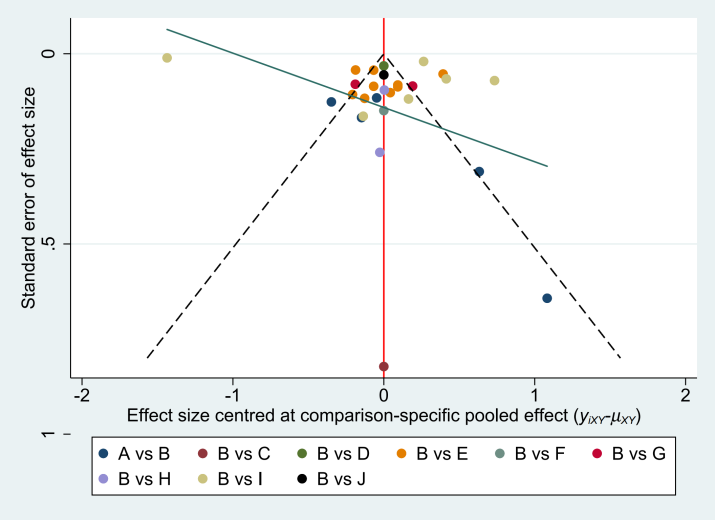
**

Figure 3. Funnel plots on publication bias

| Treatment | SUCRA | PrBest | MeanRank |
| --- | --- | --- | --- |
| Banxia Xiexin Tang | 65.2 | 2.2 | 4.1 |
| Conventional Therapy | 7.5 | 0.0 | 9.3 |
| Dachaihu Tang | 92.8 | 81.1 | 1.7 |
| Dachengqi Tang | 57.4 | 4.9 | 4.8 |
| Dahuang Fuzi Tang | 40.3 | 0.0 | 6.4 |
| Dahuang Gancao Tang | 35.4 | 1.3 | 6.8 |
| Houpo Heji | 25.9 | 0.1 | 7.7 |
| Intestinal Comfort | 46.2 | 1.2 | 5.8 |
| Rhubarb | 77.3 | 5.3 | 3.0 |
| Zengye Chengqi Tang | 52.0 | 3.8 | 5.3 |

Table 1. Cumulative probability table

| Dachaihu Tang | Rhubarb | Banxia Xiexin Tang | Dachengqi Tang | Zengye Chengqi Tang | Intestinal Comfort | Dahuang Fuzi Tang | Dahuang Gancao Tang | Houpo Heji | Conventional Therapy |
| --- | --- | --- | --- | --- | --- | --- | --- | --- | --- |
| Dachaihu Tang | 1.06 (-0.82,2.95) | 1.23 (-0.67,3.14) | 1.32 (-0.73,3.37) | 1.40 (-0.66,3.46) | 1.49 (-0.48,3.46) | 1.55 (-0.32,3.42) | 1.67 (-0.40,3.74) | 1.78 (-0.18,3.74) | 2.03 (0.18,3.88) |
| -1.06 (-2.95,0.82) | Rhubarb | 0.17 (-0.43,0.77) | 0.26 (-0.72,1.23) | 0.34 (-0.64,1.32) | 0.42 (-0.36,1.21) | 0.49 (0.01,0.97) | 0.61 (-0.41,1.62) | 0.72 (-0.03,1.46) | 0.97 (0.59,1.34) |
| -1.23 (-3.14,0.67) | -0.17 (-0.77,0.43) | Banxia Xiexin Tang | 0.09 (-0.93,1.10) | 0.17 (-0.85,1.19) | 0.25 (-0.58,1.08) | 0.32 (-0.23,0.88) | 0.44 (-0.62,1.49) | 0.55 (-0.25,1.34) | 0.80 (0.33,1.26) |
| -1.32 (-3.37,0.73) | -0.26 (-1.23,0.72) | -0.09 (-1.10,0.93) | Dachengqi Tang | 0.08 (-1.20,1.36) | 0.17 (-0.97,1.30) | 0.23 (-0.72,1.19) | 0.35 (-0.96,1.66) | 0.46 (-0.65,1.57) | 0.71 (-0.19,1.61) |
| -1.40 (-3.46,0.66) | -0.34 (-1.32,0.64) | -0.17 (-1.19,0.85) | -0.08 (-1.36,1.20) | Zengye Chengqi Tang | 0.09 (-1.05,1.22) | 0.15 (-0.80,1.11) | 0.27 (-1.04,1.58) | 0.38 (-0.73,1.49) | 0.63 (-0.28,1.54) |
| -1.49 (-3.46,0.48) | -0.42 (-1.21,0.36) | -0.25 (-1.08,0.58) | -0.17 (-1.30,0.97) | -0.09 (-1.22,1.05) | Intestinal Comfort | 0.07 (-0.68,0.82) | 0.18 (-0.99,1.35) | 0.29 (-0.65,1.24) | 0.54 (-0.14,1.23) |
| -1.55 (-3.42,0.32) | -0.49 (-0.97,-0.01) | -0.32 (-0.88,0.23) | -0.23 (-1.19,0.72) | -0.15 (-1.11,0.80) | -0.07 (-0.82,0.68) | Dahuang Fuzi Tang | 0.12 (-0.88,1.11) | 0.23 (-0.49,0.94) | 0.48 (0.17,0.78) |
| -1.67 (-3.74,0.40) | -0.61 (-1.62,0.41) | -0.44 (-1.49,0.62) | -0.35 (-1.66,0.96) | -0.27 (-1.58,1.04) | -0.18 (-1.35,0.99) | -0.12 (-1.11,0.88) | Dahuang Gancao Tang | 0.11 (-1.04,1.26) | 0.36 (-0.59,1.31) |
| -1.78 (-3.74,0.18) | -0.72 (-1.46,0.03) | -0.55 (-1.34,0.25) | -0.46 (-1.57,0.65) | -0.38 (-1.49,0.73) | -0.29 (-1.24,0.65) | -0.23 (-0.94,0.49) | -0.11 (-1.26,1.04) | Houpo Heji | 0.25 (-0.40,0.90) |
| -2.03 (-3.88,-0.18) | -0.97 (-1.34,-0.59) | -0.80 (-1.26,-0.33) | -0.71 (-1.61,0.19) | -0.63 (-1.54,0.28) | -0.54 (-1.23,0.14) | -0.48 (-0.78,-0.17) | -0.36 (-1.31,0.59) | -0.25 (-0.90,0.40) | Conventional Therapy |

Table 2. League table

| Side | Direct |  | Indirect |  | Difference |  |  | tau |
| --- | --- | --- | --- | --- | --- | --- | --- | --- |
|  | Coef. | Std. Err. | Coef. | Std. Err. | Coef. | Std. Err. | P>\|z\| |  |
| A B * | 0.7976086 | 0.2 | 0.0 | 66.0 | 0.7963597 | 66.00639 | 0.99 | 0.4589785 |
| B C * | -2.03 | 0.9 | -3.6 | 632.3 | 1.595174 | 632.2653 | 0.998 | 0.4589784 |
| B D * | -0.71 | 0.5 | -2.3 | 635.1 | 1.595201 | 635.1088 | 0.998 | 0.4589779 |
| B E * | -0.4756922 | 0.2 | -2.1 | 211.2 | 1.593533 | 211.2385 | 0.994 | 0.458978 |
| B F * | -0.36 | 0.5 | -2.0 | 632.9 | 1.595275 | 632.8915 | 0.998 | 0.4589779 |
| B G * | -0.2503411 | 0.3 | -1.8 | 445.2 | 1.596447 | 445.1988 | 0.997 | 0.4589779 |
| B H * | -0.5432501 | 0.4 | -2.2 | 435.7 | 1.608884 | 435.714 | 0.997 | 0.4589785 |
| B I * | -0.9667516 | 0.2 | -2.6 | 258.9 | 1.595111 | 258.8895 | 0.995 | 0.458978 |
| B J * | -0.63 | 0.5 | -2.2 | 622.7 | 1.580164 | 622.6768 | 0.998 | 0.4589779 |

Table 3. Node-specific
